# Human Milk Feeding in Inherited Metabolic Disorders: A Systematic Review of Growth, Metabolic Control, and Neurodevelopment Outcomes

**DOI:** 10.1002/jimd.70001

**Published:** 2025-02-06

**Authors:** Fatma Ilgaz, Alexander Höller, Cyril Marsaux, Sandra Banta‐Wright, Turgay Coşkun, Kelly A. Dingess, Monika Jörg‐Streller, Camille Newby, Rani Singh, Bernd Stahl, Clare Szwec, Annemiek van Wegberg, Willie Woestenenk, Anita MacDonald, Daniela Karall

**Affiliations:** ^1^ Department of Nutrition and Dietetics, Faculty of Health Sciences Hacettepe University Ankara Turkey; ^2^ Division of Nutrition and Dietetics University Hospital Innsbruck Innsbruck Austria; ^3^ Institute of Public Health, Medical Decision Making and Health Technology Assessment, Department of Public Health, Health Services Research and Health Technology Assessment UMIT TIROL‐University for Health Sciences and Technology Hall in Tirol Austria; ^4^ Digital Health Information Systems, Center for Health & Bioresources, AIT Austrian Institute of Technology Graz Austria; ^5^ Danone Research & Innovation Utrecht the Netherlands; ^6^ School of Nursing Oregon Health & Science University Portland Oregon USA; ^7^ Department of Pediatric Metabolism and Nutrition Hacettepe University Faculty of Medicine Ankara Turkey; ^8^ Department of Nutrition and Dietetics Bristol Royal Hospital for Children Bristol UK; ^9^ Department of Human Genetics and Pediatrics Emory University School of Medicine Atlanta Georgia USA; ^10^ Department of Chemical Biology & Drug Discovery Utrecht Institute for Pharmaceutical Sciences, Utrecht University Utrecht the Netherlands; ^11^ Division of Metabolic Diseases Beatrix Children's Hospital, University Medical Centre Groningen, University of Groningen Groningen the Netherlands; ^12^ Department of Gastroenterology and Hepatology‐Dietetics Radboud University Medical Center Nijmegen the Netherlands; ^13^ Department of Dietetics Birmingham Women's and Children's Hospital Birmingham UK; ^14^ Department of Pediatrics I, Division of Inherited Metabolic Disorders Medical University of Innsbruck Innsbruck Austria

**Keywords:** breast milk, breastfeeding, inborn errors of metabolism, PKU, rare metabolic diseases

## Abstract

Human milk (HM) is the optimal source of nutrition for infants. Yet the suitability of HM macronutrient composition, paired with the challenge of regulating HM intake, may deserve some consideration for infants with inherited metabolic disorders (IMDs) requiring restrictive and controlled dietary management. Except for classic galactosemia, HM feeding is expected to be feasible, allowing infants to maintain metabolic stability, while growing and developing optimally. However, information about HM feeding in nonphenylketonuria (PKU) literature is scarce. In this systematic review, 52 studies were included, representing 861 infants (86% PKU) receiving HM after IMD diagnosis (mean duration 4–10 months depending on the IMD). For non‐PKU IMDs (e.g., other amino acidopathies, urea cycle disorders, organic acidemias, fatty acid oxidation disorders), outcomes of HM feeding were available for few infants, except for medium‐chain acyl‐CoA dehydrogenase (MCAD) deficiency (*n* = 48). In PKU, HM feeding combined with phenylalanine‐free formula, led to adequate metabolic control (25 studies), growth (15 studies), and neurodevelopment (10 studies). For other IMDs, more evidence is required, but the limited data suggest that HM feeding is possible, with attentive monitoring and disease‐specific formula supplementation where applicable. In MCAD deficiency, ensuring adequate HM intake is essential, as symptoms were more frequently reported in exclusively breastfed infants. No IMD‐specific articles were found on the relationship between HM feeding and many other outcomes of interest (e.g., immune status or comorbidity risk later in life). With the exception of galactosemia, HM feeding is expected to benefit infants with IMD. More data should be published for IMDs other than PKU.

## Graphical abstract

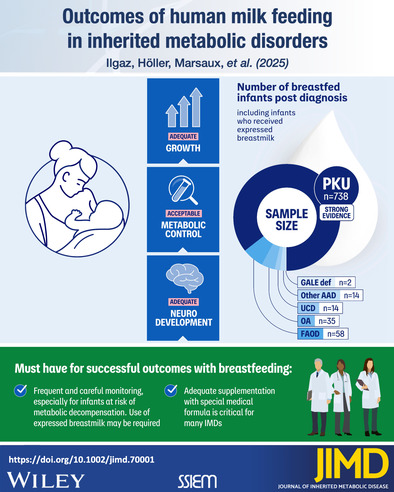

## 1 Introduction

Inherited metabolic disorders (IMDs) are rare genetic defects of specific enzymes causing a block in the metabolism of amino acids, carbohydrates, or fatty acids. This may result in intoxication (i.e., toxic accumulation, in plasma and tissues, of substrates and alternative products before the metabolic block, together with a possible deficiency of products after the block in the pathway), disturbances of energy metabolism, and/or mishandling of complex large molecules [1]. If left untreated, IMDs may lead to severe multi‐system outcomes including irreversible neurological damage, neurodevelopmental deficits, metabolic encephalopathy, kidney, liver, eye, skin or bone dysfunction, faltering growth, or even death [2, 3]. Some of these disorders can be treated by dietary interventions with restriction or avoidance of the “offending” precursor nutrients and supplementation with any deficient products as necessary. Sometimes, this is combined with appropriate pharmaceutical therapy. To achieve metabolic control and to prevent metabolic decompensation, lifelong dietary treatment should commence immediately following diagnosis in the neonatal period [1, 4].

Human milk (HM), with its dynamic composition and high nutrient utilization, is universally recognized as the best source of nutrition for (healthy) infants [5]. HM contains approximately 88% water, 6% lactose, 4% lipids (including essential fatty acids), 1% protein, and micronutrients, as well as an abundance of key functional components, for example, HM oligosaccharides (HMOs), maternal immune cells, lactoferrin, secretory immunoglobulin A, cytokines, nucleotides, exosomes, free amino acids, creatine, urea, and beneficial bacteria [5, 6, 7, 8, 9, 10, 11, 12, 13, 14, 15, 16, 17, 18, 19, 20]. In the past decades, a wealth of data has confirmed that HM feeding (HMF) is a major determinant of optimal infant and child health. Many acute and chronic pediatric disorders including otitis media, acute diarrheal disease, lower respiratory illnesses, sudden infant death syndrome, inflammatory bowel disease, childhood leukemia, diabetes mellitus, obesity, asthma, and atopic dermatitis, occur less frequently among children who were breastfed as infants (Table 1) [26, 28, 29, 30, 31, 46, 77, 78]. In addition to the advantages for the growing infant, there are also benefits for mothers who breastfeed (e.g., reduced risk of reproductive cancers and cardiovascular diseases, positive influence on mental health, etc.) (Table 1) [59, 61, 79]. Although the benefits in the non‐IMD population are well described, there are few published reports of HMF in patients with IMDs (except for phenylketonuria [PKU]) [80].

**TABLE 1:** Short‐ and long‐term benefits/advantages of human milk feeding.

Benefits/advantages of human milk feeding
Infancy and early childhood:
High nutrient utilization [5, 21]
Presence of functional proteins and enzymes that facilitate nutrient digestion (e.g., bile salt‐stimulated lipase) and uptake (e.g., lactoferrin, α‐lactalbumin)
Fatty acid profile (softer stool, better digestion and absorption, maturation of visual system)
Benefits of lactose over maltodextrin in infant formulas
Lower osmolality
Optimal growth and maturation [22, 23]
Reduced incidence of sudden infant death syndrome [24]
Better child visual motor ability and higher Intelligence Quotient at school age with longer exclusive human milk feeding duration [25, 26]
Decreased risk of infectious disease (e.g., otitis media, gastroenteritis, respiratory tract infections), as well as diabetes mellitus, sepsis, and necrotizing enterocolitis [27, 28, 29, 30, 31]
Immunologic components including probiotics, prebiotics (e.g., human milk oligosaccharides), immunoglobulins [32]
Protection against childhood asthma and allergic rhinitis [33, 34, 35, 36, 37, 38]
Better food acceptance during weaning and childhood [39, 40]
Fewer dental malocclusions [41]
Reduced risk of childhood leukemia [42]
Late childhood, adolescence and adulthood:
Moderate protection against overweight and obesity in childhood, adolescence and adulthood with a dose–response effect between duration and exclusivity of breastfeeding and risk reduction [43, 44, 45]
Protection against type 2 diabetes [46]
Protection against metabolic syndrome [47]
Greater performance on intelligence tests during childhood and adolescence [48]
Maternal benefits:
Helps with post‐partum weight loss [49, 50, 51, 52, 53, 54, 55, 56]
Lower risk of maternal type 2 diabetes [57, 58]
Lower risk of cardiovascular disease [59, 60]
Lower risk of breast, ovarian, endometrial and thyroid cancer [61, 62, 63]
Lower risk of hemorrhages, prolonged amenorrhea [61]
Psychological benefits, for example, enhanced mother‐infant emotional bonding [64, 65, 66]
Additional/future benefit areas of human milk research:
Benefit of human milk oligosaccharides on, for example, cognition and behavior [67, 68]
Microbiota and dysbiosis (e.g., higher levels of beneficial gut bacteria) [69]
Milk fat globule membrane (MFGM) [21, 70]
MicroRNAs [71]
Chrononutrition [72]
Systems biology [73]
Oral health [74, 75, 76]

Historically, when an IMD of amino acid metabolism was diagnosed in early infancy, HMF was stopped and replaced with a premeasured volume of standard infant formula, combined with a disease‐specific medical formula. This was considered the most effective, safest, and easiest method allowing for accurate titration and estimation of nutrient intakes [81]. Probably the first successful experience with HMF was described in PKU in the early 1980s [82]. Determination of the relatively low phenylalanine (Phe) content in HM [83] enabled its use in combination with a Phe‐free medical formula as a feasible alternative to exclusive formula feeding [84, 85, 86, 87], and today, HMF is recommended by management guidelines for PKU [88, 89].

In classic galactosemia [90], HMF is clearly contra‐indicated because toxic analytes (e.g., erythrocyte galactose‐1‐phosphate and urinary galactitol) would accumulate, leading to life‐threatening complications in untreated infants [91]. However, for the majority of other IMDs, it is expected that HMF is feasible, if it can be partially or fully incorporated into the prescribed dietary regimens, so that infants maintain metabolic stability and attain optimum nutritional intake to enable acceptable growth and development. To confirm this, we systematically reviewed the literature on outcomes with HMF in infants with IMDs where management requires a therapeutic dietary intervention (e.g., protein/amino acid or fat restriction), and where HMF might be considered a risk factor for precipitating metabolic instability. Future initiatives should also focus on current clinical practices and provide recommendations on how to best support HMF of infants with IMDs.

## 2 Materials and Methods

The Preferred Reporting Items for Systematic Reviews and Meta‐Analyses (PRISMA) guideline was followed [92], and the review protocol was published on the PROSPERO international prospective register of systematic reviews (CRD42024598922; www.crd.york.ac.uk/PROSPERO, accessed on November 4, 2024).

### 2.1 Literature Search

A systematic literature search was conducted in 97 electronic databases available on ProQuest Dialog (including Medline, Embase, SciSearch and BIOSIS Previews) without any publication date restriction. Only articles or (conference) abstracts published in English were included. The last search was completed on July, 24, 2024. The reference lists of studies selected for inclusion, as well as previous reviews or relevant published guidelines were also checked for any additional relevant references that might not have been captured by the electronic search.

The final search string is provided in Table S1. The search strategy included a combination of different groups of keywords that described the main condition (e.g., “inborn errors of metabolism” OR “inherited metabolic disease/disorders” OR “rare metabolic disease/disorders”), and exposure (e.g., “breastfeeding” OR “human/breast/maternal/mother milk”). Additional keywords relating to IMDs associated with aminoacidopathies, organic acidemias, urea cycle disorders, glycogen storage disease, fatty acid oxidation disorders and others, where HMF might be considered a risk, were also included in the search string (Table S1). A full list of the electronic databases searched can be accessed via the following link: https://dialog.com/commercialdatabases/.

### 2.2 Study Selection

The PICO (population, intervention, comparison, outcomes) method was applied to formulate the review question. Any IMDs where management requires a diet modification were considered in scope, except for galactosemia, which was excluded from this review because HM feeding is unequivocally contra‐indicated for this disorder. To ensure all relevant data were captured, no restriction was used for the study type or design. Original research was considered eligible if it provided information on HMF in infants with IMD. Conference abstracts with relevant data were also included in the absence of a full paper.

Screening was carried out by two independent reviewers (F.I. and A.H.). Any disagreements were resolved through discussion with the other authors.

### 2.3 Outcome Measures and Data Extraction

Data reported for infants receiving HM and/or their mothers were extracted, as well as data for any available comparison group (e.g., infants not receiving HM). Mainly, this included data on the following outcomes: (1) physical growth (e.g., weight, length/height, head circumference), (2) metabolic control (e.g., blood or urine levels of specific biomarkers), (3) neurodevelopmental status (e.g., screening of neurodevelopmental performance, achievement of developmental milestones), (4) nutritional status (e.g., blood concentrations or intakes of macro and micronutrients), (5) immune status (e.g., frequency of infections, hospitalization) as well as (6) some outcomes related to the mothers of infants with IMD (e.g., maternal health, maternal stress, etc.). Any reported adverse events were also extracted.

Data were collected by two independent authors (F.I. and A.H.) using a standardized data extraction form and were checked by a third author (C.M.). Information extracted included (1) study characteristics (authors, publication year, country, setting, and study design), (2) description of the population (sample size, clinical diagnosis/phenotype, sex, age at initiation and cessation of HMF, and ethnicity), (3) description of the exposure (disease‐specific dietary treatment, strategies/protocol to implement HMF including mode of administration, duration of HMF, and protocol for nutritional and metabolic monitoring), and (4) outcomes.

### 2.4 Data Analysis

Data obtained from the included studies were synthesized in a qualitative manner. There were differences between studies in terms of presentation or reporting of data for each outcome. Furthermore, data were restricted to only a few cases for most IMDs with limited quantitative data on the outcomes of interest. Hence, a meta‐analysis was not performed.

### 2.5 Quality Appraisal and Risk of Bias Assessment

Two reviewers (F.I. and C.M.) independently assessed the quality of the evidence and the risk of bias of the included studies using the “NIH (National Institutes of Health) Study Quality Assessment Tools” developed jointly by the U.S. National Heart, Lung and Blood Institute (NHLBI, National Institutes of Health) and Research Triangle Institute (RTI) International [93]. Three different NIH Quality Assessment Tools were used depending on the study design: Quality Assessment of Controlled Intervention Studies (14 items), Quality Assessment Tool for Observational Cohort and Cross‐Sectional Studies (14 items), and Quality Assessment Tool for Case Series (9 items). Each tool comprises 9 to 14 items to evaluate potential flaws in study methods or implementation, including sources of bias (e.g., patient selection, performance, attrition, and detection), confounding, study power, and the strength of causality in the association between interventions and outcomes. Considering the detailed guidance document specific to each tool, reviewers selected “yes,” “no,” “cannot determine,” “not reported,” or “not applicable” in response to each item. Based on the ratings, an overall judgment was made regarding the quality of each study: (1) “good quality” if the study had minimal risk of bias, (2) “fair quality” if the study was susceptible to some bias but not deemed sufficient to invalidate its results, and (3) “poor quality” if the study raised substantial concerns. Differing ratings between reviewers were discussed until consensus was reached.

## 3 Results

### 3.1 Study Selection

**FIGURE 1:**
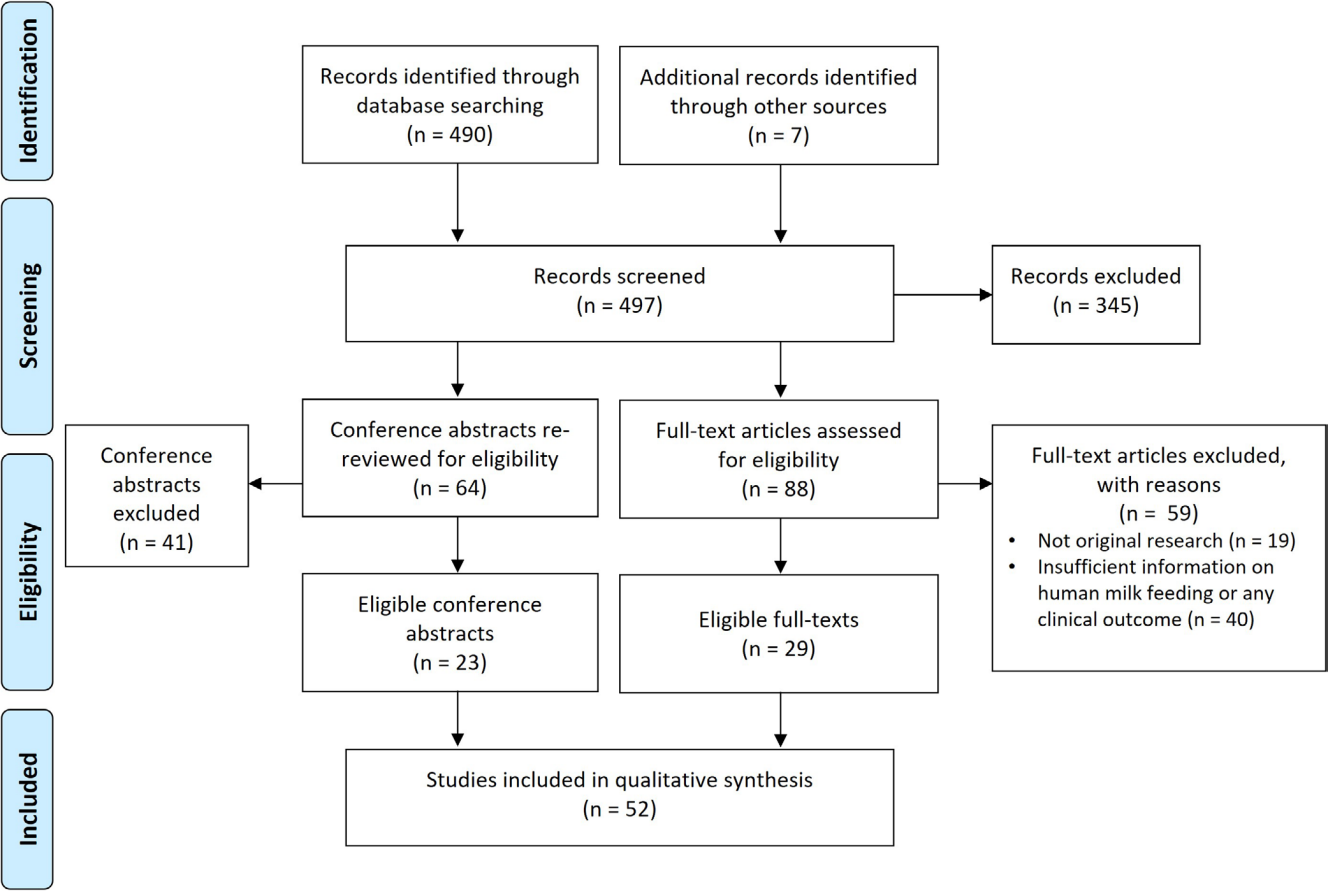
Study selection process according to the Preferred Reporting Items for Systematic Reviews and Meta‐Analysis (PRISMA) flow chart. *Note:* Five of 88 full‐text articles retrieved were not in English (the corresponding abstracts were in English, which is how they were captured by the electronic search). It was decided to assess their eligibility after translation, after which two were included.

A total of 52 studies (23 [44%] as conference abstracts only) reported their experience with HMF in IMD and were included in the systematic review (Figure 1): 23 case reports/case series, 21 observational cohort/cross‐sectional studies, and 8 nonrandomized controlled studies. Studies were conducted in Europe (*n =* 23, including *n* = 7 in the UK), the United States (*n =* 12), Turkey (*n =* 4), Brazil (*n =* 4), Australia (*n =* 4), Canada (*n* = 1), Chile (*n =* 1), India (*n* = 1), Iran (*n* = 1), and Mexico (*n* = 1) (Table S2).

### 3.2 Subject Characteristics

We included data from 861 infants receiving HM after IMD diagnosis, as well as data from 705 infants fed exclusively standard infant formula post diagnosis (total: 1566 infants with an IMD) (Tables S2 and S3). Most infants receiving HM after diagnosis had an amino acid (AA) disorder (*n =* 752/861, 87%, 38 studies), mainly PKU (*n =* 738 [including few cases of hyperphenylalaninemia, HPA], 86%, 30 studies). Some infants had a fatty acid oxidation disorder (FAOD; *n =* 58, 7%, 7 studies), and only a limited number had an organic acidemia (OA, *n =* 35, 4%, 6 studies), urea cycle disorder (UCD; *n =* 14, 2%, 7 studies) or other disorder (galactose epimerase [GALE] deficiency: only one study with 2 HM‐fed infants) (Tables S2 and S3).

Most infants were diagnosed within the first month of life, mainly by newborn screening. Seven of 35 children with OAs were “late” diagnosed, between 3 months and 2.5 years (*n =* 3 methylmalonic acidemia [MMA]; *n =* 1 propionic acidemia [PA]; *n =* 2 glutaric aciduria type 1 [GA1]; and *n =* 1 malonic acidemia [MA]), as well as one of 14 children with a UCD (*n =* 1 ornithine transcarbamylase [OTC] deficiency, diagnosed at 1.5 years), and 2 of 58 children with FAODs (*n =* 2 medium‐chain acyl‐coenzyme A dehydrogenase [MCAD] deficiency, diagnosed at 2 months and 3.5 years). Most infants with PKU had the classical phenotype. For other IMDs, phenotypic information was commonly not reported, but when the information was available for OAs and UCDs, infants had severe phenotypes. For FAOD, HM‐fed infants with long‐chain hydroxyacyl‐coA dehydrogenase (LCHAD) or very long‐chain acyl‐CoA dehydrogenase (VLCAD) deficiency were diagnosed by newborn screening. They were either asymptomatic at diagnosis (*n* = 4) or this information was not reported (*n* = 4). The two infants with carnitine‐acylcarnitine translocase (CACT) or carnitine palmitoyltransferase II (CPTII) deficiency had a severe phenotype, and 27% of HM‐fed infants with MCAD deficiency were symptomatic at diagnosis (*n =* 13/48) (Table S2).

### 3.3 Summary of HMF Practices in IMDs After Diagnosis

After diagnosis, exclusive HMF was reported in 64 HM‐fed infants: *n =* 37/48 (77%) HM‐fed infants with MCAD deficiency, *n =* 4/8 with LCHAD or VLCAD deficiency, *n =* 1/1 with CPTII deficiency, *n =* 3/4 with OTC deficiency, *n =* 2/2 with arginase deficiency, *n =* 1/2 with CPS1 deficiency, *n =* 2/2 with GALE deficiency, *n =* 8/14 with MMA, *n =* 4/12 with PA, *n =* 1/1 with MA, and *n =* 1/1 with hypermethioninemia (Table S3). However, for almost all HM‐fed infants with AA disorders (*n* = 750/752) and most infants with OAs (*n* = 22/35), the feeding regimen was partial HM in combination with a disease‐specific medical formula. The most common feeding approaches were to give a prescribed amount of medical formula prior to on demand breastfeeding or to alternate on demand breastfeeding and medical formula feeds. Two studies reported that standard infant formula was necessary in addition to breastmilk and medical formula for *n =* 29/54 infants with PKU, due to insufficient breastmilk supply [94, 95]. The use of controlled volumes of expressed HM, mixed with medical formula where required, was common after acute management of symptomatic infants with MSUD (*n =* 3/4 studies), OAs (*n =* 5/6 studies), UCDs (*n =* 6/6 studies), and FAODs (*n =* 4/5 studies), before trying to establish on demand breastfeeding (Table S3).

The mean duration of HMF was 4 to 10 months across IMDs, though some mothers were still breastfeeding at the last follow‐up, and for some studies, HMF duration was not reported. In all IMDs, HMF duration varied widely depending on the infant/mother ranging from 1 week to 26 months in AA disorders, from 1.5 to 24 months in OAs, from 1.5 to 9 months in UCDs, and from one to > 12 months in FAODs (Table S3).

### 3.4 Overview of Study Results

Key findings for each disorder and each outcome are summarized in Tables 2, 3, 4, 5, 6, with additional details provided in Tables S4–S7. Growth, metabolic control and neurodevelopment were evaluated in 30, 46, and 21 of 52 studies, respectively, while only six studies reported limited information on the effects of HMF (or HMF cessation) on the psychological and mental health of mothers of infants with an IMD or the mother–infant relationship [82, 96, 102, 127, 139, 140]. Biochemical assessment of nutritional status was rarely studied. Data on energy and nutrient intakes were not described in the majority of studies and, if reported, were so heterogenous that they were not included in summary tables. No studies evaluated immune status. Furthermore, we were unable to identify any articles that investigated the relationship between HMF and dental health, the risk of childhood asthma, allergic rhinitis, or cancers, or the risk of obesity or chronic diseases in later life.

**TABLE 2:** Growth, metabolic control, and neurodevelopment in infants with phenylketonuria who received human milk after diagnosis.

Condition	References	HM‐fed infants	Mean HMF duration (months)	Growth	Metabolic control	Neurodevelopment
*Phenylketonuria*	Francis (1981) [82]	6	n/r	n/r	Acceptable	n/r
	McCabe (1989) [86]	18	9	Adequate	Acceptable	n/r
	Greve (1994) [85]	9	≥ 4	Adequate	Acceptable	n/r
	Miller (1994) [96]	1	3	Adequate	Acceptable	Adequate
	Riva (1996) [97]	5	1	n/r	n/r	Adequate
	Duncan (1997) [84]	1	11	n/r	Acceptable	n/r
	Motzfeldt (1999) [87]	74	7	Adequate	Acceptable	n/r
	Davidson (2000) [98]	33	≤ 15	Adequate	Acceptable	Adequate
	Francis (2000) [99]	1	5	Adequate	Acceptable	Adequate
	Agostoni (2003) [100]	7	≤ 2	n/r	n/r	Adequate
	Cornejo (2003) [94]	19	≥ 6	Adequate	Acceptable (*n* = 15) Poor (*n* = 4)	Adequate (*n* = 11 or 13) Inadequate (*n* = 5 or 3) n/r (*n* = 3)
	van Rijn (2003) [101]	9	3 (median)	Adequate	Acceptable	n/r
	Nielsen (2005) [102]	6	n/r	n/r	Acceptable	n/r
	Kanufre (2007) [95]	35	8	Adequate	Acceptable	n/r
	Sweeney (2009) [103]	22	7 (median)	Adequate	Acceptable	n/r
	Santos (2011) [104]	39	≤ 6	Adequate	Acceptable	n/r
	Sweeney (2011) [105]	1	≥ 16	n/r	Acceptable	Adequate
	Banta‐Wright (2012) [106]	75	7	n/r	Acceptable	n/r
	Lamônica (2012) [107]	10	5	n/r	Acceptable (*n* = 8) Poor (*n* = 2)	Adequate (*n* = 8) Inadequate (*n* = 2)
	O'Sullivan (2013) [108]	45	n/r	n/r	Acceptable	n/r
	Sweeney (2016) [109]	1	10	n/r	Acceptable	Adequate
	Köse (2018) [110]	25	7	Adequate	Acceptable	n/r
	Weiss (2020) [111]	3	n/r	n/r	Acceptable	Adequate (*n* = 2) Inadequate (*n* = 1)
	Zuvadelli (2022) [112]	28	7	Adequate	Acceptable	n/r
	Rice (2023) [113]	16	8	n/r	Acceptable	n/r
	Rocha (2023) [114]	30	5 (median)	n/r	n/r	n/r
	Guillén‐López (2024) [115]	181	n/r	Adequate	Acceptable	n/r
	Mohammadzadeh (2024) [116]	13	14	Adequate	Acceptable	n/r

*Note:* More details can be found in the Supporting Information. Adequate/poor growth, acceptable/poor metabolic control, and adequate/inadequate neurodevelopment as defined by the original authors. Adequate growth was usually defined as body length and weight within 3rd–97th percentiles or *z*‐scores within two standard deviations; acceptable metabolic control was defined as metabolites in the age‐adapted therapeutic ranges; and for neurodevelopment, various questionnaires were used to assess mental and/or psychomotor development/acquisition of developmental milestones.

Abbreviations: HM, human milk; HMF, human milk feeding; *n*, number of infants; n/r, not reported.

**TABLE 3:** Growth, metabolic control, and neurodevelopment in infants with aminoacidopathies other than phenylketonuria who received human milk after diagnosis.

Condition	References	HM‐fed infants	HMF duration (months)	Growth	Metabolic control	Neurodevelopment
Maple syrup urine disease	Touati (2001) [117]	3	4, 10, n/r	Adequate	Acceptable	Adequate
	Huner (2005) [118]	1	3	Adequate	Acceptable	Adequate
	Ross (2016) [119]	1	n/r	n/r	Acceptable	n/r
	Pichler (2017) [120]	1	1	Adequate	Acceptable	Adequate
Homocystinuria	Dixon (2014) [121]	3	3 ≥ 6	Adequate	Poor (*n* = 1) Acceptable (*n* = 2)	n/r
	Aktuğlu (2015) [122]	1	≥ 18	n/r	Acceptable	n/r
Hypermethioninemia	Pichler (2017) [120]	1	12	Adequate	Acceptable	Adequate
Tyrosinemia	Daly (2005) [123]	2	5, 5	Adequate	Acceptable	n/r
	Silva (2022) [124]	1	13	Adequate	Acceptable	Adequate

*Note:* More details can be found in the Supporting Information. Adequate/poor growth, acceptable/poor metabolic control, and adequate/inadequate neurodevelopment as defined by the original authors. Adequate growth was usually defined as body length and weight within 3rd–97th percentiles or *z*‐scores within two standard deviations; acceptable metabolic control was defined as metabolites in the age‐adapted therapeutic ranges; and for neurodevelopment, various questionnaires were used to assess mental and/or psychomotor development/acquisition of developmental milestones.

Abbreviations: HM, human milk; HMF, human milk feeding; *n*, number of infants; n/r, not reported.

**TABLE 4:** Growth, metabolic control, and neurodevelopment in infants with organic acidemias who received human milk after diagnosis.

Condition	References	HM‐fed infants	HMF duration (months)	Growth	Metabolic control	Neurodevelopment
Methylmalonic acidemia	Dixon (2000) [125]	2	< 1 11	Poor (*n* = 1) Adequate (*n* = 1)	Poor (*n* = 1) Acceptable (*n* = 1)	Adequate
	Huner (2005) [118]; Gokcay (2006) [126]	4	19	Adequate	Acceptable	Adequate (*n* = 2) Inadequate (*n* = 2)
	Pichler (2017) [120]	2	9, 12	Adequate	Acceptable	Adequate
	Starin (2024) [127]	6	≥ 12	n/r	Acceptable	n/r
Propionic acidemia	Dixon (2000) [125]	1	≥ 4	Adequate	Acceptable	n/r
	Huner (2005) [118]; Gokcay (2006) [126]	1	4	Adequate	Poor	Adequate
	Pichler (2017) [120]	3	12	Adequate	Acceptable	Inadequate
	Starin (2024) [127]	7	≥ 8	n/r	Acceptable	n/r
Glutaric acidemia type 1	Huner (2005) [118]; Gokcay (2006) [126]	2	6, 11	Adequate^a^	Acceptable	Adequate (*n* = 1) Inadequate (*n* = 1)
	Fitzachary (2015) [128]	2	≥ 6	Adequate	Acceptable	n/r
	Pichler (2017) [120]	2	3 5	Adequate (*n* = 1) Poor (*n* = 1)	Acceptable	Adequate (*n* = 1) Inadequate (*n* = 1)
Isovaleric acidemia	Huner (2005) [118]; Gokcay (2006) [126]	2	2, 10	Adequate^a^	Acceptable	Adequate
Malonic acidemia	Pichler (2017) [120]	1	6	Adequate	Acceptable	Adequate

*Note:* More details can be found in the Supporting Information. Adequate/poor growth, acceptable/poor metabolic control, and adequate/inadequate neurodevelopment as defined by the original authors. Adequate growth was usually defined as body length and weight within 3rd–97th percentiles or *z*‐scores within two standard deviations; acceptable metabolic control was defined as metabolites in the age‐adapted therapeutic ranges; and for neurodevelopment, various questionnaires were used to assess mental and/or psychomotor development/acquisition of developmental milestones.

Abbreviations: HM, human milk; HMF, human milk feeding; n, number of infants; n/r, not reported.

^a^ Huner 2005 and Gokcay 2006: Glutaric acidemia type 1, adequate height and weight but both infants had a head circumference above normal. Isovaleric acidemia, one infant was obese at follow up.

**TABLE 5:** Growth, metabolic control, and neurodevelopment in infants with urea cycle disorders who received human milk after diagnosis.

Condition	References	HM‐fed infants	HMF duration (months)	Growth	Metabolic control	Neurodevelopment
Citrullinemia	Kamper (2001) [129]	1	≥ 8	Adequate	Acceptable	Adequate
Ornithine transcarbamylase deficiency	Rawlinson (2000) [130]	2	7, 8	n/r	Acceptable	n/r
	Huner (2005) [118]	1	2	n/r	Poor	n/r
	Pichler (2017) [120]	1	9	Adequate	Acceptable	Adequate
	Burfield (2024) [131]	1	3	Adequate	Acceptable	n/r
Arginase deficiency	Pichler (2017) [120]	2	2, 6	Adequate^a^	Acceptable	Adequate
Argininosuccinate lyase deficiency	Dixon (2000) [125]	2	3, ≥ 4	Adequate	Acceptable	Adequate
	Burfield (2024) [131]	2	≥ 7, ≥ 8	Adequate	Acceptable	n/r
Carbamoyl phosphate synthetase 1 deficiency	Bzduch (2019) [132]	2	2, 3	Adequate	n/r	Adequate

*Note:* More details can be found in the Supporting Information. Adequate/poor growth, acceptable/poor metabolic control, and adequate/inadequate neurodevelopment as defined by the original authors. Adequate growth was usually defined as body length and weight within 3rd–97th percentiles or *z*‐scores within two standard deviations; acceptable metabolic control was defined as metabolites in the age‐adapted therapeutic ranges; and for neurodevelopment, various questionnaires were used to assess mental and/or psychomotor development/acquisition of developmental milestones.

Abbreviations: HM, human milk; HMF, human milk feeding; *n*, number of infants; n/r, not reported.

^a^ Pichler 2017: Adequate height but underweight in one infant with arginase deficiency.

**TABLE 6:** Growth, metabolic control, and neurodevelopment in infants with fatty acid oxidation disorders who received human milk after diagnosis.

Condition	References	HM‐fed infants	HMF duration (months)	Growth	Metabolic control	Neurodevelopment
Medium‐chain acyl‐CoA dehydrogenase deficiency	Roe (1986) [133]	1	n/r	n/r	Acceptable	n/r
	Hsu (2008) [134]	11	n/r	n/r	Mixed^a^	Adequate (*n* = 10) Inadequate (*n* = 1)
	Ahrens‐Nicklas (2016) [135]	34	n/r	n/r	Mixed^a^	n/r
	Petropoulou (2017) [136]	1	2	Poor	Acceptable	n/r
Long‐chain hydroxyacyl‐coA dehydrogenase (LCHAD) deficiency	Pichler (2017) [120]	3	2.5, 3 5	Adequate (*n* = 2) Poor (*n* = 1)^b^	Acceptable	Adequate
Very long‐chain acyl‐CoA dehydrogenase (VLCAD) deficiency	Pichler (2017) [120]	1	1	Adequate	Acceptable	Adequate
Mixed (LCHAD or VLCAD)	Hussa (2006) [137]	4	≤ 4	Adequate	Acceptable	Adequate
Carnitine‐acylcarnitine translocase deficiency	Kritzer (2020) [138]	1	> 12	Adequate	Acceptable	Adequate
Carnitine palmitoyltransferase II deficiency	Pichler (2017) [120]	1	n/r	Adequate	Acceptable	Adequate

*Note:* More details can be found in the Supporting Information. Adequate/poor growth, acceptable/poor metabolic control, and adequate/inadequate neurodevelopment as defined by the original authors. Adequate growth was usually defined as body length and weight within 3rd–97th percentiles or *z*‐scores within two standard deviations; acceptable metabolic control was defined as metabolites in the age‐adapted therapeutic ranges; and for neurodevelopment, various questionnaires were used to assess mental and/or psychomotor development/acquisition of developmental milestones.

Abbreviations: HM, human milk; HMF, human milk feeding; *n*, number of infants; n/r, not reported.

^a^ Hsu 2008: Initial C8 concentrations were significantly higher in breastfed infants versus infants on standard formula, but no difference at follow up. Ahrens‐Nicklas 2016: C8 concentrations were significantly higher in exclusively breastfed infants vs. infants on standard formula; 11 of the 24 (46%) exclusively breastfed infants had signs of metabolic decompensation.

^b^ Pichler 2017: Poor linear growth but adequate weight in one infant with LCHAD deficiency.

Considering growth outcome, 20/30 studies measured weight and/or length/height, head circumference was evaluated in nine studies, and 10/30 studies did not specify how they assessed growth. Growth was usually assessed weekly, biweekly or monthly between 0 and 6 months of age, and less frequently (monthly or every 2–3 months) thereafter. No apparent difference was observed in the frequency of growth monitoring according to the type of disorder. However, there was a high heterogeneity between studies for reporting growth data (e.g., percentiles, *z*‐scores, or actual measurements). The studies usually used cut‐off values of ±2 standard deviations, or < 3rd and > 97th percentiles to define abnormal growth, which is similar to the recommendations from the World Health Organization [141].

Two‐thirds of the studies (*n =* 14/21) did not describe which method was used to evaluate neurodevelopmental status. The Denver Developmental Screening Test II (DDST II) [142] was used in three studies [118, 120, 126], the Bayley Mental Developmental Index (MDI) and Psychomotor Developmental Index (PDI) [143] in two studies [94, 100], whereas the Wechsler Intelligence Scales for Children (WISCR‐adapted for 6–16 years of age) [144], the Early Language Milestone Scale [145], and the Basic Steps of Development [146], were each used in one study [97, 107].

#### 3.4.1 Amino Acid Disorders—PKU

##### 3.4.1.1 Growth

Fifteen studies evaluated growth in 507 HM‐fed infants with PKU (Tables 2 and S4). For five studies, data could only be retrieved from abstracts. HMF combined with Phe‐free medical formula led to adequate growth in all studies (i.e., > −2SD or > 3rd centile, within the normal ranges for age of national growth charts, or the definition was not reported).

When comparing HM‐fed versus standard infant formula‐fed infants (*n =* 10 studies), two studies with 1‐year follow‐up reported a statistically significant difference in weight gain between the two groups. Köse et al. [110] found that both weight gain and absolute weight were lower at 6 months, but higher at the end of 1 year in the HMF group, in line with the WHO growth curves for breastfed infants. Zuvadelli et al. [112] reported a significantly higher weight gain in the standard infant formula‐fed group during the first year of life, although the length growth rate was similar between groups. No statistically significant difference in growth parameters between groups was found in the other eight studies (Table S4).

In one study, the relationship between type of HMF administration (i.e., premeasured HMF via bottle, on demand HMF after metabolic formula, time‐controlled HMF or alternate infant formula/HMF) and growth was investigated [112], but no significant differences were observed.

##### 3.4.1.2 Biochemical Assessment of Nutritional Status

Only two studies reported on the nutritional status of HM‐fed infants with PKU. One prospective study did not find any significant differences between HM‐fed and standard infant formula‐fed infants for hemoglobin, hematocrit, serum iron, total iron binding capacity, iron saturation (%), ferritin and plasma zinc levels at 3 and 6 months of life [86]. In another study, plasma long‐chain polyunsaturated fatty acid (LCPUFA) concentrations were statistically significantly higher in HM‐fed versus standard infant formula‐fed infants at PKU diagnosis (Mean [SD, range] age at diagnosis was 20 days [3.4 days, 15–26 days] in the HM‐fed group and 19 days [4 days, 16–28 days] in the standard infant formula‐fed group) [100].

##### 3.4.1.3 Metabolic Control

Twenty‐five studies assessed metabolic control in 671 HM‐fed infants with PKU (Tables 2 and S5). In nine studies, data could only be retrieved from abstracts. Blood Phe concentrations were usually monitored daily or 2–3 times a week until levels became stable and then weekly until 6 months of life, but not in all studies [110, 116]. After 6 months, the frequency of monitoring was often reduced to biweekly or monthly measurements. Nine studies did not report the frequency or protocol for monitoring (Table S5). Blood tyrosine was obtained in only one study [86]. Acceptable ranges for target blood Phe levels varied among studies due to the differences in publication year and/or country/metabolic center.

Mean/median blood Phe concentrations, and the percentage within normal range, demonstrated that acceptable metabolic control was achieved with HMF combined with Phe‐free medical formula in almost all (99%) patients with PKU (Tables 2 and S5). Of the 12 studies comparing data on metabolic control of HM‐fed and standard infant formula‐fed infants, 10 studies showed similar metabolic control between the two groups during follow‐up. In contrast, Köse et al. [110] and O'Sullivan et al. [108] found that blood Phe concentrations were significantly lower in HM‐fed infants than standard infant formula‐fed infants and HM‐fed infants were more likely to have blood Phe concentrations in the therapeutic target range (*p* < 0.05). Most studies highlighted the advantage of the lower Phe content of HM compared with currently available standard infant formula.

No difference in blood Phe control was found by Zuvadelli et al. [112] when comparing four methods of HMF administration (i.e., premeasured HMF via bottle, on demand HMF after metabolic formula, time‐controlled HMF, or alternate feeding).

##### 3.4.1.4 Neurodevelopmental Outcomes

In total, 10 studies investigated neurodevelopment in 91 HM‐fed infants with PKU (Tables 2 and S6). It was not reported if current or historical blood Phe concentrations were considered. In five studies, data could only be retrieved from abstracts.

Two studies investigated the impact of (short) exposure to HMF (for the first 1–2 months of life) on later neurodevelopment and visual performance compared with infants who received standard infant formula feeding after delivery [97, 100]. Infants in the HM‐fed group performed better when assessed by the WISC‐R scale compared with the standard infant formula‐fed group. After adjustment for differences in social background and maternal education, there was a 12.9‐point higher IQ in the HMF group (*p* = 0.01) [97]. The second study using MDI and PDI scales to assess neural performance at 5 and 12 months found that the PDI score was significantly higher in the early HM‐fed group at 5 months, but not at 12 months. Furthermore, there were no significant differences between groups in MDI at either timepoint. A weak positive association was found between plasma LCPUFAs at diagnosis, which was higher in HM‐fed infants, and neurodevelopmental indices. They suggested that LCPUFAs supplied through HM might counteract the adverse effects of increased Phe concentrations, thereby resulting in better cognitive performance [100]. More evidence is required to support this hypothesis.

In the remaining eight studies (*n =* 66 HM‐fed infants), adequate neurological and/or psychological development was achieved in 60/66 (91%) of infants according to the authors (Tables 2 and S6). Neurodevelopment was inadequate in five infants, likely due to poor metabolic control [94, 107], and speech development at 13 years was affected in one infant due to cochlea implants and a bilingual education, but nonverbal intelligence of the patient was average [111].

##### 3.4.1.5 Immune Status

This was not evaluated in any of the studies.

##### 3.4.1.6 Psychological Outcomes in Mothers

Schulpis et al. [139, 140] investigated changes in stress in mothers of infants with PKU or HPA after diagnosis, when they were asked respectively to replace HMF completely with standard infant formula and Phe‐free medical formula (PKU groups, total *n =* 79) or could continue breastfeeding in addition to the Phe‐free medical formula (HPA group, *n =* 25). There was a significantly higher percentage of mothers with high or severe stress in the PKU group than in the HPA group (Table S7). When the stress levels were examined according to the choice of pre‐diagnosis feeding, mothers of infants with PKU who had exclusively breastfed their infants until they were asked to stop HMF experienced more stress than those who were on a combination of HMF and standard infant formula feeding at baseline [140]. Psychological support helped all mothers to feel better and to relieve stress symptoms. In three additional studies, mothers reported that being able to continue HMF was rewarding/positive for their mother–child relationship [82, 96, 102].

##### 3.4.1.7 Other Outcomes

A single study in 30 infants with PKU, breastfed for a median duration of 5 months, found no relationship between breastfeeding and feeding difficulty scores at a median age of 2 years (range 7 months to 6 years) [114].

#### 3.4.2 Amino Acid Disorders Other Than PKU

##### 3.4.2.1 Growth

In non‐PKU amino acid disorders, six studies described the growth of 12 infants fed HM and precursor‐free medical formula: *n =* 5 infants were diagnosed with MSUD, *n =* 3 infants with tyrosinemia (TYR), *n =* 3 infants with homocystinuria (HCU), and *n =* 1 infant with hypermethioninemia. Most data could only be retrieved from conference abstracts (4/6 studies). Although one infant with TYR had poor growth associated with low Phe levels at the initiation of dietary treatment (around 2 months), all infants (*n =* 12) demonstrated adequate growth at follow‐up (from 3 months up to 5 years) (Tables 3 and S4).

##### 3.4.2.2 Biochemical Assessment of Nutritional Status

This was not evaluated in any of the studies.

##### 3.4.2.3 Metabolic Control and Metabolic Decompensation

Eight studies (five conference abstracts) evaluated metabolic control in a total of 13 infants diagnosed with MSUD (*n* = 6), HCU (*n =* 4), and TYR (*n =* 3), receiving HM and precursor‐free medical formula, as well as in one infant with hypermethioninemia exclusively breastfed (Table S5). Monitoring of metabolic control varied depending on the disorder. In MSUD, plasma amino acids including plasma branched chain amino acids (BCAA) [117, 118, 120], ammonia, urinary ketones, and organic acids [118], were measured weekly to biweekly. One study did not report protocol for monitoring [119]. Plasma homocysteine, methionine [121, 122], and cysteine [122] were measured in infants with HCU, but frequency of monitoring was not reported. The single study in hypermethioninemia evaluated plasma amino acids and general biochemical monitoring every 3 months [120]. In infants with TYR, blood Phe and blood Tyr were monitored weekly [123, 124].

Metabolic control was acceptable in 5/6 infants with MSUD (Table 3) (*n* = 3 breastfed, *n* = 2 received a controlled volume of expressed HM, Table S3). In one infant, very high concentrations of BCAA were observed 1 week after feeding was changed from expressed HM with precursor‐free medical formula to on demand breastfeeding [118]. There was no observable clinical problem but the infant was hospitalized and HMF was terminated. Plasma concentrations of BCAA stabilized and on demand HMF was reintroduced at 3 months. Although the infant had satisfactory control of plasma BCAA, HMF was discontinued at 4 months of age due to the stress associated with frequent monitoring (Tables 3 and S5).

Blood tyrosine concentrations were within therapeutic target levels in all three breastfed infants with TYR [123, 124]. Only 1/3 infants had low blood Phe concentrations requiring Phe supplementation, but all biochemical markers were within therapeutic reference range at 14‐months of follow‐up (Tables 3 and S5) [124].

Blood concentrations of methionine and homocysteine were within therapeutic reference range in 3/4 breastfed infants with HCU [121, 122]. One infant struggled to take his medical formula before HMF and thus had high homocysteine concentrations because HM intake was not limited sufficiently by medical formula intake. HMF was stopped after 12 weeks (Tables 3 and S5) [121].

The infant with hypermethioninemia did not exhibit any problems or signs of poor metabolic control while exclusively breastfed (Tables 3 and S5) [120].

##### 3.4.2.4 Neurodevelopmental Outcomes

Neurodevelopmental status was reported as adequate in all four studies (two conference abstracts) where this was evaluated (7 HM‐fed infants: *n =* 5 MSUD; *n =* 1 TYR; *n =* 1 hypermethioninemia) (Tables 3 and S6).

##### 3.4.2.5 Immune Status

This was not evaluated in any of the studies.

##### 3.4.2.6 Psychological Outcomes in Mothers

This was not evaluated in any of the studies.

#### 3.4.3 Organic Acidemias

##### 3.4.3.1 Growth

Five studies (including two conference abstracts) assessed growth in a total of 22 HM‐fed infants diagnosed with OAs (*n =* 8 MMA, *n =* 5 PA, *n =* 6 GA1, *n =* 2 IVA, and *n =* 1 MA, Table S4). Most infants also received precursor‐free medical formula, sometimes with an additional protein‐free supplement. However, the infant with MA, two infants with MMA, and two infants with PA were exclusively HM‐fed (Table S3). Although the type of measurements was not reported in one conference abstract (*n =* 2 GA1), growth was described as adequate [128]. In the remaining four studies, length/height was reported as adequate in 19/20 infants, while weight was reported as adequate in 15/20 infants. At diagnosis, one infant with GA1 was underweight [118, 126], and transient poor weight gain was observed in two infants (*n =* 1 GA1 and *n =* 1 MMA) during follow‐up [120], but catch‐up was achieved in all. One infant with MMA had poor weight gain (and poor metabolic control, see below) while exclusively HM‐fed, and this did not improve with addition of precursor‐free medical formula. HMF was stopped and replaced with standard infant formula at 2 weeks of age and weight gain improved [125]. At the end of follow‐up, one infant with IVA was obese (weight > 97th centile) (Tables 4 and S4) [118, 126].

Head circumference was evaluated in only one study (*n* = 9 infants) [126]. There was a deviation from adequate head circumference in two infants with GA1, both at the time of diagnosis (> 90th centile in *n =* 1; > 97th in *n =* 1), and during follow‐up (> 97th in *n =* 2). Head growth was adequate (25–75th centiles) in the remaining seven infants during the study period (Table S4).

None of the studies compared growth between HM‐fed and standard infant formula‐fed infants.

##### 3.4.3.2 Biochemical Assessment of Nutritional Status

This was not evaluated in any of the studies.

##### 3.4.3.3 Metabolic Control and Metabolic Decompensation

Six studies (three conference abstracts) presented data on metabolic control in 35 HM‐fed infants with OAs. All studies used measurements of plasma amino acids, ammonia, urinary ketones, and organic acids to monitor metabolic control on a weekly, biweekly or monthly basis (Table S5).

Generally, infants with MMA (*n =* 13/14) and PA (*n =* 11/12) showed good metabolic control with HMF (Table 4 and S5). HMF was discontinued at 2 weeks of age for one breastfed infant with MMA after persistent metabolic acidosis and hyperammonemia (and poor weight gain, see above) [125]. HMF was also discontinued, at 4 months, for one breastfed infant with PA after two metabolic crises [118, 126]. Furthermore, in the Turkish studies [118, 126], one infant with MMA had low plasma concentrations of precursor amino acids leading to cessation of precursor‐free medical formula, while two experienced 2–3 metabolic episodes (e.g., vomiting, mild acidosis) with hospitalization. HMF was reintroduced as controlled volumes of expressed HM after management of these acute episodes, and thereafter, breastfeeding could continue in these three infants. The other infants with MMA (*n* = 10) and PA (*n =* 11) did not have any signs of metabolic instability during HMF (*n* = 13 breastfed, *n* = 8 receiving expressed HM, no direct breastfeeding, Table S3) [120, 125, 127].

HMF was usually well tolerated without any signs of poor metabolic control or metabolic crisis in patients with GA1 (*n =* 6, 3 breastfed, three breastfed after receiving controlled volumes of expressed HM first after acute management), MA (*n =* 1, exclusively breastfed), and IVA (*n =* 2, one breastfed after receiving a controlled amount of expressed HM acutely, one receiving expressed HM, no direct breastfeeding) (Table 4). One of six infants with GA1 had a metabolic decompensation at 3 months of age associated with a common viral infection [120]. Another infant had consistently low plasma lysine during the 6‐month follow‐up period (Table S5) [128]. Direct breastfeeding was not discontinued in either case.

##### 3.4.3.4 Neurodevelopmental Outcomes

Four studies (one conference abstract) investigated neurodevelopmental status in 18 HM‐fed infants diagnosed with OAs. Seven infants had an abnormal DDST II test indicating a delay in neurological development (Tables 4 and S6). Four of them (*n =* 2 MMA, *n =* 1 PA, *n =* 1 GA1) were late diagnosed (age at diagnosis: 3–30 months) [118, 120, 126], three of whom showed improvement while on HMF [118, 126]. Two infants diagnosed with PA as newborns had mild‐to‐moderate neurological impairment. Another infant diagnosed with GA1 via NBS was severely neurologically impaired due to a metabolic crisis at 3 months during a viral infection [120]. The remaining 11 infants, including 2 late diagnosed (*n =* 1 GA1 diagnosed at 2.5 months, *n =* 1 MA diagnosed at 6 months), showed adequate neurological development during HMF according to the authors (Tables 4 and S6).

##### 3.4.3.5 Immune Status

Impact of HMF on immune status was not quantitatively evaluated in the included studies. However, a Turkish research group commented that they observed a decrease in the number of infections and hospitalizations with the use of HMF in infants with OAs (no data reported) [118, 126].

##### 3.4.3.6 Psychological Outcomes in Mothers

In a single study (conference abstract), mothers of infants with MMA and PA reported an improved mental well‐being and a strengthened connection with their child when allowed to breastfeed (Table S7) [127].

#### 3.4.4 Urea Cycle Disorders

##### 3.4.4.1 Growth

Five studies (four published as conference abstracts) evaluated growth in 10 infants diagnosed with UCDs (Table S4, *n =* 1 citrullinemia, *n =* 2 OTC deficiency, *n =* 2 arginase deficiency, *n* = 4 argininosuccinate lyase [ASL] deficiency, and *n =* 1 CPS1 deficiency) who were fed HM exclusively (*n* = 5) or combined with medical formula (*n* = 5). Anthropometric findings were adequate in 9/10 infants (Tables 5 and S4). An infant with arginase deficiency had an adequate length but was underweight (weight < 3rd centile) [120].

##### 3.4.4.2 Biochemical Assessment of Nutritional Status

This was not evaluated in any of the studies.

##### 3.4.4.3 Metabolic Control and Metabolic Decompensation

Six studies (four conference abstracts) reported on metabolic control during the HMF period for 12 infants with UCDs (*n =* 5 OTC deficiency [two severe, three unreported phenotype], *n =* 2 arginase deficiency, *n* = 4 ASL deficiency, and *n =* 1 citrullinemia [all unreported phenotypes]; Table S5). Monitoring of metabolic control was similar to that in OAs and included: plasma amino acids, ammonia, and urine organic acids all measured in 1‐ to 4‐week intervals. All but one infant had acceptable metabolic control without any metabolic crisis (Tables 5 and S5) (*n* = 3 breastfed, *n* = 5 received controlled volumes of expressed HM first after management of acute episodes, then breastfed, *n* = 2 received expressed HM, no direct breastfeeding, *n* = 1 not reported, Table S3). The last infant, who had severe OTC deficiency, experienced inadequate metabolic control with hyperammonemia during an episode of respiratory infection at 1.5 months of age. On demand breastfeeding was stopped and never retried [118].

##### 3.4.4.4 Neurodevelopmental Outcomes

Four studies (three conference abstracts) reported on neurodevelopmental outcomes in seven infants with UCDs (*n =* 1 severe OTC deficiency, *n =* 2 arginase deficiency, *n* = 1 ASL deficiency, *n =* 2 CPS1 deficiency, and *n =* 1 citrullinemia). All infants had adequate neurological/psycho‐motor development (Tables 5 and S6).

##### 3.4.4.5 Immune Status

This was not evaluated in any of the studies.

##### 3.4.4.6 Psychological Outcomes in Mothers

This was not evaluated in any of the studies.

#### 3.4.5 Fatty Acid Oxidation Disorders

##### 3.4.5.1 Growth

Growth was evaluated in 11 HM‐fed infants with FAODs (Table S4, *n = 1* MCAD deficiency, *n = 3* LCHAD deficiency, *n = 1* VLCAD deficiency, *n = 1* CACT deficiency, *n = 1* CPT II deficiency, and *n = 4* LCHAD/VLCAD deficiency) from four studies (two conference abstracts). Growth was reported as adequate during the HMF period (2–12 months) for 9/11 infants (based on height and weight for *n = 2* LCHAD, *n = 1* CACT, and *n = 1* CPT II deficiency, not reported for *n* = 4 LCHAD or VLCAD deficiency) (Tables 6 and S4).

For one preterm infant with MCAD deficiency, poor weight gain was observed both in the short‐term (37 weeks gestational age) and long‐term (4 years) follow‐up, and height *z*‐score was also < −2.5 at 4 years [136]. Poor linear growth (i.e., height) was also reported in one infant with LCHAD deficiency (Tables 6 and S4) [120].

##### 3.4.5.2 Biochemical Assessment of Nutritional Status

Nutritional status was evaluated in one case report of severe CACT deficiency, who received expressed skimmed HM mixed with MCT formula and protein and carbohydrate supplements. Laboratory parameters, including vitamin D and fatty acid profiles, remained within normal reference ranges during HMF [138].

##### 3.4.5.3 Metabolic Control and Metabolic Decompensation

Data on metabolic control in FAODs was available in seven studies (two conference abstracts) (Tables 6 and S5).

For MCAD deficiency (*n =* 48 HM‐fed vs. *n =* 13 standard infant formula‐fed), the data on duration of HMF and protocol for monitoring were limited as studies were not primarily designed to investigate HMF. In two studies [134, 135], plasma levels of C8‐10 fatty acids were significantly higher in HM‐fed infants compared to standard infant formula‐fed infants, particularly during the initiation of feeding after birth [134]. No significant difference in C8 and C8/C10 ratio was observed between HMF (*n =* 11) and standard infant formula (*n =* 4) groups at follow‐up [134]. However, Ahrens‐Nicklas et al. [135] reported that 11/24 (46%) of infants on exclusive on demand HMF developed signs of metabolic decompensation, compared with none of the infants who received standard infant formula (with or without HMF). The other two patients with MCAD deficiency were asymptomatic (late diagnosis at age 2 months and 3.5 years) (Tables 6 and Table S5) [133].

The remaining three studies included infants diagnosed with severe CPT II deficiency (*n =* 1, exclusively breastfed), severe CACT deficiency (*n =* 1, receiving expressed skimmed HM with MCT and supplements), and LCHAD or VLCAD (*n =* 3 asymptomatic LCHAD exclusively breastfed or receiving expressed HM with MCT, *n* = 1 asymptomatic VLCAD exclusively breastfed, *n =* 4 either LCHAD or VLCAD [not specified which and phenotype not reported] exclusively breastfed, breastfed after low fat formula, or receiving expressed HM with low fat formula, Table S3). According to the authors, metabolic control was acceptable in all patients during HMF (2–12 months) (Table 6), with no reported metabolic decompensation, except for an initial metabolic crisis shortly after birth before diagnosis for the two infants with CACT or CPT II deficiency (Table S5) [120, 137, 138]. All infants with LCHAD or VLCAD deficiency were identified through newborn screening (Table S2).

##### 3.4.5.4 Neurodevelopmental Outcomes

Four studies (one conference abstract) evaluated neurodevelopmental status with HMF in 21 infants diagnosed with FAODs. The protocol for assessment was only provided in one study [120]. Neurological development was reported as normal in all infants, except for one of 11 HM‐fed infants with MCAD deficiency, who had neonatal hypoglycemia, and mild expressive speech delay at 2 years of age (Tables 6 and S6) [134].

##### 3.4.5.5 Immune Status

This was not evaluated in any of the studies.

##### 3.4.5.6 Psychological Outcomes in Mothers

This was not evaluated in any of the studies.

#### 3.4.6 GALE Deficiency

##### 3.4.6.1 Growth

Two infants with GALE deficiency received exclusive HMF for 3 months until it was discontinued due to insufficient milk supply [120]. During this period, both weight and length were above > 3rd centile (Table S4).

##### 3.4.6.2 Biochemical Assessment of Nutritional Status

This was not evaluated.

##### 3.4.6.3 Metabolic Control and Metabolic Decompensation

During the 3‐month HMF period, monthly measurements of galactose‐1‐phosphate level in erythrocytes were reported as satisfactory in both infants (Table S5) [120].

##### 3.4.6.4 Neurodevelopmental Outcomes

Results from the DDST II test were reported as normal in both infants during the 3‐month HMF period (Table S6) [120].

##### 3.4.6.5 Immune Status

This was not evaluated.

##### 3.4.6.6 Psychological Outcomes in Mothers

This was not evaluated.

### 3.5 Quality Appraisal and Risk of Bias Assessment

The summary of risk of bias assessment of included studies is shown in Tables S8–S10. Study quality was rated as “fair” in half of included studies (*n =* 26/52) and as “poor” in the remaining half (*n =* 26/52), indicating an overall risk of bias from medium to high. Studies conducted in non‐PKU disorders were more often rated as “poor” (*n =* 5/9 in non‐PKU AADs, *n =* 6/6 in OAs, *n =* 6/7 in UCDs, *n =* 5/7 in FAODs, and *n =* 1/1 in GALE deficiency), reflecting the paucity of data, which came from case studies (*n =* 15/21, 71%) and/or short conference abstracts (*n =* 14/22, 64%).

The lack of randomization, blinding, and power calculation as well as the allocation bias and possible confounding were the most common shortages of the controlled studies included. For the case studies, a higher risk of bias was detected due to the absence of a clear description of the case, lack of information on the outcome measures and unclear results. Uncertainties regarding the subject recruitment (e.g., homogeneity, inclusion, exclusion criteria), absence of sample size justification and/or statistical adjustments and lack of reporting on measurement of different levels of exposure (e.g., amount or duration of HMF) were the main concerns for observational and cross‐sectional studies.

## 4 Discussion

This is the first systematic review of outcomes related to HMF in infants diagnosed with any IMDs requiring dietary treatment, including, but not limited to, PKU. We collected data from 52 studies, representing 861 HM‐fed infants with an IMD. Overall, only a few IMDs would require the avoidance of HMF (e.g., clear contra‐indication for classic galactosemia). However, the strength of the evidence supporting HMF was disproportionate, with 58% of studies (86% of all HM‐fed infants) included in this review focusing on PKU. In stark contrast, for all other IMDs except MCAD deficiency, data from few HM‐fed infants were available (sometimes only one or two cases). For disorders of carbohydrate metabolism, no publication on HMF could be identified, except for GALE deficiency (galactosemia out of review scope).

In PKU, continuation of HMF in combination with a Phe‐free medical infant formula is well established and actively recommended by international guidelines (Table S11) [88, 89, 147]. Our review compiles successful outcomes across four decades, showing that HMF, combined with Phe‐free formula, has led to acceptable metabolic control (25 studies), and adequate growth (based on 15 studies) and neurodevelopment (10 studies). Comparing HM‐fed and standard infant formula‐fed infants, studies found either no differences in outcome or some suggested benefit with HMF (e.g., infants more likely to have good metabolic control [108, 110], and higher developmental scores in childhood [97, 98, 100]), which is in line with the recent scoping review by Kalvala et al. [80].

Even though supporting evidence is limited, for MSUD an evidence‐based consensus paper advocates the use of HM as the source of intact protein, with adequate growth, clinical, and biochemical monitoring, as well as adequate HM supply [148]. For TYR [149, 150] or HCU [151], guidelines only briefly mention how HMF can be possible as part of dietary management without giving an active recommendation (Table S11). In our review, based on data from 3 to 6 infants, HMF (duration one to > 18 months) appeared safe for MSUD, TYR and HCU (i.e., physical growth and neurocognitive performance within acceptable ranges according to the authors), provided that the dose of precursor‐free medical formula intake was adequate and metabolic control maintained. Medical formula plays a crucial role in attaining metabolic control whilst supporting adequate growth and development. In an infant with pyridoxine nonresponsive HCU, when medical formula intake was inadequate, plasma tHcy rose markedly, leading to discontinuation of HMF after 3 months [121]. Another infant, with MSUD, stopped HMF when he was hospitalized due to very high BCAA concentrations 1 week after feeding was changed from expressed HM with precursor‐free medical formula to on demand breastfeeding (though there was no observable clinical problem and the infant was gaining weight). Breastfeeding was reintroduced at 3 months and BCAA concentrations remained adequate. However, the stress of the frequent monitoring led the mother to stop [118]. Comparative data with standard infant formula‐fed infants were not available.

For OAs, HMF is clearly encouraged in GA1 management guidelines [152, 153], whilst guidelines for MMA and PA are somewhat more cautious, stating that HMF can be used, with careful monitoring and consideration of total protein intake (Table S11) [154, 155]. Advantages of HM in OA include its low protein and amino acid content and protection against infection [156]. Furthermore, research comparing fecal short chain fatty acid concentrations in breastfed and standard infant formula‐fed babies would suggest that HMF may reduce bacterial propionate production in the gut [157], which would be beneficial in MMA and PA. However, this has not been studied in infants with these disorders. Our search identified data from only 35 HM‐fed infants with OAs. It is worth noting that 20% of patients (*n =* 7/35) were late diagnosed (age at diagnosis: 2.5–30 months). Infants were probably well or stable, allowing HMF, explaining the somewhat longer HMF duration (up to 24 months) in this group (Table S11). Adequate growth was reported in most infants (*n* = 18/22, 82%) [118, 120, 126, 128], though there were some instances of transient poor weight gain. HMF was stopped in one infant with MMA, after 2 weeks of poor weight gain and metabolic control [125]. Overall, available data showed that approximately 86% of the infants (*n =* 30/35) had satisfactory metabolic control without any decompensation, whilst five infants (*n* = 3 MMA, *n* = 1 PA, *n* = 1 GA1) experienced 1–3 metabolic crises requiring hospitalization, mainly due to (common) infections [118, 120, 125, 126]. During these episodes of acute illness, HM was provided as expressed breast milk, allowing HMF to continue in combination with precursor‐free medical formula. On demand HMF could be resumed after the infants improved, except for two infants. One infant with MMA (mentioned above) was persistently metabolically unstable and discontinued HMF at 2 weeks of age [125]. One infant with PA had two crises 1 week apart, requiring 8 days and 2 weeks of hospitalization, respectively. This led the clinical team to recommend stopping on demand HMF [118, 126]. As infants with OAs commonly present with lethargy/impaired consciousness and poor feeding/sucking [158], on demand HMF may be impracticable, and expressed breast milk should be encouraged instead [156]. Neurological development was delayed for *n =* 7/18 infants, 4 (57%) of whom were late diagnosed, confirming the importance of early treatment initiation.

European UCD guidelines state that the main protein source for infants should be either breastfeeding or standard infant formula [159, 160]. Standard infant formulas are mainly manufactured from cow's milk, and they differ from HM in terms of protein quantity, protein fractions (casein to whey protein ratio) and amino acid profiles, although the composition of infant formulas improves continuously. Despite lower protein/nitrogen content, HM meets essential amino acids requirements [161, 162, 163]. Hence, in the protein restrictions required for UCDs, HM is advantageous as it delivers adequate essential amino acids, but with a lower total nitrogen content. Furthermore, in the latest European UCD guidelines, it was noted that exclusive demand breastfeeding is possible, but this needs close analytical/clinical monitoring. If necessary, protein intake can be lowered and controlled by giving protein‐free infant formula prior to breast feeds (Table S11) [159, 160]. In our systematic review, we included six infants who were successfully exclusively HM‐fed (*n =* 3 OTC, *n =* 2 arginase, and *n =* 1 CPS1 deficiencies). Overall, data available for UCDs and HM are limited, that is, only 1–5 infants depending on the UCD, and did not describe HMF with argininosuccinic acid synthetase deficiency. Neurocognitive development was described as adequate in all seven cases where this was assessed. Linear growth was normal but one of two infants with arginase deficiency was underweight (< 3rd centile) [120]. In general, metabolic control was reported as satisfactory with HMF except for one severe case with OTC deficiency who received HM only for 2 months (and who died later at 4 months during an episode respiratory infection leading to a metabolic crisis) [118]. In the majority of cases (*n* = 10/14), after management of the acute episode, first expressed HM was used, before transitioning to direct breastfeeding on demand (which was possible for all but one infant).

In this systematic review, most reports on FAOD and HMF were in infants diagnosed with MCAD deficiency (*n =* 48, 83%), whilst only 10 cases had other types of FAOD. Protocols for monitoring metabolic control, data about the duration and frequency of HMF, and information on fasting tolerance were lacking for the majority of studies. In MCAD deficiency, growth and neurodevelopment were rarely described. The limited data suggested that HMF was feasible, but there were more symptomatic infants in the exclusively HM‐fed group [133, 134, 135]. In addition, in at least one study, 10/34 HM‐fed infants received standard infant formula on top of HM [135]. It is important to note that there is a risk of mortality, particularly in the first 72 h after birth if HMF is not well established, because the initial supply of breast milk is low in volume and energy content, and only small volumes are consumed [164]. In the small number of infants with LCHAD or VLCAD deficiency (*n* = 8), who were all identified through newborn screening, metabolic control was considered satisfactory during HMF with no reported metabolic decompensation. However, many regions worldwide either do not screen for FAOD at all, or only few disorders are included in the testing panels. Moreover, despite improved outcomes in screened patients with FAOD compared with symptomatic presentations [165], a recent study on LCHAD deficiency showed that the rates of neonatal decompensations were still high in screened infants [166]. Symptoms in FAOD may also manifest shortly after birth, before any newborn screening results are known, as was the case for the infant with severe CACT deficiency and the infant with severe CPT II deficiency included in this review, who both presented with a metabolic crisis before diagnosis through neonatal screening [120]. Thereafter, no further decompensations were observed in both infants, and metabolic control remained satisfactory during the HMF period. However, others have reported a high mortality in the first year of life of nonscreened infants with CACT deficiency, despite adequate treatment [166, 167]. Severe, symptomatic infants with FAOD often present with poor feeding in the neonatal period [168, 169], which puts them at risk of mortality as it may affect the efficiency and sufficiency of oral intakes. Early feeding with HM needs to be well established, feed volumes need to be adequate, and any prolonged fasting should be avoided. Providing expressed HM may be preferred instead of on demand HMF. Commonly in the literature, the mode of infant feeding is not described prior to diagnosis. Hence, additional published reports of HMF in FAOD would be useful, with more detailed descriptions of feeding protocols, outcomes, and assessment methods.

Few guidelines discuss HMF in FAOD. HM is rich in lipids (3.8 g/100 mL) providing approximately half of its total calories, and HM fat content varies throughout the course of each feed, with hindmilk containing higher amounts of fat compared with foremilk [170, 171]. Long‐chain (LCT) and medium‐chain triglycerides (MCT) make up about 92% and 8% of fatty acids in HM, respectively [10]. Considering this fatty acid composition, US guidelines for VLCAD deficiency suggest that dietary management should be tailored to the severity of the disorder and clinical history of the infant [172]. Asymptomatic infants with milder phenotypes were recommended to receive HMF with or without supplementation with a special low‐LCT, high MCT medical formula, though we advise that feeding should be frequent, feed volume controlled, with fasting times minimized. For asymptomatic infants with a severe phenotype, special medical formula feeding was recommended as the primary source of nutrition. For symptomatic infants, the US guideline [172] now also suggests allowing partial HM in combination with a low LCT, high MCT medical formula to meet energy needs (Table S11). Finally, Kritzer et al. [138] showed that skimmed breast milk could successfully be combined with appropriate fat sources (including triheptanoin) to provide complete nutrition for an infant with severe CACT deficiency. The authors speculated that this regimen would also be beneficial for infants with other severe long chain FAODs.

In contrast to classic galactosemia where galactose derived from lactose in HM causes life‐threatening symptoms, in GALE deficiency, levels of Gal‐1‐phosphate are only elevated in erythrocytes. We found a single study describing HMF experience in two infants with GALE deficiency [120]. Results were promising with both infants being exclusively breastfed, albeit for a relatively short duration (1 and 4 months) due to milk shortage.

In summary for non‐PKU IMDs, more evidence is required, but the limited data suggest that HMF is possible and may be advantageous, provided it is given with disease‐specific formula supplementation where applicable, fasting is avoided when necessary, and careful monitoring is applied. HMF duration appeared to be associated with the severity of the symptoms. We aimed to collect data on a wide range of outcomes associated with HMF in infants with IMD and their mothers. However, little‐to‐no data were published for many relevant outcomes, mainly as this is not considered a priority of IMD management. There is a large body of evidence in non‐IMD infants on the protective effects of HMF against morbidity and mortality due to infectious diseases (e.g., diarrhea, lower respiratory tract, otitis media) and hospital admissions throughout the first 2 years of life (Table 1) [28, 29, 30, 31, 78], and there is no reason a priori why these benefits could not be extrapolated to IMD. In fact, these are of particular importance for infants with IMDs such as MSUD, OAs, or certain UCDs, where a common infection can have catastrophic consequences due to the risk of metabolic crisis. One research group commented that the number of infections and hospitalizations decreased with use of HMF in infants with OAs and UCDs [118, 126]. However, no data were shown to support this observation such as the frequency of infections or hospital admissions. The time of initiation and the duration of HMF will no doubt play a role in the extent to which infants will benefit. Provision of expressed HM should be considered when direct breastfeeding is challenging or temporarily risky (e.g., during episodes of metabolic decompensation). Several studies in this review have shown that infants could then transition back to direct breastfeeding on demand.

The benefits associated with HM are substantial, and we recommend its use in IMD whenever feasible, with appropriate monitoring from the metabolic team. The richness and diversity in bioactive components (i.e., live cells, immunomodulatory compounds, enzymes, hormones, growth factors, etc.) is far greater in HM than any standard infant formula [6, 21, 173]. For many IMDs such as amino acid disorders, OAs, or UCDs, unless clinical phenotype is very mild, exclusive HMF is not an option and infants require a special medical formula to safely meet their nutritional needs and ensure optimal growth and development. While these medical formulas should consider the IMD‐specific needs (e.g., omission of “offending” precursor nutrients), their composition should otherwise closely match that of HM in terms of protein, carbohydrates (e.g., lactose) and fat (e.g., long‐chain polyunsaturated fatty acids), but also bioactive components (e.g., addition of HMOs, other prebiotics, etc.) as they represent a substantial part of infant nutrition in IMD [174].

The major strength of this review is that all published clinical outcomes related to HMF in a wide group of IMD were systematically analyzed. However, the data were obtained from studies of relatively low quality with a small sample size, often without a control group and with limited information on the outcomes of interest. For non‐PKU IMDs, over 63% of studies (*n =* 14/22) were published only as conference abstracts, so they were not peer‐reviewed, and important information regarding methodology, patients' description, administration, and duration of HMF was commonly omitted. It is important to encourage clinicians to develop conference abstracts into full publications, especially for IMDs other than PKU where available data are scarce. Considering the rarity of these disorders, multicentric studies should be given a priority. This could be in the form of collections of case studies with an agreed dataset to describe HMF outcomes. Information reported should include the severity of the disorder, as well as data on actual baseline and follow‐up measurements (e.g., growth parameters, nutritional intakes, blood concentrations of relevant analytes) and the assessment methods used, including definitions of adequacy/normalcy. Articles/abstracts often mentioned qualitative statements by the authors without clear definition of a “normal” versus “abnormal” result, complicating our synthesis/interpretation of the data. For few infants, it was not clear if HMF continued beyond diagnosis (e.g., five infants in Agostoni et al.) [100], thus they were not included in the pool of HM‐fed infants. It should be noted that some studies included late diagnosed patients, who therefore may have poor clinical outcomes, but this should not be interpreted as a detrimental effect of HMF. On the other hand, the late diagnosed patients with good clinical outcome might have been breastfed without adverse event, thus giving confidence in the feasibility of HMF in the underlying disorder.

## 5 Conclusion

It has been four decades since the first administration of HM in dietary management of IMDs. Data on the short‐term effects of HMF were scarce, particularly for IMDs other than PKU. The limited evidence suggests that, with careful monitoring and disorder‐specific medical formula as appropriate, normal growth and neurological development can be achieved with HMF, with satisfactory metabolic control in most infants. We did not find any studies investigating the impact of HMF on the long‐term health of patients with IMDs or their mothers, and data comparing HMF to standard infant formula feeding in IMD were lacking, beyond several reports in PKU.

Clinicians working in the field of IMD should be encouraged to collect and publish data on short‐term and long‐term outcomes of HMF especially in IMDs other than PKU, as HM properties should benefit infants with IMDs as well. When doing so, it is important to report the severity of the disorder, sufficient details about HMF such as method of administration and duration, as well as how outcomes were evaluated. HM is a complex biofluid whose composition is still being unraveled. As special medical formulas can represent a substantial part of the nutritional intake of infants with IMDs, it is important that their composition evolves to match new HM discoveries.

Although there are many components that are associated with the feasibility and success of HMF in IMDs (e.g., metabolic stability, frequency of monitoring, experience of IMD team, mother's preference), the severity of the disorder certainly affects the outcomes. In mild forms, the diet can be less restricted in terms of protein/amino acids or fats, allowing the infant to consume greater amounts of HM without any deterioration in metabolic control. However, in severe forms, the use of HM can be problematic due to metabolic instability, severity of dietary restrictions, or the stress/interruptions related to more frequent monitoring. Support from an experienced team of clinicians, including dietitian and lactation consultant, is key to success. Factors such as the lack of knowledge and experience of IMD teams, challenges during administration, concerns regarding the safety of HMF, lack of resources for frequent and continuous monitoring or mother's choice influence the decision on the use, as well as the duration of HMF in infants with IMD. Future initiatives should address the challenges around providing HM in IMD in detail and offer practical solutions and recommendations to help initiating and continuing HMF.

## Author Contributions

Conceptualization and methodology: C.M., F.I., A.H., A.M., and D.K. Study selection, data extraction, formal analysis, risk of bias assessment and quality appraisal: F.I., A.H., and C.M. Writing – original draft: F.I. and C.M. Writing – review and editing: F.I., C.M., A.H., A.M., D.K., S.B.‐W., T.C., K.A.D., M.J.S., C.N., R.S., B.S., C.S., A.v.W., and W.W. Supervision: A.M. and D.K. Project administration: C.M. All authors approved the final version of the manuscript as submitted and agreed to be accountable for all aspects of the work. All authors confirm the absence of previous similar or simultaneous publications.

## Ethics Statement

The authors have nothing to report.

## Conflicts of Interest

Fatma Ilgaz and Alexander Höller received a small honorarium from Danone Research & Innovation as compensation for time spent screening the literature and extracting data. Camille Newby received honoraria from Danone Nutricia and Vitaflo International to attend study days and conferences. Annemiek van Wegberg has received travel grants from Danone Nutricia and Vitaflo. The UMCG and Radboudumc as employers of Annemiek van Wegberg have received a research grant from Danone Research & Innovation, and advisory board and lecture fees from Danone Research & Innovation and Vitaflo. Anita MacDonald's hospital has received research funding from Biomarin, PTC pharmaceuticals, Danone Research & Innovation, Vitaflo, Cambrooke, MetaX, Relief Therapeutics, Arla Food Ingredients. Advisory board/lecture fees have been received from: PTC pharmaceuticals, Danone Research & Innovation, Vitaflo, APR, and Arla Food Ingredients. Cyril Marsaux, Kelly A. Dingess, Bernd Stahl, Clare Szwec, Willie Woestenenk are employees of Danone Research & Innovation. The other authors declare no conflicts of interest.

## Supporting information


**Table S1.** Search strategy for the systematic review electronic database search.
**Table S2.** Main characteristics of included studies.
**Table S3.** Description of human milk feeding practices in infants with an inherited metabolic disorder.
**Table S4.** Short‐ and long‐term growth of infants with an inherited metabolic disorder who have received human milk.
**Table S5.** Metabolic control of infants with an inherited metabolic disorder during the human milk feeding period.
**Table S6.** Neurodevelopmental outcomes of infants with an inherited metabolic disorder who have received human milk.
**Table S7.** Psychological outcomes associated with human milk feeding in mothers of infants with an inherited metabolic disorder.
**Table S8.** Quality appraisal and risk of bias assessment of controlled intervention studies.
**Table S9.** Quality appraisal and risk of bias assessment of case reports and case series.
**Table S10.** Quality appraisal and risk of bias assessment of observational cohort and cross‐sectional studies.
**Table S11.** Recommendations on human milk feeding of infants with an inherited metabolic disorder, in published guidelines or consensus reports.

## Data Availability

Data are contained within the article or Supporting Information.
