# Supplementary material for: Human Milk Feeding in Inherited Metabolic Disorders: A Systematic Review of Growth, Metabolic Control, and Neurodevelopment Outcomes
Source: J Inherit Metab Dis. 2025 Feb 6;48(2):e70001. doi: 10.1002/jimd.70001 (PMC11800321; doi:10.1002/jimd.70001)
Supplement: Supplementary file 1 — Table S1. Search strategy for the systematic review electronic database search. Table S2. Main characteristics of included studies. Table S3. Description of human milk feeding practices in infants with an inherited metabolic disorder. Table S4. Short‐ and long‐term growth of infants with an inherited metabolic disorder who have received human milk. Table S5. Metabolic control of infants with an inherited metabolic disorder during the human milk feeding period. Table S6. Neurodevelopmental outcomes of infants with an inherited metabolic disorder who have received human milk. Table S7. Psychological outcomes associated with human milk feeding in mothers of infants with an inherited metabolic disorder. Table S8. Quality appraisal and risk of bias assessment of controlled intervention studies. Table S9. Quality appraisal and risk of bias assessment of case reports and case series. Table S10. Quality appraisal and risk of bias assessment of observational cohort and cross‐sectional studies. Table S11. Recommendations on human milk feeding of infants with an inherited metabolic disorder, in published guidelines or consensus reports. [file JIMD-48-0-s001.pdf]

Supplementary Tables

Supplementary Table 1. Search strategy for the systematic review electronic database search. 2

Supplementary Table 2. Main characteristics of included studies.. 4

Supplementary Table 3. Description of human milk feeding practices in infants with an inherited metabolic disorder. 11

Supplementary Table 4. Short- and long-term growth of infants with an inherited metabolic disorder who have received human milk. 23

Supplementary Table 5. Metabolic control of infants with an inherited metabolic disorder during the human milk feeding period. 30

Supplementary Table 6. Neurodevelopmental outcomes of infants with an inherited metabolic disorder who have received human milk. 42

Supplementary Table 7. Psychological outcomes associated with human milk feeding in mothers of infants with an inherited metabolic disorder.. 46

Supplementary Table 8. Quality appraisal and risk of bias assessment of controlled intervention studies. 47

Supplementary Table 9. Quality appraisal and risk of bias assessment of case reports and case series. 48

Supplementary Table 10. Quality appraisal and risk of bias assessment of observational cohort and cross-sectional studies. 49

Supplementary Table 11. Recommendations on human milk feeding of infants with an inherited metabolic disorder, in published guidelines or consensus reports... 50

Supplementary Table 1. Search strategy for the systematic review electronic database search.

SEARCH TERMS		
**#**	**IMDs**	**STRING**
S1	IEM, IMD, RMD	((inborn p/0 error*) p/1 metabolism) OR (inherited p/0 metabolic p/0 dis*) OR (rare p/0 metabolic p/0 dis*)
S2	OA	(organic p/0 acid p/0 dis*) OR (organic p/0 (aciduria* OR acidemia* OR acidaemia*))
S3	IVA	(Isovaleryl-CoA p/0 dehydrogenase p/0 def*) OR (isovaleric p/0 (aciduria OR acidemia OR acidaemia))
S4	PA	(propionyl-CoA p/0 carboxylase p/0 (def* OR (subunit p/0 (alpha OR beta) p/0 def*))) OR (propionic p/0 (aciduria OR acidemia OR acidaemia))
S5	MMA, MCE def, MCM def	(methylmalonyl-CoA p/0 (epimerase OR mutase) p/0 def*) OR (methylmalonic p/0 (aciduria OR acidemia OR acidaemia))
S6	GA1	(glutaryl-CoA p/0 dehydrogenase p/0 def*) OR (glutaric p/0 (aciduria OR acidemia OR acidaemia))
S7	MA	(malonyl-CoA p/0 decarboxylase p/0 def*) OR (malonic p/0 (aciduria OR acidemia OR acidaemia))
S8	UCD	(urea p/0 cycle p/0 (dis* OR def*))
S9	NAGSD	(N-acetylglutamate p/0 synthase p/0 def*) OR (NAGS p/0 def*)
S10	CPS1D	(Carbamoyl p/0 phosphate p/0 synthetase p/0 1 p/0 def*) OR (Carbamyl p/0 phosphate p/0 synthetase p/0 def*) OR ((CPS1 OR (CPS p/0 1)) p/0 def*)
S11	OTCD	(Ornithine p/0 transcarbamylase p/0 def*) OR (Ornithine p/0 Carbamoyltransferase p/0 def*) OR (OTC p/0 def*)
S12	ASSD	(Argininosuccinate p/0 synthetase p/0 def*) OR Citrullinuria OR citrullinemia OR citrullinaemia OR ((CTLN1 OR (CTLN p/1 1)) p/0 def*)
S13	ASLD	(Argininosuccinate p/0 lyase p/0 def*) OR (ASL p/0 def*)
S14	ARG1D	(Arginase p/0 def*) OR argininemia OR argininaemia OR ((ARG1 OR (ARG p/0 1)) p/0 def*)
S15	HHH Syndrome	(Mitochondrial p/0 ornithine p/0 transporter p/0 def*) OR (ornithine p/0 (translocase OR carrier)) OR (hyperornithinemia p/0 hyperammonemia p/0 homocitrullinuria p/0 syndrome) OR (HHH p/0 syndrome)
S16	Citrin D	(Citrin p/0 def*) OR ((CTLN2 OR (CTLN p/1 2)) p/0 def*)
S17	Carbonic anhydrase VA deficiency	(Carbonic p/0 anhydrase p/0 VA p/0 def*)
S18	Glutamate dehydrogenase superactivity	(Glutamate p/0 dehydrogenase p/0 superactivity)
S19	AA disorders	(Amino p/0 acid p/0 (dis* OR def*)) OR Aminoacidopath*
S20	MSUD	(Branched-chain p/0 ketoacid p/0 dehydrogenase p/0 (def* OR (E1 p/0 (alpha OR beta) p/0 def*))) OR (maple p/0 syrup p/0 urine p/0 dis*) OR MSUD
S21	PKU	(Phenylalanine p/0 hydroxylase p/0 def*) OR phenylketonuria OR phenylalaninemia OR phenylalaninaemia OR hyperphenylalaninemia OR hyperphenylalaninaemia OR PKU
S22	TYR1/TYR2	(Tyrosine p/0 aminotransferase p/0 def*) OR (Fumarylacetoacetase p/0 def*) OR tyrosinemia OR tyrosinaemia
S23	CBS def	(Cystathionine p/0 beta-synthase p/0 def*) OR homocystinuria OR (CBS p/0 def*)
S24	FOAD	(Fatty p/0 acid p/0 oxidation p/0 dis*) OR FAOD
S25	VLACCD	(Very p/0 long-chain p/0 acyl-CoA p/0 dehydrogenase p/0 def*) OR (VLCAD p/0 def*)
S26	MTP def	(Trifunctional p/0 protein p/0 subunit p/0 (alpha OR beta) p/0 def*) OR (trifunctional p/0 protein p/0 def*) OR (MTP p/0 def*)
S27	LCHADD	(long-chain p/0 3-hydroxyacyl-CoA p/0 dehydrogenase p/0 def*) OR (3-hydroxyacyl-CoA p/0 dehydrogenase p/0 long p/0 chain p/0 def*) OR (long-chain p/0 3-OH p/0 acyl-CoA p/0 dehydrogenase p/0 def*) OR (LCHAD p/0 def*)
S28	MCADD	(medium-chain p/0 acyl-CoA p/0 dehydrogenase p/0 def*) OR (MCAD p/0 def*)
S29	GSD	(glycogen p/0 storage p/0 dis*)
**#**	**Human milk**	**STRING**
S30		breastfe* OR (breast p/0 fe*) OR ((human OR breast OR maternal) p/0 milk) OR (mother* p/1 milk) OR breastmilk

Final search string	
Ti,ab(((inborn p/0 error*) p/1 metabolism) OR (inherited p/0 metabolic p/0 dis*) OR (rare p/0 metabolic p/0 dis*) OR (organic p/0 acid p/0 dis*) OR (organic p/0 (aciduria* OR acidemia* OR acidaemia*)) OR (Isovaleryl-CoA p/0 dehydrogenase p/0 def*) OR (isovaleric p/0 (aciduria OR acidemia OR acidaemia)) OR (propionyl-CoA p/0 carboxylase p/0 (def* OR (subunit p/0 (alpha OR beta) p/0 def*))) OR (propionic p/0 (aciduria OR acidemia OR acidaemia)) OR (methylmalonyl-CoA p/0 (epimerase OR mutase) p/0 def*) OR (methylmalonic p/0 (aciduria OR acidemia OR acidaemia)) OR (glutaryl-CoA p/0 dehydrogenase p/0 def*) OR (glutaric p/0 (aciduria OR acidemia OR acidaemia)) OR (malonyl-CoA p/0 decarboxylase p/0 def*) OR (malonic p/0 (aciduria OR acidemia OR acidaemia)) OR (urea p/0 cycle p/0 (dis* OR def*)) OR (N-acetylglutamate p/0 synthase p/0 def*) OR (NAGS p/0 def*) OR (Carbamoyl p/0 phosphate p/0 synthetase p/0 1 p/0 def*) OR (Carbamyl p/0 phosphate p/0 synthetase p/0 def*) OR ((CPS1 OR (CPS p/0 1)) p/0 def*) OR (Ornithine p/0 transcarbamylase p/0 def*) OR (Ornithine p/0 Carbamoyltransferase p/0 def*) OR (OTC p/0 def*) OR (Argininosuccinate p/0 synthetase p/0 def*) OR Citrullinuria OR citrullinemia OR citrullinaemia OR ((CTLN1 OR (CTLN p/1 1)) p/0 def*) OR (Argininosuccinate p/0 lyase p/0 def*) OR (ASL p/0 def*) OR (Arginase p/0 def*) OR argininemia OR argininaemia OR ((ARG1 OR (ARG p/0 1)) p/0 def*) OR (Mitochondrial p/0 ornithine p/0 transporter p/0 def*) OR (ornithine p/0 (translocase OR carrier)) OR (hyperornithinemia p/0 hyperammonemia p/0 homocitrullinuria p/0 syndrome) OR (HHH p/0 syndrome) OR (Citrin p/0 def*) OR ((CTLN2 OR (CTLN p/1 2)) p/0 def*) OR (Carbonic p/0 anhydrase p/0 VA p/0 def*) OR (Glutamate p/0 dehydrogenase p/0 superactivity) OR (Amino p/0 acid p/0 (dis* OR def*)) OR Aminoacidopath* OR (Branched-chain p/0 ketoacid p/0 dehydrogenase p/0 (def* OR (E1 p/0 (alpha OR beta) p/0 def*))) OR (maple p/0 syrup p/0 urine p/0 dis*) OR MSUD OR (Phenylalanine p/0 hydroxylase p/0 def*) OR phenylketonuria OR phenylalaninemia OR phenylalaninaemia OR hyperphenylalaninemia OR hyperphenylalaninaemia OR PKU OR (Tyrosine p/0 aminotransferase p/0 def*) OR (Fumarylacetoacetase p/0 def*) OR tyrosinemia OR tyrosinaemia OR (Cystathionine p/0 beta-synthase p/0 def*) OR homocystinuria OR (CBS p/0 def*) OR (Fatty p/0 acid p/0 oxidation p/0 dis*) OR FAOD OR (Very p/0 long-chain p/0 acyl-CoA p/0 dehydrogenase p/0 def*) OR (VLCAD p/0 def*) OR (Trifunctional p/0 protein p/0 subunit p/0 (alpha OR beta) p/0 def*) OR (trifunctional p/0 protein p/0 def*) OR (MTP p/0 def*) OR (long-chain p/0 3-hydroxyacyl-CoA p/0 dehydrogenase p/0 def*) OR (3-hydroxyacyl-CoA p/0 dehydrogenase p/0 long p/0 chain p/0 def*) OR (long-chain p/0 3-OH p/0 acyl-CoA p/0 dehydrogenase p/0 def*) OR (LCHAD p/0 def*) OR (medium-chain p/0 acyl-CoA p/0 dehydrogenase p/0 def*) OR (MCAD p/0 def*) OR (glycogen p/0 storage p/0 dis*)) AND Ti,ab(breastfe* OR (breast p/0 fe*) OR ((human OR breast OR maternal) p/0 milk) OR (mother* p/1 milk) OR breastmilk)	
Databases	All 97 databases available on ProQuest Dialog
Language	English
Publication date	No restrictions (last search carried out on 24-07-2024)
# of results	490

Supplementary Table 2. Main characteristics of included studies.

Group of disorders	Condition	N of studies and infants	References	Country (N of centres)	Study design	Feeding type after diagnosis	Sample size	Gender M:F	Time of diagnosis [a]	Phenotype	Clinical presentation at diagnosis
**Amino acid disorders (AADs)**	*Phenylketonuria (PKU)*	30 studies<br>HMF: 738<br>Std IF: 679<br>Total: 1417	Francis 1981[34]	UK (n=1)	Case series	HMF:	6	n/r	n/r	n/r	n/r
			McCabe 1989[38]	USA (n=1)	Non-randomized controlled study, prospective	HMF:	18	n/r	Early (NBS)	n/r	Asymptomatic
						Std IF:	10				
			Greve 1994[37]	USA (n=1)	Non-randomized controlled study, prospective	HMF:	9	n/r	Early (NBS)	Classic [b]	Asymptomatic
						Std IF:	4				
			Miller 1994[48]	USA (n=1)	Case report	HMF:	1	n/r	Early (NBS)	Classic	Asymptomatic
			Riva 1996[64]	Italy (n=1)	Observational cohort study, retrospective	HMF:	5 [c]	8:5	Early (neonatal)	Classic	n/r
						Std IF:	21	5:8			
			Duncan 1997[36]	USA (n=1)	Case report	HMF:	1	0:1	Early (NBS)	Classic	Asymptomatic
			Motzfeldt 1999[39]	Norway (n=1)	Observational cohort study, retrospective	HMF:	74	n/r	Early (NBS)	Classic	Asymptomatic
						Std IF:	9				
			Davidson 2000[86]	Canada (n=1)	Observational cohort study, retrospective	HMF:	33	n/r	n/r	n/r	n/r
						Std IF:	19				
			Francis 2000[163]	Australia (n=1)	Case report	HMF:	1	0:1	Early (NBS)	Mild [b]	Asymptomatic
			Agostoni 2003[59]	Italy (n=1)	Non-randomized controlled study, prospective	HMF:	7 [c]	7:5	Early (NBS)	Classic [b]	Asymptomatic
						Std IF:	13	4:4			
			Cornejo 2003[47]	Chile (n=1)	Observational, cross-sectional study	HMF:	19	9:10	Early (neonatal)	n/r	Asymptomatic
			van Rijn 2003[164]	Netherlands (n=1)	Non-randomized controlled study, retrospective	HMF:	9	2:7	Early (NBS)	Mixed [b]	Asymptomatic
						Std IF:	9	5:4			
			Nielsen 2005[49]	Denmark (n=1)	Case series	HMF:	6 [d]	n/r	n/r	Classic	n/r
			Kanufre 2007[46]	Brazil (n=1)	Non-randomized controlled study, prospective	HMF:	35	21:14	Early (NBS)	Mixed [b]	Asymptomatic
						Std IF:	35	21:14			

Group of disorders	Condition	N of studies and infants	References	Country (N of centres)	Study design	Feeding type after diagnosis	Sample size	Gender M:F	Time of diagnosis [a]	Phenotype	Clinical presentation at diagnosis
			Sweeney 2009[165]	Australia (n=1)	Observational cohort study, retrospective	HMF:	22	n/r	Early (NBS)	n/r	Asymptomatic
						Std IF:	5				
			Santos 2011[166]	Brazil (n=1)	Observational cohort study, retrospective	HMF:	39	22:17	Early (≤40 days)	n/r	n/r
						Std IF:	39	22:17			
			Sweeney 2011[167]	Australia (n=1)	Case report	HMF:	1	1:0	Early (NBS)	Mild	Asymptomatic
			Banta-Wright 2012[168]	USA (n=27)	Observational cohort study, retrospective	HMF:	75	39:36	Early (NBS)	Mixed [b]	Asymptomatic
						Std IF:	22	9:13			
			Lamônica 2012[63]	Brazil (n=1)	Observational cohort study, prospective	HMF:	10	6:4	Early (NBS)	n=5 Classic n=5 Mild-moderate [b]	Asymptomatic
			O'Sullivan 2013[68]	Ireland (n/r)	Observational cohort study, retrospective	HMF:	45	n/r	n/r	Classic and moderate	n/r
						Std IF:	128				
			Sweeney 2016[169]	Australia (n=1)	Case report	HMF:	1	0:1	Early (NBS)	Classic	Asymptomatic
			Köse 2018[65]	Turkey (n=1)	Observational cohort study, retrospective	HMF:	25	11:14	Early (NBS)	Classic	Asymptomatic
						Std IF:	16	9:7			
			Schulpis 2019[51]	Greece (n=1)	Observational cross-sectional study	HMF:	0 [c]	n/r	Early (NBS)	n/r	Asymptomatic
						Std IF:	54				
			Weiss 2020[69]	Germany (n=1)	Case series	HMF:	3	3:0	Early (NBS)	n=2 Classic [b], n=1 BH4 responsive	Asymptomatic
			Schulpis 2021[50]	Greece (n=1)	Observational cross-sectional study	HMF:	25	n/r	Early (NBS)	HPA	Asymptomatic
						Std IF:	25 [c]	n/r		Classic	
			Zuvadelli 2022[66]	Italy (n=1)	Observational cohort study, retrospective	HMF:	28	n/r	Early (NBS)	n=18 Classic n=15 Mild n=9 HPA	Asymptomatic
						Std IF:	14	n/r			
			Rice 2023[170]	Ireland (n=1)	Observational cohort study, retrospective	HMF:	16	24:15	Early (NBS)	n/r	Asymptomatic
						Std IF:	23				
			Rocha 2023[70]	Brazil (n=1)	Non-randomized controlled study, retrospective	HMF:	30	21:19	Early (neonatal)	n=26 Classic n=13 Mild n=1 n/r	n/r
						Std IF:	10				

Group of disorders	Condition	N of studies and infants	References	Country (N of centres)	Study design	Feeding type after diagnosis	Sample size	Gender M:F	Time of diagnosis [a]	Phenotype	Clinical presentation at diagnosis
			Guillén-López 2024[171]	Mexico (n=1)	Observational cohort study, retrospective	HMF:	181	n/r	n/r	n/r	n/r
						Std IF:	202				
			Mohammad zadeh 2024[67]	Iran (n=1)	Observational cohort study, retrospective	HMF:	13	5:8	Early (NBS)	Classic	Asymptomatic
						Std IF:	21	10:11			
Other Amino Acid Disorders	*Maple Syrup Urine Disease (MSUD)*	4 studies HMF: 6	Touati 2001[71]	France (n=1)	Case series	HMF:	3	n/r	Early (neonatal/ prenatal)	n/r	n=1 Severe coma, n=1 n/a (prenatal) n=1 Asymptomatic
			Huner 2005[56]	Turkey (n=1)	Observational cohort study, prospective	HMF:	1	1:0	Early (10 days)	n/r	Metabolic crisis
			Ross 2016[72]	India (n=1)	Case report	HMF:	1	n/r	Early (prenatal)	n/r	Prenatal diagnosis
			Pichler 2017[57]	Austria (n=1)	Observational cohort study, retrospective	HMF:	1	n/r	Early (NBS)	n/r	Asymptomatic
	Homocystinuria (HCU)	2 studies HMF: 4	Dixon 2014[74]	UK (n=1)	Case series	HMF:	3	n/r	Early (NBS)	Pyridoxine non-responsive	Asymptomatic
			Aktuğlu 2015[73]	Turkey (n=1)	Case report	HMF:	1	0:1	Early (n/r)	n/r	Asymptomatic
	Hypermethioninemia	1 study HMF: 1	Pichler 2017[57]	Austria (n=1)	Observational cohort study, retrospective	HMF:	1	n/r	Early (NBS)	n/r	Asymptomatic
	Tyrosinemia (TYR)	2 studies HMF: 3	Daly 2005[75]	UK (n=1)	Case series	HMF:	2	2:0	Early (NBS)	Type I	Asymptomatic
			Silva 2022[76]	Portugal (n=1)	Case report	HMF:	1	1:0	Early (NBS)	Type II	Asymptomatic
Organic acidemias (OAs)	*Methylmalonic acidemia (MMA)*	5 studies HMF: 14 Std IF: 5 Total: 19	Dixon 2000[78]	UK (n=2)	Case series	HMF:	2	1:1	Early (prenatal & 3 weeks)	n=1 Mutase[0] n=1 n/r	n=1 Metabolic crisis
			Huner 2005[56]; Gökçay 2006[55]	Turkey (n=1)	Observational cohort study, prospective	HMF:	4	2:2	n=2 Early (1 & 3 days) n=2 Late (3 & 6 months)	n=2 Mutase-negative n=2 n/r	n=2 Metabolic crisis n=1 Asymptomatic n=1 Failure to thrive & psychomotor retardation

Group of disorders	Condition	N of studies and infants	References	Country (N of centres)	Study design	Feeding type after diagnosis	Sample size	Gender M:F	Time of diagnosis [a]	Phenotype	Clinical presentation at diagnosis
			Pichler 2017[57]	Austria (n=1)	Observational cohort study, retrospective	HMF:	2	n/r	n=1 Early (NBS)<br>n=1 Late (5 months)	n/r	n=1 Asymptomatic<br>n=1 Failure to thrive
			Starin 2024[52]	USA (n=1)	Observational cohort study, retrospective	HMF:	6	2:4	Early (1-4 days or NBS)	Moderate to severe [e]	n=2 Asymptomatic<br>n=4 Symptomatic
						Std IF:	5	4:1	Early (prenatal, 3, 12 days, or NBS)	Moderate to severe [e]	n=2 Asymptomatic<br>n=3 Symptomatic
	Propionic acidemia (PA)	5 studies<br><br>HMF: 12<br>Std IF: 8<br>Total: 20	Dixon 2000[78]	UK (n=1)	Case report	HMF:	1	1:0	Early (2 days)	n/r	Asymptomatic [f]
			Huner 2005[56]; Gökçay 2006[55]	Turkey (n=1)	Observational cohort study, prospective	HMF:	1	1:0	Early (6 days)	n/r	Metabolic crisis
			Pichler 2017[57]	Austria (n=1)	Observational cohort study, retrospective	HMF:	3	n/r	n=2 Early (NBS & newborn)<br>n=1 Late (2.5 years)	n/r	n=1 Asymptomatic<br>n=2 Metabolic crisis
			Starin 2024[52]	USA (n=1)	Observational cohort study, retrospective	HMF:	7	5:2	Early (2 & 3 days or NBS)	Severe [e]	n=4 Asymptomatic<br>n=3 Symptomatic
						Std IF:	8	3:5	n=6 Early (17 days or NBS)<br>n=1 Late (12 months)<br>n=1 n/r	Severe [e]	n=4 Asymptomatic<br>n=4 Symptomatic
	Glutaric acidemia Type 1 (GA1)	4 studies<br>HMF: 6	Huner 2005[56]; Gökçay 2006[55]	Turkey (n=1)	Observational cohort study, prospective	HMF:	2	1:1	Late (2.5 & 6 months)	Type I	n=1 Metabolic crisis<br>n=1 Macrocephaly
			Fitzachary 2015[77]	UK (n=1)	Case series	HMF:	2	n/r	Early (NBS)	Type I	Asymptomatic
			Pichler 2017[57]	Austria (n=1)	Observational cohort study, retrospective	HMF:	2	n/r	Early (NBS)	Type I	n/r
	Isovaleric acidemia (IVA)	2 studies<br>HMF: 2	Huner 2005[56]; Gökçay 2006[55]	Turkey (n=1)	Observational cohort study, prospective	HMF:	2	0:2	Early (8 days)	n/r	Metabolic crisis
	Malonic acidemia (MA)	1 study<br>HMF: 1	Pichler 2017[57]	Austria (n=1)	Observational cohort study, retrospective	HMF:	1	n/r	Late (6 months)	n/r	Dilatative cardiomyopathy

Group of disorders	Condition	N of studies and infants	References	Country (N of centres)	Study design	Feeding type after diagnosis	Sample size	Gender M:F	Time of diagnosis [a]	Phenotype	Clinical presentation at diagnosis
**Urea Cycle Disorders (UCDs)**	*Citrullinemia*	1 study<br>HMF: 1	Kamper 2001[172]	Austria (n=1)	Case report	HMF:	1	1:0	Early (3 days)	n/r	Metabolic crisis and neurologic deterioration
	Ornithine transcarbamylase (OTC) deficiency	4 studies<br>HMF: 5	Rawlinson 2000[173]	UK (n=1)	Case series	HMF:	2	1:1	Early (prenatal & 2 days)	n/r	n=1 Asymptomatic<br>n=1 Metabolic crisis
			Huner 2005[56]	Turkey (n=1)	Observational cohort study, prospective	HMF:	1	1:0	Early (5 days)	Severe	Severe metabolic crisis
			Pichler 2017[57]	Austria (n=1)	Observational cohort study, retrospective	HMF:	1	n/r	Late (18 months)	Severe	Metabolic crisis
			Burfield 2024[174]	USA (n=1)	Observational cohort study, prospective	HMF:	1	0:1	Early (3 days)	n/r	Neonatal hyperammonemia
	Arginase deficiency	1 study<br>HMF: 2	Pichler 2017[57]	Austria (n=1)	Observational cohort study, retrospective	HMF:	2	n/r	Early (1 month)	n/r	Metabolic crisis
	Argininosuccinate lyase (ASL) deficiency	2 studies<br>HMF: 4	Dixon 2000[78]	UK (n=2)	Case series	HMF:	2	2:0	Early (1-3 days)	n/r	n=1 Metabolic crisis<br>n=1 Asymptomatic [f]
			Burfield 2024[174]	USA (n=1)	Observational cohort study, prospective	HMF:	2	0:2	Early (NBS & 2 days)	n/r	n=1 Asymptomatic<br>n=1 Neonatal hyperammonemia
	Carbamoyl phosphate synthetase 1 (CPS 1) deficiency	1 study<br>HMF:2	Bzduch 2019[175]	Slovakia (n=1)	Case series	HMF:	2	1:1	Early (neonatal)	Mild	n/r
**Fatty Acid Oxidation Disorders (FAODs)**	*Medium-chain acyl-CoA dehydrogenase (MCAD) deficiency*	4 studies<br><br>HMF: 48<br>Std IF: 13<br>Total: 61	Roe 1986[83]	USA (n=1)	Case series	HMF:	2	1:1	Late (2 months & 3.5 years)	n/r	Asymptomatic
			Hsu 2008[82]	USA (n/r)	Non-randomized controlled study, retrospective	HMF:<br>Std IF:	11 [g]<br>4	n/r	Early (NBS)	985A□G homozygote (Severe)	n=2 Symptomatic (HM-fed)<br>n=13 Asymptomatic
				USA (n=1)		HMF:	34 [h]	n/r	Early (NBS)		n=11 Metabolic crisis<br>n=23 Asymptomatic

Group of disorders	Condition	N of studies and infants	References	Country (N of centres)	Study design	Feeding type after diagnosis	Sample size	Gender M:F	Time of diagnosis [a]	Phenotype	Clinical presentation at diagnosis
			Ahrens-Nicklas 2016[81]		Non-randomized controlled study, retrospective	Std IF:	9			53%: 985A□G homozygote (Severe)	Asymptomatic
			Petropoulou 2017[79]	UK (n=1)	Case report	HMF:	1	1:0	Early (5 days)	n/r	Asymptomatic
	Long-chain hydroxyacyl-coA dehydrogenase (LCHAD) deficiency	1 study HMF: 3	Pichler 2017[57]	Austria (n=1)	Observational cohort study, retrospective	HMF:	3	n/r	Early (NBS)	n/r	Asymptomatic
	Very long-chain acyl-CoA dehydrogenase (VLCAD) deficiency	1 study HMF: 1	Pichler 2017[57]	Austria (n=1)	Observational cohort study, retrospective	HMF:	1	n/r	Early (NBS)	n/r	Asymptomatic
	Mixed (LCHAD or VLCAD)	1 study HMF: 4	Hussa 2006[84]	USA (n=1)	Case series	HMF:	4	n/r	Early (NBS)	n/r	n/r
	Carnitine-acylcarnitine translocase (CACT) deficiency	1 study HMF: 1	Kritzer 2020[80]	USA (n=1)	Case report	HMF:	1	0:1	Early (NBS)	Severe	Metabolic crisis
	Carnitine palmitoyltransferase II (CPT II) deficiency	1 study HMF: 1	Pichler 2017[57]	Austria (n=1)	Observational cohort study, retrospective	HMF:	1	n/r	Early (1 week)	Severe	Metabolic crisis
**Other**	*Galactose epimerase (GALE) deficiency*	1 study HMF: 2	Pichler 2017[57]	Austria (n=1)	Observational cohort study, retrospective	HMF:	2	n/r	Early (NBS)	Mild	Asymptomatic

Abbreviations: Dx, diagnosis; HMF, human milk feeding; M:F, male:female; N, number; NBS, newborn screening; n/a, not applicable; n/r, not reported; Std IF, standard (commercial) infant formula feeding.

[a] Diagnosis ≤1 month or >1 month of age was defined as 'early' or 'late', respectively. For Santos 2011, diagnosis was within 40 days of birth and kept summarized as 'Early' in the table.

[b] Phenotype descriptions were made based on the diagnostic (mean ± SD or median [range]) blood Phe levels or mutation analysis; Greeve, 1994 - HM-fed: 1216.85 ± 147.7 µmol/L, Std IF: 1584.33 ± 145.3 µmol/L; Francis, 2000 - Phe at 2 & 9 d were 633 & 1049 µmol/L, respectively; Agostoni, 2003 - HM-fed: 1398 ± 444 µmol/L, Std IF: 1260 ± 588 µmol/L; van Rijn, 2003 - HM-fed: 1600 [390-2200] µmol/L, Std IF: 780 [200-4150] µmol/L; Kanufre, 2007 - HM-fed: 1134 [318 - 2718] µmol/L, Std IF: 1128 [360 - 2880] µmol/L; Banta-Wright, 2012 - HM-fed (mean, range): 1126.8 [240–3,534] µmol/L, Std IF (mean, range): 1339.8 (480–2,802) µmol/L; Weiss, 2020 - Infant #1: c.165 delT / c.284_286 delTCA, Infant #2: c.1222 C>T / c.1222 C>T (classic), Infant #3: c.1066-11 G>A / c.1222 C>T (classic).

[c] Riva, 1996: 8/13 infants with PKU stopped HMF at diagnosis and switched to Std IF, while 5/13 continued HMF for <2 weeks after diagnosis. Agostoni, 2003: Of 12 infants exclusively breastfed until diagnosis, at least 7 continued to be HM-fed for a short duration; unclear for the other 5 if HMF was stopped at diagnosis or shortly after. Schulpis, 2019 and 2021: Infants with PKU were initially HM-fed, either partially or exclusively, before diagnosis. All infants switched to exclusive Std IF feeding after diagnosis. For

[d] Nielsen, 2005: In 3 infants, HM was temporarily discontinued until blood Phe level was <900 µmol/L, whereas in the remaining 3 infants, the clinicians allowed HMF without any breaks although the Phe level was >900µmol/L.

[e] Starin, 2024: Likely phenotype predicted based on mutations.

[f] Dixon, 2000: The infant with PA and one infant with ASL deficiency were asymptomatic, but they were treated from birth, before diagnosis, because of a previous sibling with the same condition.

[g] Hsu, 2008: In addition to the 15 infants, initial feeding type was not known in n=5 infants (HMF plus standard infant formula or not specified).

[h] Ahrens-Nicklas, 2016: Type of feeding was unknown in n=3 infants.

Supplementary Table 3. Description of human milk feeding practices in infants with an inherited metabolic disorder.

Group of disorders	Condition	References	N of HM-fed infants		Age at HMF initiation<br>Mean ± SD;<br>[Median, range]	Duration of HMF<br>Mean ± SD;<br>[Median, range]	Exclusive vs. partial HMF after diagnosis	HMF mode of administration after diagnosis	Protocol
			Before diagnosis:	After diagnosis:					
**Amino Acid Disorders (AADs)**	*Phenylketonuria (PKU)*	Francis 1981	6	6	At birth	n/r	HMF + PS	PS before on demand breastfeeding	Breastfeeding supplemented with a low Phe PS (15% dilution, five feeds/day). Appropriate vitamin supplements were given daily. Water and fruit juice were discouraged unless the baby was unwell. Small quantities of solids were started from 4 months.
		McCabe 1989	18	18	At birth	8.9±7.3 months<br>[1.5-25.5]<br>n=16<br><br>*≥6 months*<br>n=2	HMF + PS	Alternating breastfeeding and PS	Low Phe PS and supplemental breastfeeding. Mothers were instructed to separate breast and bottle feedings because of authors' concern that the low Phe PS might decrease the bioavailability of iron from breast milk; an iron supplement was added if haematocrit was approaching 30%.
		Greve 1994	9	9	At birth	3 months<br>[1.3-5.2]<br>n=5<br><br>*≥6 months*<br>n=4	HMF + PS	PS before on demand breastfeeding	Phe-free PS combined with breastfeeding; dietary adjustments were made by adding or subtracting Phe-free PS in response to dietary history and the fluctuations in blood Phe levels determined 2x weekly.
		Miller 1994	1	1	At birth	*≥3 months*	HMF + PS	Alternating breastfeeding and PS	Breastfeeding until diagnosis (day 10). Then, Phe-free PS for 3 days until acceptable Phe levels were achieved. At 14 d of age, the infant was fed with 3x breastfeeds/day and 3x Phe-free PS/day.
		Riva 1996	13	5	At birth	*>0.9 months*<br>[0.7-1.3]	HMF + PS	Controlled expressed HM before or after PS	Infants were exclusively breast-fed at delivery until diagnosis (for ~26 days); partial breastfeeding was started upon diagnosis in 5 of 13 (38.5%) infants; they received around l00 ml/day of expressed HM + modified diet/PS for <2 weeks; the other n=8 (61.5%) stopped breastfeeding upon diagnosis.
		Duncan 1997	1	1	At birth	11 months	HMF + PS	Controlled expressed HM before PS; then 'Time-controlled' breastfeeding before PS	Phe-free PS for 3d after diagnosis until blood Phe levels reached target range. At day 20, Phe was introduced with ~240 ml expressed HM (=29 mg/kg/d Phe) with ad lib Phe-free PS intake. At 7 weeks, family opted for transition to a breastfeeding timed system (90 min. breastfeeding/24h) with ad lib PS. At 5 months; complementary feeding was started and HM was reduced until complete weaning at 11 months.
		Motzfeldt 1999	n/r	74	At birth	7 months<br>[1-16]	HMF + PS	PS before on demand breastfeeding	Initially Phe-free PS was given as 60-70% of fluid intake divided into 6-9 feeds and was reduced 5-20 ml/feed every other day; breastfeeding on demand was given after each PS feed; HMF was postponed for 1-3 d only if serum Phe >1200 µmol/L;<br>Infants were discharged from the hospital after 7–10 d:

Group of disorders	Condition	References	N of HM-fed infants Before diagnosis:	After diagnosis:	Age at HMF initiation Mean ± SD; [Median, range]	Duration of HMF Mean ± SD; [Median, range]	Exclusive vs. partial HMF after diagnosis	HMF mode of administration after diagnosis	Protocol
									intake on average 60% HMF, and 40% PS; solid food introduced by 4-6 months of age, given after PS and HMF.
		Davidson 2000	n/r	33	n/r	*≤15 months*	HMF + PS	PS before on demand breastfeeding	Infants given a specified amount of low Phe PS before being allowed to breastfeed ad lib. Amount of PS adjusted according to blood Phe levels.
		Francis 2000	1	1	3 days	*≥5 months*	HMF + PS	PS before on demand breastfeeding	First 2 days, the infant only received Phe-free PS (150 mL/kg/day) until metabolic control was achieved. Then, Phe-free PS was reduced to 30-50 mL/kg and given before each breast feed.
		Agostoni 2003	12	n/r<br>≥7	At birth	*≤1 month; n=5*<br>*≤2 months; n=7*	HMF + PS	n/r	In the HMF group. Infants were exclusively breastfed at least until diagnosis (15-26 days). After diagnosis, infants were placed on a low Phe diet (e.g., Phe-free PS and vegetables with a low Phe content) to maintain plasma Phe levels <360 µmol/L. Five mothers stopped breastfeeding within the first month of life (unclear how many continued after diagnosis), and 7 mothers within the second month (i.e., they continued at least a little after diagnosis).
		Cornejo 2003	n/r	19	Within a week after diagnosis	*≥6 months; n=14*<br>*<6 months; n=5*	HMF + PS<br>(+/- Std IF)	PS before on demand breastfeeding	Total volume of liquid to be ingested was estimated as 150 ml/kg/day. After diagnosis, breastfeeding was suspended for 1 week. Liquid intake was 100% Phe-free PS, while caloric intake was completed with maltodextrin and vegetable oil, preferably soybean oil. On day 5 of treatment, blood Phe levels were evaluated. If they decreased to <480-600 µmol/L, Phe-free PS was reduced by 50%, and volume was completed with breastfeeding after each bottle feed (every 3h). Mothers recorded volume of PS, duration and frequency of breastfeeding. If blood Phe level was 120-360 µmol/L, prescription was maintained. If <120 µmol/L, PS was reduced by 25%, indirectly increasing the volume of breast milk. If blood Phe was 360-600 mmol/L, volume of PS was increased by 25%, and breast milk intake was indirectly decreased. If Phe level >600 µmol/L, Phe-free PS was increased by 50% of the total prescribed liquids (150 ml/kg/day).<br>For 5 infants, breast milk supply was insufficient: std IF was introduced starting at 2 months and complementary foods (at 4 months of age. The remaining 14 infants maintained breastfeeding as the only source of Phe.

Group of disorders	Condition	References	N of HM-fed infants: Before diagnosis:	After diagnosis:	Age at HMF initiation Mean ± SD; [Median, range]	Duration of HMF Mean ± SD; [Median, range]	Exclusive vs. partial HMF after diagnosis	HMF mode of administration after diagnosis	Protocol
		van Rijn 2003	9	9	At birth	[2.5 months, 1.8-8.3]	HMF + PS	Alternating breastfeeding and PS	The number of breastfeeds was adapted to the plasma Phe concentrations. At each feeding, either PS or breastfeeding, the child was allowed to drink until satiety.
		Nielsen 2005	6	6 [b]	n/r	n/r	HMF + PS	PS before on demand breastfeeding	Phe-free PS given 7-8x daily, volume set to approximately 1/6 of infant's weight. When ~80% of PS has been drunk, the mother could breastfeed ad lib.
		Kanufre 2007	35	35	At birth	7.5±4.0 months [6.6, 1.2-12]	HMF + PS (+ Std IF for n=24)	Alternating breastfeeding and PS	Phe-free PS was given every 3 hours and between these bottle feeds, breastfeeding was on demand. Standard infant formula had to be added to the diets of 24 (68.5%) breastfed infants to complete Phe intake. Volume of PS was adjusted according to blood Phe levels. If blood Phe was >360 µmol/L at three consecutive consultations, then breastfeeding was stopped and standard infant formula started.
		Santos 2011	n/r	39	n/r	*≤6 months*	HMF + PS	n/r	n/r
		Sweeney 2009	22	22	At birth	[6.9 months, 0.2-16.4]	HMF + PS	PS before on demand breastfeeding	Breastfeeding was recommenced after diagnosis once Phe levels were <600 µmol/L. The volume of Phe-free PS was altered according to the infant's blood-spot Phe levels.
		Sweeney 2011	1	1	At birth	*≥16 months*	Exclusive	On demand breastfeeding	Very mild PKU exclusively breastfed from birth. Solid foods introduced at 6 months. Between 12-16 months dietary protein was reduced due to Phe levels >400 µmol/L. At 16 months, infant received 2.5 g protein/kg/day plus one breastfeed.
		Banta-Wright 2012	75	75	n/r	6.8 months	HMF + PS	Expressed HM mixed with PS or PS before on demand breastfeeding	Breastfeeding group received HM either directly from the breast or as expressed HM delivered from a bottle and received Phe-free formula from a bottle to maintain appropriate Phe levels.
		Lamônica 2012	10	10	At birth	5.3±3.7 months [4.3, 1-14]	HMF + PS	PS before on demand breastfeeding	The procedures were based on estimating breast milk intake, with a safe margin of Phe concentration, calculating stomach volume, and initially offering formula, then breastfeeding on free demand, at every feeding. If serum Phe was 120-360 µmol/L, the prescription was kept; if it was <120 µmol/L, PS was reduced by 25%, indirectly increasing breastfeeding; if it was >360 µmol/L PS was increased by 50%.
		O'Sullivan 2013 [c]	58	45	n/r	n/r	HMF + PS	PS before on demand breastfeeding	n/r

Group of disorders	Condition	References	N of HM-fed infants		Age at HMF initiation Mean ± SD; [Median, range]	Duration of HMF Mean ± SD; [Median, range]	Exclusive vs. partial HMF after diagnosis	HMF mode of administration after diagnosis	Protocol
			Before diagnosis:	After diagnosis:					
		Sweeney 2016	n/r	1	n/r	10 months	HMF + PS	PS before on demand breastfeeding	n/r (solid foods were introduced at 5.5 months of age).
		Köse 2018	40	25	At birth	7.4±4.0 months [1-15]	HMF + PS	PS after breastfeeding	Phe-free PS given after each breastfeeding. Daily protein consumption was determined according to dietary reference intake. At least every month, all infants' prescriptions were adjusted and the volume of Phe-free formula was revised (according to serum Phe level).
		Schulpis 2019	54	0	At birth	1 month	Stopped HMF	n/a	Initially, a total of 54 newborns were exclusively (n=32) or partially (n=22) breastfed for 1 month, i.e., until diagnosis. After diagnosis, breastfeeding was replaced with standard infant formula, and all infants also started taking PS.
		Weiss 2020	3	3	At birth	n/r	HMF + PS (n=2)<br><br>HMF + PS until 38 weeks, then exclusive HMF for short period before restricting HMF and re-introducing PS (n=1)	n/r	Three pre-term infants:<br>1. Enteral feeding confined to few mL of HM due to vomiting, and parenteral nutrition was established. Diagnosis at 3 d, but Phe-containing parenteral nutrition not suspended because enteral feeding poorly tolerated (and no Phe-free parenteral feed was available). Switched parenteral feed to decrease Phe intake (3.1 g/L Phe to 0.88 g/L). Feeding of breast milk was continued to prevent necrotizing enterocolitis but was combined with a Phe-free PS. Blood Phe was normal at day 13. Tolerance of enteral feeding improved day by day. At day 17, parenteral nutrition was terminated, and infant received a breast milk fortifier, preterm formula, and Phe-free PS. With increasing weight, preterm formula and breast milk fortifier were decreased.<br>2. Enteral HM + parenteral nutrition in the first week. Due to high blood Phe, HM replaced with Phe-free PS at day 8 + additional Phe-free amino acid supplements to make up for the parenteral protein intake. Parenteral nutrition continued for an additional 2 d, limited to carbohydrates and lipids. With blood Phe in the normal range, HM was reintroduced after 3 d and steadily increased. A HM fortifier was added, Phe-free PS was stopped, and only small amounts of Phe-free amino acid supplements were required. Adequate weight gain so HM fortifiers and amino acid supplements decreased and finally stopped at 38 weeks of gestation. HM ad lib until discharge from hospital, then confined to 130 mL/kg + Phe-free PS.

Group of disorders	Condition	References	N of HM-fed infants: Before diagnosis:	After diagnosis:	Age at HMF initiation Mean ± SD; [Median, range]	Duration of HMF Mean ± SD; [Median, range]	Exclusive vs. partial HMF after diagnosis	HMF mode of administration after diagnosis	Protocol
									3. Enteral HM in the first week. Due to high blood Phe, HM was substituted with Phe-free PS at day 8. HM gradually reintroduced at day 13 + Phe-free PS. HM ad lib until expected delivery date, then restricted.
		Schulpis 2021	50	25	At birth	PKU:<br>6 days (until diagnosis)<br><br>HPA:<br>n/r	PKU:<br>Stopped HMF<br><br>HPA:<br>HMF + PS	PKU: n/a<br><br>HPA:<br>n/r	All infants were exclusively breastfed until diagnosis. In the PKU group, mothers were requested to stop breastfeeding after diagnosis by replacing it with Phe-free PS and restricted natural protein from standard infant formula.<br>Mothers in the HPA group partially replaced breastfeeding with Phe-free PS and continued breastfeeding.
		Zuvadelli 2022	37	28	At birth	7.2 months<br>[0.8-18.8]	HMF + PS	Expressed HM before PS (n=11; 39%);<br><br>PS before on demand breastfeeding (n=9; 32%);<br><br>Alternating on demand breastfeeding and PS (n=8; 29%)	The choice of the breastfeeding strategy applied was made by the dietitian taking into account mothers' preference. 19/42 (45%) required a period of "wash-out" at time of admission. Low-Phe diet supplemented with Phe-free PS was started at a mean age of 12.7 ± 3.4 days. Dietary interventions were prescribed according to the infant's phenotype (to achieve blood Phe concentrations of 120–360 µmol/L, as suggested by European guidelines).
		Rice 2023	16	16	n/r	7.6 months<br>[5.9, 0.2-24.4]	HMF + PS	PS before on demand breastfeeding (n=15)<br><br>Alternating on demand breastfeeding and PS (n=1)	n/r (Complementary feeding was commenced at a mean age of 5.3 months [range: 3.6-6.6 months])
		Rocha 2023	n/r	30	n/r	[5 months, IQR: 3-9]	HMF + PS	n/r	n/r
		Guillén-López 2024	n/r	181	n/r	n/r	HMF + PS	n/r	n/r
		Mohammad zadeh 2024	34	13	At birth	14.3±0.6 months<br>[1–24]	HMF + PS	PS after breastfeeding	Prescriptions were adjusted monthly and the volume of Phe-free PS was revised.
**Other Amino Acid**	*Maple Syrup Urine Disease (MSUD)*	Touati 2001	2	3	11±16.5 days<br>[3, 3-30]	4 and 10 months<br>(n/r; n=1)	HMF + PS	Alternating on demand breastfeeding and PS	Breastfeeding was (re-)introduced after 2 to 21 days of continuous enteral nutrition. Breastfeeding was alternated with bottles containing precursor-free PS (2 g/kg/day).

Group of disorders	Condition	References	N of HM-fed infants Before diagnosis:	After diagnosis:	Age at HMF initiation Mean ± SD; [Median, range]	Duration of HMF Mean ± SD; [Median, range]	Exclusive vs. partial HMF after diagnosis	HMF mode of administration after diagnosis	Protocol
**Disorders (AADs)**		Huner 2005	0	1	>10 days	3.3 months	HMF + PS	Controlled expressed HM mixed with PS; then PS before on demand breastfeeding	After management of the acute episode, expressed HM was introduced (1g protein/kg/day) + precursor-free PS (1.5g protein/kg/d). By 2 months, breastfeeding on demand given acceptable metabolic control. However, after 1 week, breastfeeding was terminated due to poor metabolic control and hospitalization. On-demand breastfeeding reintroduced at 3 months.
		Ross 2016	0	1	>14 days	n/r	HMF + PS	Controlled expressed HM mixed with PS	Infant was initially started on intravenous fluids after delivery due to a delay in supply of precursor-free PS. PS started on day 5. Breastfeeding avoided at birth to avoid encephalopathy and neurological sequelae. After development of skin lesions associated with isoleucine deficiency, expressed HM was added to the feeds (30% of total feed volume) to compensate for isoleucine deficiency.
		Pichler 2017	1	1	At birth	1 month	HMF + PS	Controlled expressed HM before PS	n/r
	Homocystinuria (HCU)	Dixon 2014	3	3	At birth	3 months n=1<br>*≥6 months* n=2	HMF + PS	PS before on demand breastfeeding	HM + precursor-free PS was estimated at 160 ml/kg/day (commenced at 26, 32 or 43 days). Breastfeeding was discontinued in one infant at 12 weeks into treatment due to inadequate intake of PS.
		Aktuğlu 2015	n/r	1	n/r	*≥18 months*	HMF + PS	PS before on demand breastfeeding	After diagnosis and elimination diet, precursor-free PS, and energy support were initiated (0.9 g/kg/day; 55 kcal/kg/day) with on demand breastfeeding. Infant was followed with 0.5 g/kg/day natural protein, 1.4 g/kg/day protein equivalent from PS, 95 kcal/kg/day for 18 months with breastfeeding continuation.
	Hypermethioninemia	Pichler 2017	1	1	At birth	12 months	Exclusive	On demand breastfeeding	The infant was breastfed on demand without need for PS.
	Tyrosinemia (TYR)	Daly 2005	n/r	2	11±8.5 days [11, 5-17]	5.1 and 5.3 months	HMF + PS	PS before on demand breastfeeding	Initially precursor-free PS for 1-2 days after diagnosis, then 30-70 mL was given before each breast feed from day 3. Phe supplements gradually increased from 50 to 150 mg/day Breastfeeding stopped at 22-23 weeks.
		Silva 2022	1	1	At birth	13 months	HMF + PS	PS before on demand breastfeeding	A Phe and Tyr restricted diet was initiated after diagnosis (around 1 month of age), while maintaining breastfeeding. Of 2.4 g/kg/day of total protein intake, natural protein provided 1.4 g/kg/day, preceded by precursor-free PS. Phe supplement was started due to low Tyr levels and continued until 7 months. At 13

Group of disorders	Condition	References	N of HM-fed infants: Before diagnosis:	N of HM-fed infants: After diagnosis:	Age at HMF initiation Mean ± SD; [Median, range]	Duration of HMF Mean ± SD; [Median, range]	Exclusive vs. partial HMF after diagnosis	HMF mode of administration after diagnosis	Protocol
									months breastfeeding was replaced with standard infant formula due to insufficient milk supply.
**Organic Acidemias (OAs)**	*Methylmalonic acidemia (MMA)*	Dixon 2000	1	2	At birth (n=1)<br>n/r (n=1)	0.5 and 11 months	HMF + protein-free energy supplement; then HMF + PS (n=1)<br><br>Exclusive (n=1)	Energy supplement before on demand breastfeeding; then PS before on demand breastfeeding (n=1)<br><br>Controlled expressed HM via feeding tube; then on demand breastfeeding (n=1)	n=1, at birth she was given IV dextrose, sodium benzoate and L-carnitine. She was commenced on breast feeds (1 g/kg/day) together with a protein-free energy supplement, which was gradually decreased to establish exclusive breastfeeding. However, poor weight gain and metabolic control with on demand breastfeeding, so supplemented with precursor-free PS. Poor weight gain and metabolic control continued, so breastfeeding stopped at 2 weeks and replaced with standard infant formula.<br><br>n=1, breastfeeding commenced after birth but was never well established. On day 2, he was admitted to the hospital with metabolic crisis and breastfeeding was temporarily stopped. Over several days, expressed HM was gradually reintroduced via a nasogastric tube. At 2 weeks of age, he was discharged from the hospital with exclusive on demand breastfeeding until 4 months when low protein solids were introduced, with vitamins and minerals supplements added at 6 months. Breastfeeding continued until 11 months.
		Huner 2005; Gökçay 2006	1<br>(n/r in 3)	4	At birth or 1 day (n=2)<br>n/r (n=2)	18.5±5.6 months<br>[19.5, 11-24]	HMF + PS + protein-free energy supplement (exclusive HMF for 3 months in n=1)	Controlled expressed HM mixed with PS; then PS and energy supplement before on demand breastfeeding	After management of the acute episode, natural protein intake was provided from a pre-measured expressed HM until metabolic control was established. Then, feeding with expressed HM was followed with on demand breastfeeding. Precursor-free PS and protein-free energy supplements with glucose polymers and fats were used with expressed HM or before on demand breastfeeding.
		Pichler 2017	2	2	At birth	9 and 12 months	Exclusive (n=1)<br><br>HMF + PS (n=1)	Controlled expressed HM mixed with PS before on demand breastfeeding (n=1)<br><br>On demand breastfeeding (n=1)	n/r
		Starin 2024	6	6	At birth	*≥12.3 months*<br>*[≥2-24]*	Exclusive (n=5)<br><br>HMF + PS (n=1)	On demand breastfeeding (n=2)	Asymptomatic infants (n=2) were breastfed on demand. For symptomatic infants (n=4), after acute management, controlled amounts of expressed HM were progressively introduced as total parenteral nutrition was weaned. As infants grew, they were

Group of disorders	Condition	References	N of HM-fed infants: Before diagnosis:	After diagnosis:	Age at HMF initiation Mean ± SD; [Median, range]	Duration of HMF Mean ± SD; [Median, range]	Exclusive vs. partial HMF after diagnosis	HMF mode of administration after diagnosis	Protocol
								Controlled expressed HM, then on demand breastfeeding (n=2)<br><br>Expressed HM (controlled then on demand) with (n=1) or without PS (n=1)	transitioned to on demand breastfeeding (n=2/4) or if not possible, continued to receive expressed HM (n=2/4, together with precursor-free PS in one infant).
	Propionic acidemia (PA)	Dixon 2000	0	1	2 days	*≥4 months*	HMF + protein-free energy supplement; then exclusive HMF	Energy supplement before on demand breastfeeding; then on demand breastfeeding without supplement	After acute treatment (IV dextrose, L-carnitine and sodium benzoate), oral feeds were started including a measured volume of protein-free energy supplement (50% of estimated fluid requirement) before each breast feed. Volume of supplement was reduced over the next few days until the infant was fully breastfed, and medications continued orally. At 4 months, protein-free solid foods were introduced. Breast feeds were decreased as protein-containing foods were introduced and increased.
		Huner 2005; Gökçay 2006	0	1	10 days	3.5 months	HMF + PS	Controlled expressed HM mixed with PS; then PS before on demand breastfeeding	After management of the acute episode, natural protein intake was provided from a pre-measured expressed HM with precursor-free PS until metabolic control was established. Then, feeding with expressed HM was followed with on demand breastfeeding with precursor-free PS.
		Pichler 2017	3	3	At birth	12.3±6.5 months; [12, 6-19]	Exclusive (n=1)<br><br>HMF + PS (n=2)	On demand breastfeeding (n=1)<br><br>Controlled expressed HM mixed with PS (n=2)	n/r
		Starin 2024	7	7	At birth	*≥8.1 months [1-≥23]*	Exclusive (n=2)<br><br>HMF + PS (n=3)<br><br>HMF + protein-free energy supplement (n=2)	On demand breastfeeding (n=1)<br><br>On demand breastfeeding with additional expressed HM and PS (n=1)<br><br>Controlled expressed HM with energy supplement, then on demand breastfeeding (n=1)	Asymptomatic infants (n=4) were breastfed on demand with (n=1/4) or without additional expressed HM and precursor-free PS (n=1/4), or could not be directly breastfed and received expressed HM in controlled amounts (n=1/4) or ad lib (n=1), both with precursor-free PS. For symptomatic infants (n=3), after acute management, controlled amounts of expressed HM were progressively introduced (combined with a protein-free energy supplement in n=2/3) as total parenteral nutrition was weaned. Only one infant could transition to on demand breastfeeding.

Group of disorders	Condition	References	N of HM-fed infants Before diagnosis:	After diagnosis:	Age at HMF initiation Mean ± SD; [Median, range]	Duration of HMF Mean ± SD; [Median, range]	Exclusive vs. partial HMF after diagnosis	HMF mode of administration after diagnosis	Protocol
								Controlled expressed HM only (n=1), with PS (n=1), or with energy supplement (n=1)<br><br>On demand expressed HM with PS (n=1)	
	Glutaric acidemia type 1 (GA1)	Huner 2005; Gökçay 2006	n/r	2	n/r	6 and 11 months	HMF + PS	Controlled expressed HM mixed with PS, then PS before on demand breastfeeding (n=1)<br><br>PS before on demand breastfeeding (n=1)	After management of the acute episode, on-demand breastfeeding with precursor-free PS. One infant first received expressed HM for 2 weeks.
		Fitzachary 2015	n/r	2	n/r	*≥6 months*	HMF + PS	PS before on demand breastfeeding	Lysine intake was limited by giving a measured volume of precursor-free PS before HMF; HM + PS was estimated as 170 ml/kg/day.
		Pichler 2017	2	2	At birth	2.5 and 5 months	HMF + PS	Controlled expressed HM mixed with PS before on demand breastfeeding	Breastfeeding on demand with additional precursor-free PS given in a small amount of expressed breast milk via a standard bottle immediately before on demand breastfeeding.
	Isovaleric acidemia (IVA)	Huner 2005; Gökçay 2006	0	2	>8 days	1.5 and 10 months	HMF + PS + protein-free energy supplement	Controlled expressed HM mixed with PS (n=1)<br><br>Controlled expressed HM mixed with PS, then PS before on demand breastfeeding (n=1)	After management of the acute episode, natural protein intake was provided from a pre-measured expressed HM with precursor-free PS until metabolic control was established. Then, breastfeeding on demand was successful in one infant, but breastfeeding was terminated in the other infant due to insufficient HM supply.
	Malonic acidemia (MA)	Pichler 2017	1	1	At birth	6 months	Exclusive	On demand breastfeeding	n/r
**Urea Cycle Disorders (UCDs)**	*Citrullinemia*	Kamper 2001	n/r	1	7 days	*≥8 months*	HMF + PS	n/r	Initial management (4 d) with sodium benzoate, arginine hydrochloride, L-carnitine and parenteral nutrition; natural protein was introduced on the 2nd day after admission with additional EAA-PS. On day 7, controlled breastfeeding was started while continuing administration of phenylbutyrate, L-arginine, L-carnitine and EAA-PS.
	Ornithine transcarbamylase (OTC) deficiency	Rawlinson 2000	2	2	At birth	7 and 8 months	HMF + protein-free energy supplement; then exclusive HMF (n=1)	Controlled expressed HM with energy supplement; then on demand breastfeeding without supplement (n=1)	n=1, breastfeeding stopped on day 2 due to hyperammonaemia and medications were commenced. Expressed HM was introduced on day 3 (0.5 g/kg/day) and gradually increased up to 2.5 g/kg/day with concomitant use of a protein-free. high calorie fluid and vitamin and mineral supplements. On

Group of disorders	Condition	References	N of HM-fed infants		Age at HMF initiation Mean ± SD; [Median, range]	Duration of HMF Mean ± SD; [Median, range]	Exclusive vs. partial HMF after diagnosis	HMF mode of administration after diagnosis	Protocol
			Before diagnosis:	After diagnosis:					
							Exclusive (n=1)	On demand breastfeeding (n=1)	demand HMF without calorie supplementation established by 2 weeks. Low protein weaning at 4 months. Breastfeeding until 8 months.<br><br>n=1, exclusive on demand breastfeeding from birth. Low protein weaning at 4 months. Breastfeeding until 7 months.
		Huner 2005	0	1	5 days	1.5 months	HMF + PS	Controlled expressed HM with PS; then on demand breastfeeding	After management of the acute episode, expressed HM for one month mixed with PS, then on demand breastfeeding at day 40 for 2 weeks.
		Pichler 2017	1	1	At birth	9 months	Exclusive	On demand breastfeeding	n/r
		Burfield 2024	1	1	At birth	3.3 months	HMF + PS	Controlled expressed HM before and/or mixed with PS	50–53% total protein from EAA-PS, combined with expressed HM until 13 weeks of age and standard infant formula thereafter.
	Arginase deficiency (AD)	Pichler 2017	2	2	At birth	2 and 6 months	Exclusive	On demand breastfeeding (n=1)<br><br>Controlled expressed HM (n=1)	n/r
	Arginino-succinate lyase (ASL) deficiency	Dixon 2000	2	2	At birth	3 and ≥4 months	HMF + protein-free energy supplement (n=1)<br><br>HMF + protein-free energy supplement; then exclusive HMF (n=1)	Controlled expressed HM mixed with energy supplement via feeding tube; then on demand breastfeeding without supplement (n=1)<br><br>Controlled expressed HM mixed with energy supplement via feeding tube; then energy supplement before on demand breastfeeding (n=1)	n=1; after acute treatment (IV dextrose and sodium benzoate), infant commenced feeds providing 0.4 g/kg expressed HM with added carbohydrates, fat, vitamins and minerals, fed at 2 hourly intervals via a nasogastric tube. The protein intake was gradually increased and exclusive breastfeeding on demand was established at 2 weeks with medications (arginine and sodium phenylbutyrate). At 4 months, weaning was commenced with very low protein foods, and breastfeeding was reduced concomitantly (e.g., giving 2 g protein from solids instead of each breast feed).<br><br>n=1; after birth, an acute treatment (IV dextrose, arginine and sodium benzoate) was commenced together with demand breastfeeding. On day 5, breastfeeding was stopped temporarily due to high ammonia levels, and he was treated with an emergency regimen. On day 6, protein-free energy supplement was given before breastfeeds, providing 20% of fluid requirement. He was discharged on day 12, with 120 ml/kg/day energy supplement + breastfeeds supplemented with arginine and sodium

Group of disorders	Condition	References	N of HM-fed infants: Before diagnosis:	N of HM-fed infants: After diagnosis:	Age at HMF initiation Mean ± SD; [Median, range]	Duration of HMF Mean ± SD; [Median, range]	Exclusive vs. partial HMF after diagnosis	HMF mode of administration after diagnosis	Protocol
									benzoate until 3 months, then he was switched to bottle feeding upon mother's request.
		Burfield 2024	2	2	At birth	>6.7 and >7.6 months	HMF + PS	Controlled expressed HM mixed with PS or PS before on demand breastfeeding	EAA-PS in conjunction with HM. 40–50% of total protein was provided by PS. Both infants received expressed HM and nursed at the breast, making estimations of actual HM intake difficult.
	Carbamoyl phosphate synthetase 1 (CPS 1) deficiency	Bzduch 2019	1	2	At birth (n=1);<br>2 weeks (n=1)	2 and 3 months	HMF + protein-free energy supplement (n=1)<br><br>Exclusive (n=1)	Controlled expressed HM with energy supplement; then on demand breastfeeding with energy supplement (n=1)<br><br>Controlled expressed HM; then on demand breastfeeding without supplement (n=1)	n=1; expressed HM (2 weeks) started with intake 1.6 g protein/kg/day was followed with on demand HMF (up to 3 months) with supplementation of energy, L-citrulline, L-arginine, sodium benzoate and sodium phenylbutyrate.<br><br>n=1; from birth on expressed HM with protein intake 1.3 g protein/kg/day followed with on demand HMF nearly 2 months.
**Fatty Acid Oxidation Disorders (FAODs)**	*Medium-chain acyl-CoA dehydrogenase (MCAD) deficiency*	Roe 1986	2	1 [d]	At birth	n/r	Exclusive	n/r	Continued breastfeeding with a carnitine supplement of 12.5 mg/kg 4x daily.
		Hsu 2008 [e]	11	11	At birth	n/r	Exclusive	On demand breastfeeding	n/r (parents were advised to maintain frequent feedings)
		Ahrens-Nicklas 2016	34	34	At birth	n/r	Exclusive; n=24 (70.6%)<br><br>HMF + standard infant formula; n=10 (29.4%)	On demand breastfeeding	n/r
		Petropoulou 2017	1	1	At birth	2 months	HMF + pre-term formula or standard infant formula	Controlled expressed HM with pre-term or standard infant formula	0-5 d: expressed HM with preterm formula via nasogastric tube. On day 5, preterm formula was replaced with standard infant formula (50% + 50% expressed HM). At discharge (41 weeks) high energy infant formula was initiated and was used exclusively for 2 weeks (stopped using expressed HM), which was then replaced with standard infant formula until weaning at 6 months.
	Long-chain hydroxyacyl-coA dehydrogenase (LCHAD) deficiency	Pichler 2017	3	3	At birth	3.5±1.3 months; [3.0, 2.5-5]	Exclusive; n=2 (66.7%)<br><br>HMF + MCT formula; n=1 (33.3%)	On demand breastfeeding (n=2)<br><br>Controlled expressed HM with MCT formula (n=1)	n/r

Group of disorders	Condition	References	N of HM-fed infants: Before diagnosis:	N of HM-fed infants: After diagnosis:	Age at HMF initiation Mean ± SD; [Median, range]	Duration of HMF Mean ± SD; [Median, range]	Exclusive vs. partial HMF after diagnosis	HMF mode of administration after diagnosis	Protocol
	Very long-chain acyl-CoA dehydrogenase (VLCAD) deficiency	Pichler 2017	1	1	At birth	1 month	Exclusive	On demand breastfeeding	n/r
	Mixed (LCHAD or VLCAD deficiency)	Hussa 2006	n/r	4	At birth	[1-4 months]	Exclusive; n=1 (25%)<br><br>HMF + low fat formula; n=3 (75%)	Controlled expressed HM with low fat formula (n=2)<br><br>On demand breastfeeding (n=1)<br><br>Low fat formula before on demand breastfeeding (n=1)	n=2 infants were given calculated amounts of expressed HM in conjunction with a low LCT diet, n=1 was exclusively breastfed for the first month, n=1 was first given calculated amounts of a low-fat formula and then breastfed ad lib for the first 3 months.<br>All formula/HM combinations were designed to provide approximately 40% of calories from fat, 10-20% calories from LCT.
	Carnitine-acylcarnitine translocase (CACT) deficiency	Kritzer 2020	1	1	At birth	*>12 months*	HMF + MCT formula + protein and carbohydrate supplements	Controlled expressed skimmed HM mixed with MCT formula and protein and carb supplements	A combination of expressed modified-fat (skimmed) HM, supplemented with triheptanoin (4 g/kg; 73% of total fat), protein and carbohydrate modulars [100 mL modified-fat HM = 0.24 g fat + 1.22 g protein + 7.18 g carbs]. Soybean oil was used to provide essential fatty acids but was replaced by walnut oil at 2.5 months of age due to essential fatty acid deficiency.<br>Discharged at day 33 with 8x86 mL of formulated skimmed HM: ~700 mL modified-fat HM, 5 mL soybean oil, 1.5 tablespoon protein modular, 40 g carb modular, 17.6 mL triheptanoin (Total; 91.4 g carb. + 11.5 g protein + 16.9 g MCT + 6.3 g long chain triglycerides).
	Carnitine palmitoyltransferase II (CPT II) deficiency	Pichler 2017	1	1	At birth	n/r	Exclusive	On demand breastfeeding	n/r
**Other**	*Galactose epimerase (GALE) deficiency*	Pichler 2017	2	2	At birth	1 and 4 months	Exclusive	On demand breastfeeding	n/r

EAA, essential amino acids; LCT, long chain triglycerides; MCT, medium chain triglycerides; HM, human milk; HMF, human milk feeding (i.e., receiving expressed/pumped HM or at the breast); PS, protein substitute; SD, standard deviation
[a] Mean or median only calculated if n≥3. For n=1 or n=2, the actual duration of HMF is provided (if reported by the authors).
[b] Nielsen, 2005: In 3 infants, HMF was temporarily discontinued until blood Phe level was <900 µmol/L, whereas in the remaining 3 infants, the clinicians allowed HMF without any breaks despite the Phe level was >900µmol/L.
[c] O'Sullivan, 2013: Number of infants receiving HM after diagnosis was not clearly reported; however, for 13 of the 58 HM-fed infants, median HMF duration was 5 days and therefore it was assumed that they stopped HMF at diagnosis.
[d] Roe, 1986: Diagnosis occurred in infancy only for one sibling who continued to be breastfed. For the other sibling, diagnosis occurred outside of the HMF period, at 3.5y.
[e] Hsu, 2008: Only 15/20 infants were included because the feeding type was unknown for the remaining 5 infants.

Supplementary Table 4. Short- and long-term growth of infants with an inherited metabolic disorder who have received human milk.

Group of disorders	Condition	N of studies and infants	References	N of infants: HMF vs. Std IF *(if applicable)*	Duration of HMF; Mean [range]	Follow-up/ Time of assessment	Key findings	Protocol for assessment
**Amino acid disorders (AADs)**	*Phenylketonuria (PKU)*	N=15<br><br>HMF: 507<br>Std IF: 383<br>Total: 890	McCabe 1989	18 vs. 10	8.9 months [1.5-25.5] (n=16)<br>≥6 months (n=2)	6 months	**Adequate growth**<br>**Weight, height & HC: No significant difference between HMF and Std IF**	**Weight, height, HC:** Monthly
			Greve 1994	9 vs. 4	3 months [1.3-5.2] (n=5)<br>≥6 months (n=4)	6 months	**Adequate growth**<br>**Weight gain: No significant difference between HMF and Std IF**	**Weight:** Weekly
			Miller 1994	HMF: 1	≥3 months	3 months	**Adequate growth**	n/r
			Motzfeldt 1999	74 vs. 9	7 months [1-16]	n/r	**Adequate growth**<br>**Height, weight & HC:** Within the normal range for age on Norwegian growth chart	**Weight:**<br>3x a week at initiation of diet<br>**Height & HC:**<br>At admission; monthly thereafter
			Davidson 2000	33 vs. 19	≤15 months	5 & 10 years	**Adequate growth**<br>**Growth: comparable or superior HMF vs. Std IF**	n/r
			Francis 2000	HMF: 1	≥5 months	5 months	**Adequate growth**	n/r
			Cornejo 2003	HMF: 19	≥6 months (n=14)<br><6 months (n=5)	6 months	**Adequate growth**<br>**Height, weight:** Z-scores within ±2 SDs over the 6 months follow-up.	**Weight & Height:**<br>Monthly<br><br>z-scores were calculated, the National Center for Health Statistics (NCHS) growth charts were used as a standard.
			van Rijn 2003	9 vs. 9	2.5 months [1.8-8.3]	7 months	**Adequate growth**<br>Better growth when compared to the average Dutch PKU population<br>**Height, weight & HC: No significant difference between HMF and Std IF**	**Weight, height, HC:**<br>Frequency of monitoring n/r
			Kanufre 2007	35 vs. 35	7.5 months [1.2-12]	12 months	**Adequate growth**<br>Initial assessment: 2/35 of HM-fed vs. 1/35 of formula-fed were underweight (<-2SD); 1/35 of HM-fed vs. 1/35 of formula-fed were stunted (<-2SD)	**Weight, height, HC:**<br>**Initial assessment:**<br>Within the 1st month of life

Group of disorders	Condition	N of studies and infants	References	N of infants: HMF vs. Std IF *(if applicable)*	Duration of HMF; Mean [range]	Follow-up/ Time of assessment	Key findings	Protocol for assessment
							Final assessment: 2/35 of HM-fed infants were underweight/stunted<br>**Weight & height:** Good progress in both groups<br>**HC:** Significantly improved in both groups<br>**No significant difference between HMF and Std IF**	**Final assessment**:<br>HM-fed: Age at cessation of HMF;<br>Std IF: Same age as their matched HM-fed infant.
			Sweeney 2009	22 vs. 5	Median 6.9 months [0.2-16.4]	n/r	**Adequate growth**	n/r
			Santos 2011	39 vs. 39	≤6 months	≤ 6 months	**Adequate growth**<br>**Weight, height, HC:**<br>Infants in both groups had similar growth and presented z-scores within the normal range (z-score ≥-2).	**Weight, height, HC:** At baseline and at the end of study<br><br>z-scores were calculated, the National Center for Health Statistics (NCHS) growth charts were used as a standard.
			Köse 2018	25 vs. 16	7.4 months [1-15]	12 months	**Adequate growth**<br>**Weight: Similar at birth; significantly lower at 6 months in the HMF group; significantly higher at 1y in the HMF group**<br>**Mean daily weight gain during HMF period: Significantly lower in the HMF group**<br>**Mean monthly weight gain at the end of 1y: Significantly higher in the HMF group**	**Weight:**<br>Monthly (at least)
			Zuvadelli 2022	28 vs. 14	7.2 months [0.8-18.8]	12 months	**Adequate growth**<br>**Weight & height:** Lower percentiles in both groups upon admission<br>**Weight gain rate:** Both groups moved in the direction of the median; **significantly higher in the Std IF group**<br>**Longitudinal GR:** Both groups moved in the direction of the median; **no significant difference between HMF and Std IF**<br>**No significant difference in growth according to the type of HMF administration method**	**Weight, height, GR:**<br>Every 3 months

Group of disorders	Condition	N of studies and infants	References	N of infants: HMF vs. Std IF *(if applicable)*	Duration of HMF; Mean [range]	Follow-up/ Time of assessment	Key findings	Protocol for assessment
			Guillén-López 2024	181 vs. 202	n/r	2 years	**BMI z-score: No significant difference between HMF and Std IF** (p = 0.350) **Height/age z-score: Significantly better HMF vs. Std IF** (Median [range]: −0.72 [−1.09,−0.32] vs. −0.89 [−1.7, −0.35], p = 0.002)	**Weight, height:** n/r
			Mohammadzadeh 2024	13 vs. 21	14.3 months [1–24]	< 2 years	**Adequate growth** **Weight, height, HC:** Both groups were within the normal ranges of the Iranian standard growth centile charts **No significant difference between HMF and Std IF**	**Weight, height, HC:** Monthly
**Other Amino Acid Disorders**	*Maple Syrup Urine Disease (MSUD)*	N=3 HMF: 5	Touati 2001	HMF: 3	4 months (n=1) 11 months (n=1) n/r (n=1)	n/r	**Adequate growth**	n/r
			Huner 2005	HMF: 1	3.3 months	3 months	**Adequate growth**	**Weight, height, HC:** Weekly or biweekly
			Pichler 2017	HMF: 1	1 month	5 years	**Adequate growth** **Weight, height:** >3rd centile	**Weight, height:** Biweekly
	Homocystinuria (HCU)	N=1 HMF: 3	Dixon 2014	HMF: 3	3 months (n=1) ≥6 months (n=2)	6 months	**Adequate weight gain**	**Weight:** Frequency of monitoring n/r
	Hypermethioninemia	N=1 HMF: 1	Pichler 2017	HMF: 1	12 months	5 years	**Adequate growth** **Weight, height:** >3rd centile	**Weight, height:** Every 3 months
	Tyrosinemia (TYR)	N=2 HMF: 3	Daly 2005	HMF: 2	5.1 months (n=1) 5.3 months (n=1)	5 months	**Adequate growth** **Weight, height:** 25th centile	**Weight, height:** Frequency of monitoring n/r

Group of disorders	Condition	N of studies and infants	References	N of infants: HMF vs. Std IF *(if applicable)*	Duration of HMF; Mean [range]	Follow-up/ Time of assessment	Key findings	Protocol for assessment
			Silva 2022	HMF: 1	13 months	14 months	**Adequate growth at follow-up**<br>At initiation of dietary treatment: Poor growth due to low Phe levels (around 2 months)<br>At follow-up: On demand HMF was continued until 13 months with Phe supplementation (between 2-7 months) and use of a medical formula; at 14 months, growth was normal	n/r
**Organic acidemias (OAs)**	*Methylmalonic acidemia (MMA)*	N=4<br>HMF: 8	Dixon 2000	HMF: 2	2 weeks (n=1)<br>11 months (n=1)	2 weeks (n=1)<br>4 months (n=1)	n=1, **poor weight gain; HMF discontinued after 2 weeks. Improvement after switching to Std IF**<br>n=1, **adequate growth. Weight, height:** 25th centile	**Weight, height:**<br>Frequency n/r
			Huner 2005; Gökçay 2006	HMF: 4	18.5 months [11-24]	[11-24 months]	**Adequate growth**<br>**Weight, height, HC:** Within 3rd - 97th centiles	**Weight, height, HC:**<br>Weekly or biweekly after discharge
			Pichler 2017	HMF: 2	9 months (n=1)<br>12 months (n=1)	1 year (n=1)<br>2 years (n=1)	**Adequate growth**<br>**Height:** >3rd centile<br>**Weight:** Transient poor weight gain in n=1, **adequate weight gain** was quickly re-achieved after support from dietitian and lactation consultant was intensified	**Weight, height:**<br>2-4 weeks intervals
	Propionic acidemia (PA)	N=4<br>HMF: 5	Dixon 2000	HMF: 1	≥4 months	4 months	**Adequate growth**<br>**Weight:** 25th-50th centile<br>**Height:** 75th centile	**Weight, height:**<br>Frequency n/r
			Huner 2005; Gökçay 2006	HMF: 1	3.5 months	4 months	**Adequate growth**<br>**Weight, height, HC:** Within 3rd - 97th centiles	**Weight, height, HC:**<br>Weekly or biweekly after discharge
			Pichler 2017	HMF: 3	12.3 months [6-19]	[2-11 years]	**Adequate growth**<br>**Weight, height:** >3rd centile	**Weight, height:**<br>2-4 weeks interval

Group of disorders	Condition	N of studies and infants	References	N of infants: HMF vs. Std IF *(if applicable)*	Duration of HMF; Mean [range]	Follow-up/ Time of assessment	Key findings	Protocol for assessment
	Glutaric acidemia Type 1 (GA1)	N=4<br>HMF: 6	Huner 2005; Gökçay 2006	HMF: 2	6 months (n=1)<br>11 months (n=1)	6 months (n=1)<br>11 months (n=1)	At diagnosis:<br>**Weight:** n=1 was **underweight** (<3rd centile)<br>**HC: Above normal** in n=1 (>97th)<br>**Height: Adequate** (within 3rd - 97th centiles)<br>At follow-up:<br>Weight improved in n=1 who was underweight at diagnosis<br>**Weight, height: Adequate** (within 3rd - 97th centiles)<br>**HC: Above normal in both infants** (>97th)	**Weight, height, HC:** Weekly or biweekly after discharge
			Fitzachary 2015	HMF: 2	≥6 months	6 months	**Adequate growth**	n/r
			Pichler 2017	HMF: 2	2.5 months (n=1)<br>5 months (n=1)	2 years (n=1)<br>4 years (n=1)	**Height: Poor linear growth** in n=1 (<3rd centile)<br>**Weight:** Transient poor weight gain in n=1, **adequate weight gain** was quickly re-achieved after support from dietitian and lactation consultant was intensified	**Weight, height:** 2-4 weeks interval
	Isovaleric acidemia (IVA)	N=2<br>HMF: 2	Huner 2005; Gökçay 2006	HMF: 2	1.5 months (n=1)<br>10 months (n=1)	1.5 months (n=1)<br>10 months (n=1)	**Adequate growth at diagnosis, one obese infant at follow-up**<br>At diagnosis: **Weight, height, HC:** Within 3rd - 97th centiles<br>At follow-up: **Weight:** n=1 obese (>97th centile), **Height, HC:** Within 3rd - 97th centiles	**Weight, height, HC:** Weekly or biweekly after discharge
	Malonic acidemia (MA)	N=1<br>HMF: 1	Pichler 2017	HMF: 1	6 months	1 years	**Adequate growth**<br>**Weight, height:** >3rd centile	**Weight, height:** 2-4 weeks interval
**Urea Cycle Disorders (UCDs)**	*Citrullinemia*	N=1<br>HMF: 1	Kamper 2001	HMF: 1	≥8 months	8 months	**Adequate growth**	n/r
	Ornithine transcarbamylase (OTC) deficiency	N=2<br>HMF: 2	Pichler 2017	HMF: 1	9 months	6 years	**Adequate growth**<br>**Weight, height:** >3rd centile	**Weight, height:** 2-4 weeks interval
			Burfield 2024	HMF: 1	3.3 months	5.5 months	**Adequate growth**	**Weight, height, HC:** 2-4 weeks interval

Group of disorders	Condition	N of studies and infants	References	N of infants: HMF vs. Std IF *(if applicable)*	Duration of HMF; Mean [range]	Follow-up/ Time of assessment	Key findings	Protocol for assessment
	Arginase deficiency (AD)	N=1<br>HMF: 2	Pichler 2017	HMF: 2	2 months (n=1)<br>6 months (n=1)	3 years (n=1)<br>10 years (n=1)	n=1, **adequate height** (>3rd centile) **but underweight** (<3rd centile)<br>n=1, **adequate growth** (>3rd centile)	**Weight, height:** 2-4 weeks interval
	Argininosuccinate lyase (ASL) deficiency	N=2<br>HMF: 4	Dixon 2000	HMF: 2	3 months (n=1)<br>≥4 months (n=1)	3 months (n=1)<br>4 months (n=1)	**Adequate growth**<br>**Weight:** 25th centile, n=1; 50th centile, n=1<br>**Height:** 9th-25th centile, n=1; 25th-50th, n=1	n/r
			Burfield 2024	HMF: 2	>6.7 months (n=1)<br>>7.6 months (n=1)	6.7 months (n=1)<br>7.6 months (n=1)	**Adequate growth**	**Weight, height, HC:** 2-4 weeks interval
	Carbamoyl phosphate synthetase 1 (CPS 1) deficiency	N=1<br>HMF: 1	Bzduch 2019	HMF: 1 [a]	3 months	3 years	**Adequate growth**	**Growth rate:** Frequency of monitoring n/r
**Fatty Acid Oxidation Disorders (FAODs)**	*Medium-chain acyl-CoA dehydrogenase (MCAD) deficiency*	N=1<br>HMF: 1	Petropoulou 2017	HMF: 1	2 months	37 weeks gestational age & 4 years	**Poor growth**<br>**Weight:** Poor weight gain (z-score -2.9 & -3.00 SDs at 37 weeks gestational age and 4y, respectively)<br>**Height:** Height z-score was -2.54 SDs at long-term follow-up	**Weight, height:** Frequency of monitoring n/r
	Long-chain hydroxyacyl-coA dehydrogenase (LCHAD) deficiency	N=1<br>HMF: 3	Pichler 2017	HMF: 3	3.5 months [2.5-5]	[0.5-10 years]	**Height: Poor linear growth** in n=1 (<3rd centile)<br>**Weight: Adequate**.	**Weight, height:** Biweekly
	Very long-chain acyl-CoA dehydrogenase (VLCAD) deficiency	N=1<br>HMF: 1	Pichler 2017	HMF: 1	1 month	6 months	**Adequate growth**<br>**Weight, height:** >3rd centile	**Weight, height:** Biweekly
	Mixed (LCHAD or VLCAD)	N=1<br>HMF: 4	Hussa 2006	HMF: 4	[1-4 months]	n/r	**Adequate growth**	n/r
	Carnitine-acylcarnitine translocase (CACT) deficiency	N=1<br>HMF: 1	Kritzer 2020	HMF: 1	>12 months	12 months	**Adequate growth**<br>**Height, weight:** Age specific z-scores within 60th-70th centiles	**Weight, height:** Every 2 months

Group of disorders	Condition	N of studies and infants	References	N of infants: HMF vs. Std IF *(if applicable)*	Duration of HMF; Mean [range]	Follow-up/ Time of assessment	Key findings	Protocol for assessment
	Carnitine palmitoyltransferase II (CPT II) deficiency	N=1<br><br>HMF: 1	Pichler 2017	HMF: 1	n/r	4 years	**Adequate growth**<br>**Weight, height:** >3rd centile	**Weight, height:** Biweekly
**Others**	*Galactose epimerase (GALE) deficiency*	N=1<br><br>HMF: 2	Pichler 2017	HMF: 2	1 months (n=1)<br>4 months (n=1)	9 years (n=1)<br>5 years (n=1)	**Adequate growth**<br>**Weight, height:** >3rd centile	**Weight, height:** Monthly

Abbreviations: GR: growth rate; HC, head circumference; HMF, human milk feeding; Min, minimum; n/r, not reported; N, number; Phe, phenylalanine; SD, standard deviation; Std IF, standard infant formula.
[a] Bzduch 2019: Growth data was not reported for the sibling.

Supplementary Table 5. Metabolic control of infants with an inherited metabolic disorder during the human milk feeding period.

Group of disorders	Condition	N of studies and infants	References	N of infants: HMF vs. Std IF *(if applicable)*	Duration of HMF Mean [range]	Follow-up/ Time of assessment	Key findings	Protocol for assessment
**Amino acid disorders (AADs)**	*Phenylketonuria (PKU)*	N=25<br><br>HMF: 671<br>Std IF: 556<br>Total: 1227	Francis 1981	HMF: 6	n/r	n/r	**Acceptable metabolic control**	**Blood Phe:**<br>Daily for the 1st week, 2x a week until stabilised, weekly thereafter
			McCabe 1989	18 vs. 10	8.9 months [1.5-25.5] (n=16)<br>≥6 months (n=2)	6 months	**No significant difference in metabolic control between HMF and Std IF**<br>Mean ± SD serum Phe HMF vs. Std IF groups:<br>Month 1: 993 ± 285 vs 1084 ± 395 µmol/L<br>Month 2: 478 ± 145 vs 454 ± 139 µmol/L<br>Month 3: 472 ± 194 vs 478 ± 206 µmol/L<br>Month 4: 617 ± 176 vs 630 ± 218 µmol/L<br>Month 5: 599 ± 151 vs 593 ± 218 µmol/L<br>Month 6: 557 ± 157 vs 684 ± 254 µmol/L<br><br>Blood Tyr: no significant differences between HMF and Std IF groups either	**Blood Phe & Tyr:**<br>Frequency of monitoring n/r<br>Postprandial specimens were obtained (at least 2h)<br>Monthly clinic visit
			Greve 1994	9 vs. 4	3 months [1.3-5.2] (n=5)<br>≥6 months (n=4)	6 months	**Acceptable metabolic control**<br>**No significant difference in metabolic control between HMF and Std IF**<br>Phe concentrations for both HMF and Std IF groups were below the accepted upper limit of 480 µmol/L for serum Phe	**Blood Phe:**<br>2x a week until metabolic control achieved weekly thereafter
			Miller 1994	HMF: 1	≥3 months	3 months	**Acceptable metabolic control**<br>Blood Phe concentrations remained <327 µmol/L	**Blood Phe:**<br>Frequency of monitoring n/r
			Duncan 1997	HMF: 1	11 months	5 months	**1st month: Blood Phe concentrations above the acceptable range** (180-360 µmol/L)<br>**During FU: acceptable metabolic control**	**Blood Phe:**<br>Weekly up to 5 months biweekly thereafter
			Motzfeldt 1999	74 vs. 9	7 months [1-16]	n/r	**Acceptable metabolic control**<br>Serum Phe reached target concentrations (120-400 µmol/L) in the 1st week it was easily controlled during the HMF period.<br>No information reported for infants receiving Std IF as their Phe source.	**Blood Phe:**<br>At initiation of diet:<br>3x a week<br>During follow-up<br>Weekly

Group of disorders	Condition	N of studies and infants	References	N of infants: HMF vs. Std IF *(if applicable)*	Duration of HMF Mean [range]	Follow-up/ Time of assessment	Key findings	Protocol for assessment
			Davidson 2000	33 vs. 19	≤15 months	5 & 10 years	**Acceptable metabolic control**<br>Phenylalanine Exposure Index was comparable or superior to age-matched Std IF fed infants with PKU.	**Blood Phe:**<br>Frequency of monitoring n/r (Phenylalanine Exposure Index definition n/r)
			Francis 2000	HMF: 1	≥5 months	[0-5 months]	**Acceptable metabolic control**	**Blood Phe:**<br>Initially twice weekly, then once weekly
			Cornejo 2003	HMF: 19	≥6 months (n=14)<br><6 months (n=5)	6 months	n=15: **Acceptable metabolic control** (blood Phe level <480 µmol/L),<br>n=4: **Intermittently high blood Phe levels** (600-720 µmol/L)	**Blood Phe:**<br>Weekly
			van Rijn 2003	9 vs. 9	2.5 months [1.8-8.3]	n/r	**Acceptable metabolic control**<br>**No significant difference in metabolic control between HMF and Std IF**<br>Median plasma Phe HMF vs. Std IF groups:<br>170 vs. 181 µmol/L<br>% Plasma Phe 120-360 µmol/L HMF vs. Std IF groups:<br>56% vs. 59%<br>% Plasma Phe <120 µmol/L HMF vs. Std IF groups:<br>31% vs. 26%<br>% Plasma Phe >360 µmol/L HMF vs. Std IF groups:<br>6% vs. 4%	**Blood Phe:**<br>Daily during 1st week 2x a week, and biweekly when concentrations were stable within the therapeutically aimed range
			Nielsen 2005	HMF: 6	n/r	n/r	**Acceptable metabolic control**<br>Group 1: infants who temporarily discontinued HMF after diagnosis until Phe concentrations decreased to <900 mmol/L<br>Group 2: continuous HMF (even if Phe concentrations at diagnosis were >900 mmol/L)<br>Mean ± SD [median, range] blood Phe concentrations:<br>Group 1 (n=3): 204 ± 19.4 [200, 188-226] mmol/L<br>Group 2 (n=3): 268 ± 51.4 [286, 210-308] mmol/L<br>Overall, mean Phe concentrations did not differ between infants on continuous HMF vs. infants who had to temporarily discontinue HMF	**Blood Phe:**<br>Frequency of monitoring n/r

Group of disorders	Condition	N of studies and infants	References	N of infants: HMF vs. Std IF *(if applicable)*	Duration of HMF Mean [range]	Follow-up/ Time of assessment	Key findings	Protocol for assessment
			Kanufre 2007	35 vs. 35	7.5 months [1.2-12]	12 months	**Acceptable metabolic control**<br>**No significant difference in metabolic control between HMF and Std IF**<br>Blood Phe within acceptable range: 87% of blood Phe in HMF vs. 74% in Std IF groups (not significant)<br>Median time taken for Phe concentrations to return to acceptable range: 8 days in HMF vs. 7 days in Std IF groups (not significant)	**Blood Phe:**<br>Weekly until 6 months biweekly 6-12 months
			Sweeney 2009	22 vs. 5	Median 6.9 months [0.2-16.4]	n/r	**Acceptable metabolic control**<br>(aim blood Phe ≤350 µmol/L whole blood)<br>No information reported for infants receiving Std IF as their Phe source	**Blood Phe:**<br>Twice weekly until 3 months then weekly
			Santos 2011	39 vs. 39	≤6 months	≤6 months	**Acceptable metabolic control**<br>Infants maintained blood Phe ≤360 µmol/L during the study period	**Blood Phe:**<br>Frequency of monitoring n/r
			Sweeney 2011	HMF: 1	≥16 months	16 months	**Acceptable metabolic control**<br>Mean [range] blood Phe and Tyr concentrations:<br>0-1 months: 320 µmol/L [99-431] (Phe) and 86.5 µmol/L [51-113] (Tyr)<br>1-3 months: 271µmol/L [251-352]<br>3-12 months: 283 µmol/L [149-422]<br>12 months: 545 µmol/L (Phe), 123 µmol/L (Tyr)<br>12-16 months: 404 µmol/L [117-600], higher concentrations were at times of illness<br>16-months: 333 µmol/L (Phe), 78 µmol/L (Tyr)<br>Overall, blood Phe was within target range (<360 µmol/L) except 12-16 months period	**Blood Phe:**<br>Days 3, 10, 19, 28<br>1-3 months: Weekly<br>3-12 months: Monthly<br>12-16 months: Fortnightly

Group of disorders	Condition	N of studies and infants	References	N of infants: HMF vs. Std IF *(if applicable)*	Duration of HMF Mean [range]	Follow-up/ Time of assessment	Key findings	Protocol for assessment
			Banta-Wright 2012	75 vs. 22	6.8 months	n/r	**Acceptable metabolic control**<br>**No significant difference in metabolic control between HMF and Std IF**<br>Mean blood Phe concentrations in therapeutic target range (120-360 µmol/L):<br>59/75 (79%) in HMF vs. 17/22 (77%) in Std IF (not significant)<br>The distribution in the high mean blood Phe concentration category was similar in both groups (37% of infants)<br>Std IF babies were more likely to have a low mean Phe concentration, especially in the metropolitan area	**Blood Phe:**<br>Every other day to 2x a week
			Lamônica 2012	HMF: 10	5.3 months [1-14]	n/r	n=8: **Acceptable metabolic control**<br>n=2: **Poor metabolic control**. HMF was interrupted and exclusive formula feeding was started<br>Mean blood Phe concentration during HMF:<br>318 ± 90.6 µmol/L<br>Median (range) blood Phe concentration during HMF:<br>312 (192-462) µmol/L<br>8/10 infants (80%) were able to maintain safe blood Phe concentrations (120-360 µmol/L)	**Blood Phe:**<br>Weekly
			O'Sullivan 2013	45 vs. 128	n/r	5 years	**Acceptable metabolic control**<br>**Significantly better metabolic control in HMF vs. Std IF**<br>Longer HMF duration reduced Phe concentrations significantly more than shorter durations	**Blood Phe:**<br>Frequency of monitoring n/r
			Sweeney 2016	HMF: 1	10 months	2 years	**Acceptable metabolic control**<br>Phe concentrations fluctuated particularly in the 2nd year of life reflecting challenges, e.g., communication with the parents and lack of nutritional information for some traditional foods	**Blood Phe:**<br>Weekly

Group of disorders	Condition	N of studies and infants	References	N of infants: HMF vs. Std IF *(if applicable)*	Duration of HMF Mean [range]	Follow-up/ Time of assessment	Key findings	Protocol for assessment
			Köse 2018	25 vs. 16	7.4 months [1-15]	12 months	**Acceptable metabolic control**<br>**Significantly better metabolic control in HMF vs. Std IF**<br><u>Mean serum Phe in HMF vs. Std IF:</u><br>280 vs. 490 µmol/L<br><u>Serum Phe within acceptable range:</u><br>64% in HMF vs. 31.3% in Std IF (significant)<br><u>Low serum Phe concentrations:</u><br>16% in HMF vs. 0% in Std IF<br><u>High serum Phe concentrations:</u><br>20% in HMF vs. 68.7% in Std IF	**Serum Phe:**<br>Monthly (at least)
			Weiss 2020	HMF: 3	n/r	n/r	**Acceptable metabolic control**	**Blood Phe:**<br>Frequency of monitoring n/r
			Zuvadelli 2022	28 vs. 14	7.2 months [0.8-18.8]	12 months	**Acceptable metabolic control**<br>**No significant difference in metabolic control between HMF and Std IF**<br>90.1% of dried blood spots <360 µmol/L (all infants)<br>**No significant difference according to the type of HMF administration method**	**Blood Phe:**<br>Frequency of monitoring n/r<br>Dried blood spot (DBS) and plasma amino acids were obtained
			Rice 2023	16 vs. 23	7.6 months [0.2-24.4]	≤13 months	**Acceptable metabolic control**<br>**No significant difference in metabolic control between HMF and Std IF**<br><u>Mean blood Phe HMF vs. Std IF groups:</u><br>335±54 vs. 357±52 µmol/L (p=0.0705)	**Blood Phe:**<br>Frequency of monitoring n/r
			Guillén-López 2024	181 vs. 202	n/r	2 years	**Acceptable metabolic control**<br>**No significant difference in metabolic control between HMF and Std IF**<br><u>Median [range] blood Phe HMF vs. Std IF groups:</u><br>234 µmol/L [126-409] vs. 198.5 [75-367] (p=0.224)	**Blood Phe:**<br>Frequency of monitoring n/r
			Mohammad zadeh 2024	13 vs. 21	14.3 months [1–24]	<2 years	**Acceptable metabolic control**<br>**No significant difference in metabolic control between HMF and Std IF**<br><u>Mean [range] serum Phe HMF vs. Std IF groups:</u><br>296±223 [121-1150] vs. 228±127 [60-424] µmol/L (p=0.51)	**Serum Phe:**<br>Monthly (at least)

Group of disorders	Condition	N of studies and infants	References	N of infants: HMF vs. Std IF *(if applicable)*	Duration of HMF Mean [range]	Follow-up/ Time of assessment	Key findings	Protocol for assessment
**Other Amino Acid Disorders (AADs)**	*Maple Syrup Urine Disease (MSUD)*	N=4<br>HMF: 6	Touati 2001	HMF: 3	4 months (n=1)<br>11 months (n=1)<br>n/r (n=1)	n/r	**Acceptable metabolic control**<br><u>Mean [median, range] of plasma Val (n=3):</u><br>194±79 µmol/L [158 µmol/L, 35-582 µmol/L]<br><u>Mean [median, range] of plasma Ile (n=3):</u><br>152±113.8 µmol/L [89 µmol/L, 3-473 µmol/L]<br><u>Mean [median, range] of plasma Leu (n=3):</u><br>85±18.2 µmol/L [91 µmol/L, 20-668 µmol/L]<br>Overall, metabolic control was effective and none of the infants had a metabolic decompensation during HMF	**Plasma BCAA:**<br>Weekly (at least)
			Huner 2005	HMF: 1	3.3 months	4 months	**Acceptable metabolic control with expressed HM** (and precursor-free medical formula)<br>At 2 months: on demand HMF introduced instead of expressed HM. **Metabolic control was impaired after 1 week of on demand HMF** (very high plasma BCAA concentrations though no observable clinical problem; HMF was terminated, and the infant was hospitalized)<br>At 3 months: plasma BCAA within therapeutic range, and on demand HMF was introduced again. **Acceptable plasma BCAA concentrations** were maintained but HMF was discontinued at 4 months due to stress of frequent checks	**Plasma BCAA, serum ammonia and urinary ketones, and organic acids:**<br>Weekly or biweekly after discharge
			Ross 2016	HMF: 1	n/r	>14 days	**Acceptable metabolic control with expressed HM** (and precursor-free medical formula)<br>Neonate started on intravenous fluids while waiting for delayed shipment of precursor-free medical formula: high plasma BCAA concentrations before initiation of medical formula. Medical formula started on day 5; no HM to avoid neurological sequelae. Day 8, watery stools. Day 10, skin lesions. Day 12, fever and lethargy. Plasma Val and Leu within therapeutic range but Ile deficiency detected. Expressed HM was added to the feeds after day 14 and Ile concentrations increased to normal	n/r
			Pichler 2017	HMF: 1	1 month	1 month	**Acceptable metabolic control**<br>The infant did not exhibit any problems or did not develop any metabolic crisis during the HMF period	**Plasma amino acids & general biochemical monitoring:**<br>Every 2 weeks

Group of disorders	Condition	N of studies and infants	References	N of infants: HMF vs. Std IF *(if applicable)*	Duration of HMF Mean [range]	Follow-up/ Time of assessment	Key findings	Protocol for assessment
	Homocystinuria (HCU)	N=2<br><br>HMF: 4	Dixon 2014	HMF: 3	3 months (n=1)<br>≥6 months (n=2)	6 months	**Acceptable metabolic control** (if adequate intake of precursor-free medical formula)<br>Median [range] of tHcy:<br>n=1, 110 µmol/L [>90 µmol/L due to inadequate medical formula intake] HMF was stopped 12 weeks into treatment<br>n=2, within acceptable range **(**48 µmol/L [range 21-114] and 37 µmol/L [range 11-76])<br>Overall, plasma tHcy was mostly <50 µmol/L and methionine in acceptable range	**Plasma Hcy (total and free), Met and Cys:**<br>Frequency of monitoring n/r
			Aktuğlu 2015	HMF: 1	≥18 months	18 months	**Acceptable metabolic control**<br>Mean blood Met (93 µmol/L) and Hcy (21 µmol/L) concentrations were within acceptable range (Reference: 9-44 µmol/L and 5-14 µmol/L, respectively)	**Blood Met and Hcy:**<br>Frequency of monitoring n/r
	Hypermethioninemia	N=1<br><br>HMF: 1	Pichler 2017	HMF: 1	12 months	12 months	**Acceptable metabolic control**<br>The infant did not exhibit any problems or did not develop any metabolic crisis during the HMF period	**Plasma amino acids & general biochemical monitoring:**<br>Every 3 months
	Tyrosinemia (TYR)	N=2<br><br>HMF: 3	Daly 2005	HMF: 2	5.1 months (n=1)<br>5.3 months (n=1)	5 months	**Acceptable metabolic control**<br>Median blood Tyr concentration (n=2): 180-465 µmol/L<br>Median Phe concentration (n=2): remained below 45-60 µmol/L	**Blood Tyr and Phe concentrations:**<br>Frequency of monitoring n/r
			Silva 2022	HMF: 1	13 months	14 months	**Acceptable metabolic control**<br>Blood Tyr concentrations consistently within therapeutic target range (<500 µmol/L)<br>Low blood Phe concentrations between 2-7 months of age requiring Phe supplementation<br>Blood Phe concentrations were consistently around minimum Phe concentration until last follow-up<br>At 14 months, biochemical markers were acceptable	**Blood TYR and Phe concentrations:**<br>Weekly
**Organic acidemias (OAs)**	*Methylmalonic acidemia (MMA)*	N=5<br><br>HMF: 14<br>Std IF: 5<br>Total: 19	Dixon 2000	HMF: 2	2 weeks (n=1)<br>11 months (n=1)	2 weeks (n=1)<br>4 months (n=1)	**Acceptable metabolic control in 1 of 2 infants**<br>n=1: during the 4 months of exclusive HMF, the infant was well, and continued on demand HMF up to 11 months<br>n=1: persistent metabolic acidosis and mild hyperammonaemia (120 mmol/L) when on exclusive demand HMF, despite supplementation with precursor-free medical formula she was metabolically unstable and **HMF was discontinued** at 2 weeks of age	**Plasma ammonia:**<br>Frequency n/r

Group of disorders	Condition	N of studies and infants	References	N of infants: HMF vs. Std IF *(if applicable)*	Duration of HMF Mean [range]	Follow-up/ Time of assessment	Key findings	Protocol for assessment
			Huner 2005 & Gökçay 2006	HMF: 4	18.5 months [11-24]	[11-24 months]	**Acceptable metabolic control**<br>n=1 (25%) had low plasma concentrations of Met, Val, Ile, Thr low concentrations of urinary MMA excretion that allowed the cessation of medical formula at 6 months (no metabolic decompensation during HMF),<br>n=2 (50%) had 2-3 hospitalizations (e.g., vomiting, mild acidosis, urinary tract infection) but HMF continued	**Plasma amino acids, serum ammonia and urinary ketones, and organic acids:**<br>Weekly or biweekly after discharge
			Pichler 2017	HMF: 2	9 months (n=1)<br>12 months (n=1)	9 months (n=1)<br>12 months (n=1)	**Acceptable metabolic control**<br>No metabolic crisis during HMF	**Plasma amino acids, ammonia and urine organic acids:**<br>2-4 weeks intervals
			Starin 2024	6 vs. 5	≥12.3 months [≥2-24]	1 year	**Acceptable metabolic control**<br>Direct breastfeeding showed no negative impact on metabolic stability<br>No information reported for infants receiving Std IF as their Phe source	n/r
	Propionic acidemia (PA)	N=5<br><br>HMF: 12<br>Std IF: 8<br>Total: 20	Dixon 2000	HMF: 1	≥4 months	4 months	**Acceptable metabolic control**<br>No episodes of metabolic decompensation and biochemical markers of metabolic control were acceptable	**Plasma ammonia, glutamine, glycine, essential amino acids, blood gas:**<br>Frequency n/r
			Huner 2005 & Gökçay 2006	HMF: 1	3.5 months	4 months	Two metabolic crises (vomiting and mild acidosis; 1 week later, hypotonia and mild encephalopathy). **On demand HMF was stopped**	**Plasma amino acids, serum ammonia and urinary ketones, and organic acids:**<br>Weekly or biweekly after discharge
			Pichler 2017	HMF: 3	12.3 months [6-19]	[6-19 months]	**Acceptable metabolic control**<br>No metabolic crisis during HMF	**Plasma amino acids, ammonia and urine organic acids:**<br>2-4 weeks intervals
			Starin 2024	7 vs. 8	≥8.1 months [1-≥23]	1 year	**Acceptable metabolic control**<br>Direct breastfeeding showed no negative impact on metabolic stability.<br>No information reported for infants receiving Std IF as their Phe source.	n/r

Group of disorders	Condition	N of studies and infants	References	N of infants: HMF vs. Std IF *(if applicable)*	Duration of HMF Mean [range]	Follow-up/ Time of assessment	Key findings	Protocol for assessment
	Glutaric acidemia Type 1 (GA1)	N=4<br>HMF: 6	Huner 2005 & Gökçay 2006	HMF: 2	6 months (n=1)<br>11 months (n=1)	6 months (n=1)<br>11 months (n=1)	**Acceptable metabolic control**<br>During HMF, plasma Lys and Trp concentrations were within the acceptable range and very low amounts of 3-hydroxyglutaric acid were detected in urine in both infants.	**Plasma amino acids, serum ammonia and urinary ketones, and organic acids:**<br>Weekly or biweekly after discharge
			Fitzachary 2015	HMF: 2	≥6 months	6 months	**Acceptable metabolic control**<br>Plasma Lys (99-191 µmol/L) was within reference range in n=1, and low in n=1 (58-84 µmol/L)<br>Plasma Arg and free carnitine were within acceptable reference range in n=2<br>Plasma glutaryl carnitine remained above the reference range in n=1, but within range at follow-up in n=1	**Plasma Lys, Arg, and Trp, free and glutaryl carnitine concentrations:**<br>Frequency of monitoring n/r
			Pichler 2017	HMF: 2	2.5 months (n=1)<br>5 months (n=1)	2.5 months (n=1)<br>5 months (n=1)	**Acceptable metabolic control**<br>n=1 infant had a metabolic crisis at 3 months during a viral infection	**Plasma amino acids, ammonia and urine organic acids:**<br>2-4 weeks intervals
	Isovaleric acidemia (IVA)	N=2<br>HMF: 2	Huner 2005 & Gökçay 2006	HMF: 2	1.5 months (n=1)<br>10 months (n=1)	1.5 months (n=1)<br>10 months (n=1)	**Acceptable metabolic control**<br>No signs of poor metabolic control	**Plasma amino acids, serum ammonia and urinary ketones, and organic acids:**<br>Weekly or biweekly after discharge
	Malonic acidemia (MA)	N=1<br>HMF: 1	Pichler 2017	HMF: 1	6 months	6 months	**Acceptable metabolic control**<br>No metabolic crisis during HMF	**Plasma amino acids, ammonia and urine organic acids:**<br>2-4 weeks intervals
**Urea Cycle Disorders (UCDs)**	*Citrullinemia*	N=1<br>HMF: 1	Kamper 2001	HMF: 1	≥8 months	8 months	**Acceptable metabolic control**<br>The citrulline/glutamine ratio was constantly around 5, and plasma Arg concentrations were kept around 100 µmol/L	**Citrulline/glutamine ratio, plasma Arg concentrations:**<br>Frequency of monitoring n/r

Group of disorders	Condition	N of studies and infants	References	N of infants: HMF vs. Std IF *(if applicable)*	Duration of HMF Mean [range]	Follow-up/ Time of assessment	Key findings	Protocol for assessment
	Ornithine transcarbamylase (OTC) deficiency	N=4<br>HMF: 5	Rawlinson 2000	HMF: 2	7 months (n=1)<br>8 months (n=1)	7 months (n=1)<br>8 months (n=1)	**Acceptable metabolic control**<br>No acute hyperammonaemia during HMF	**Plasma ammonia:**<br>Frequency n/r
			Huner 2005	HMF: 1	1.5 months	1.5 months	**Breastfeeding was stopped owing to inadequate metabolic control** during an episode of respiratory infection and hyperammonemia and was not retried (infant was a severe case and died later during another acute episode at 4 months)	**Plasma amino acids, serum ammonia and urinary ketones, and organic acids:**<br>Weekly or biweekly after discharge
			Pichler 2017	HMF: 1	9 months	9 months	**Acceptable metabolic control**<br>HMF was generally uncomplicated without any case of metabolic crisis	**Plasma amino acids, ammonia and urine organic acids:**<br>2-4 weeks intervals
			Burfield 2024	HMF: 1	3.3 months	5.5 months	**Acceptable metabolic control**<br>Plasma ammonia, glutamine, and branched chain amino acids stable throughout study	**Plasma ammonia, glutamine, and branched chain amino acids:**<br>2-4 weeks intervals
	Arginase deficiency (AD)	N=1<br>HMF: 2	Pichler 2017	HMF: 2	2 months (n=1)<br>6 months (n=1)	2 months (n=1)<br>6 months (n=1)	**Acceptable metabolic control**<br>HMF was generally uncomplicated without any case of metabolic crisis	**Plasma amino acids, ammonia and urine organic acids:**<br>2-4 weeks intervals
	Argininosuccinate lyase (ASL) deficiency	N=2<br>HMF: 4	Dixon 2000	HMF: 2	3 months (n=1)<br>≥4 months (n=1)	3 months (n=1)<br>4 months (n=1)	**Acceptable metabolic control**	**Plasma ammonia, glutamine, arginine, essential amino acids, argininosuccinic acid:**<br>Frequency n/r
			Burfield 2024	HMF: 2	>6.7 months (n=1)<br>>7.6 months (n=1)	6.7 months (n=1)<br>7.6 months (n=1)	**Acceptable metabolic control**<br>Plasma ammonia, glutamine, and branched chain amino acids stable throughout study	**Plasma ammonia, glutamine, and branched chain amino acids:**<br>2-4 weeks intervals

Group of disorders	Condition	N of studies and infants	References	N of infants: HMF vs. Std IF *(if applicable)*	Duration of HMF Mean [range]	Follow-up/ Time of assessment	Key findings	Protocol for assessment
**Fatty Acid Oxidation Disorders (FAODs)**	*Medium-chain acyl-CoA dehydrogenase (MCAD) deficiency*	N=4<br><br>HMF: 48<br>Std IF: 13<br>Total: 61	Roe 1986	HMF: 2	n/r	n/r	**Acceptable metabolic control**<br>Asymptomatic metabolic control was acceptable (except for mild hyperbilirubinemia in n=1), and no metabolic crisis observed	n/r
			Hsu 2008	11 vs. 4	n/r	n/r	**Initial C8 concentrations were significantly higher in HM-fed than in Std IF-fed infants** (80% of infants had C8>10 µM, vs. none in Std IF-fed) probably due to period of relative fasting associated with initiation of breastfeeding,<br>**No significant difference in C8 concentrations and C8/C10 at follow-up between the two groups**	**Plasma C8 and C10 concentrations:**<br>Frequency of monitoring: n/r
			Ahrens-Nicklas 2016	34 vs. 9	n/r	Neonatal period	**C8 concentrations were higher in exclusively HM-fed compared to Std IF fed infants**<br>11 of 24 (46%) exclusively HM-fed infants had signs of metabolic decompensation<br>None of the 19 neonates (0%) who received formula (with or without HMF) became ill	**Plasma C8 concentration:**<br>Frequency of monitoring: n/r
			Petropoulou 2017	HMF: 1	2 months	4 years	**Acceptable metabolic control**<br>Infant remained well on a combination of HMF and Std IF	**n/r**
	Long-chain hydroxyacyl-coA dehydrogenase (LCHAD) deficiency	N=1<br><br>HMF: 3	Pichler 2017	HMF: 3	3.5 months [2.5-5]	[2-5.5 months]	**Acceptable metabolic control**<br>No metabolic crisis was observed	**Acylcarnitines & general biochemical monitoring:**<br>Biweekly
	Very long-chain acyl-CoA dehydrogenase (VLCAD) deficiency	N=1<br><br>HMF: 1	Pichler 2017	HMF: 1	1 month	1 month	**Acceptable metabolic control**<br>No metabolic crisis was observed	**Acylcarnitines & general biochemical monitoring:**<br>Biweekly

Group of disorders	Condition	N of studies and infants	References	N of infants: HMF vs. Std IF *(if applicable)*	Duration of HMF Mean [range]	Follow-up/ Time of assessment	Key findings	Protocol for assessment
	Mixed (LCHAD or VLCAD)	N=1<br>HMF: 4	Hussa 2006	HMF: 4	[1-4 months]	n/r	**Acceptable metabolic control**	n/r
	Carnitine-acylcarnitine translocase (CACT) deficiency	N=1<br>HMF: 1	Kritzer 2020	HMF: 1	>12 months	n/r	**Acceptable metabolic control**<br>No acute decompensations and no episodes of hypoglycaemia, hyperammonaemia, or rhabdomyolysis	**C16, C18:1, CK, ammonia concentrations:**<br>Frequency of monitoring n/r
	Carnitine palmitoyltransferase II (CPT II) deficiency	N=1<br>HMF: 1	Pichler 2017	HMF: 1	n/r	4 years	**Acceptable metabolic control**<br>No metabolic crisis was observed	**Acylcarnitine & general biochemical monitoring:**<br>Biweekly
**Others**	*Galactose epimerase (GALE) deficiency*	N= 1<br>HMF: 2	Pichler 2017	HMF: 2	1 month (n=1)<br>4 months (n=1)	1 month (n=1)<br>4 months (n=1)	**Acceptable metabolic control**<br>No metabolic crisis was observed	**Galactose-1-phosphate concentration in erythrocytes:**<br>Monthly

Abbreviations: Arg, arginine; BCAA, branch-chained amino acids; Cys, cysteine; FU, follow-up; h, hours; HM, human milk; HMF, human milk feeding; Ile, isoleucine; Leu, leucine; Lys, lysine; Met, methionine; Min, minimum; MMA, methylmalonic acid; N, number; n/r, not reported; NBS, newborn screening; Phe, phenylalanine; Std IF, standard infant formula; tHcy, total homocysteine; Thr, threonine; Trp, tryptophan; Tyr, tyrosine; Val, valine.

Supplementary Table 6. Neurodevelopmental outcomes of infants with an inherited metabolic disorder who have received human milk.

Group of disorders	Condition	N of studies and infants	References	N of infants: HMF vs. Std IF *(if applicable)*	Duration of HMF; Mean [range]	Follow-up/ Time of assessment	Key findings	Protocol for assessment
**Amino acid disorders (AADs)**	*Phenylketonuria (PKU)*	N=10<br><br>HMF: 91<br>Std IF: 40<br>Total: 131	Miller 1994	HMF: 1	≥3 months	3 months	**Adequate neurodevelopment**	n/r
			Riva 1996	13 vs. 13	>0.9 months [0.7-1.3]	9.5 years [6.5-12.5]	**Adequate neurodevelopment**<br>**WISC-R verbal and performance scales: significantly better HMF vs. Std IF**<br>After adjustment for differences in social category and maternal education, there was 12.9-point advantage in IQ scoring in the HMF group (p=0.01)<br>The overall IQ score was significantly linked to the type of early (pre-treatment/pre-diagnosis) feeding.	**WISC-R (adapted for 6-16 y):**<br>Overall intellectual capacity (verbal & non-verbal tasks)
			Davidson 2000	33 vs. 19	≤15 months	5 & 10 years	**Adequate neurodevelopment**<br>**Psychological testing: comparable or superior HMF vs. Std IF**	n/r
			Francis 2000	HMF: 1	≥5 months	5 months	**Adequate neurodevelopment**	n/r
			Agostoni 2003	12 vs. 8	≤1 month (n=5)<br>≤2 months (n=7)	5 & 12 months	**Adequate neurodevelopment**<br>Neural performance:<br>**MDI:** No significant difference between groups at 5 and 12 months; slightly decreased from 5 to 12 months in both groups<br>**PDI:** Significantly higher in HMF (Δ=10, 95%CI=0 to 20) only at 5 months; PDI did not change in both groups<br>Visual function:<br>**P100 wave latency** at 15 min arc: Significantly shorter in HMF infants (Δ=–21 ms, 95%CI=–30 to –12 ms); P100 wave latency at 60 min arc: Similar between groups<br>Overall, early LCPUFA intake and status (related to the condition of being either HM-fed or not) was found to be associated with neural and visual performance through the first 12 months of life.	**Neural performance – Bayley Mental Developmental Index (MDI) & Psychomotor Developmental Index (PDI)**:<br>At 5 and 12 months<br><br>**Visual function – P100 wave latency (ms) at 15 min and 60 min of arc**:<br>At 12 months
			Cornejo 2003	HMF: 16	≥6 months (n=14)<br><6 months (n=5) [a]	4 or 6 months	MDI:<br>n=13: **Adequate mental development (MDI>80)**<br>n=3: **Inadequate mental development (MDI<80)**<br>PDI:<br>n=11: **Adequate motor development (PDI>80)**	**Bayley Mental Developmental Index (MDI) & Psychomotor Developmental Index (PDI):**

Group of disorders	Condition	N of studies and infants	References	N of infants: HMF vs. Std IF *(if applicable)*	Duration of HMF; Mean [range]	Follow-up/ Time of assessment	Key findings	Protocol for assessment
							n=5: **Inadequate motor development (PDI<80)**	At 4 months (n=10), at 6 months (n=6)
			Sweeney 2011	HMF: 1	≥16 months	16 months	**Adequate neurodevelopment**	n/r
			Lamônica 2012	HMF: 10	5.3 months [1-14]	n/r	n=8: **Adequate neurodevelopment**<br>n=2: **Inadequate neurodevelopment** (associated with poor metabolic control)	**Early Language Milestone Scale; Basic Steps of Development:** Weekly
			Sweeney 2016	HMF: 1	10 months	3.5 years	**Adequate neurodevelopment**	n/r
			Weiss 2020	HMF: 3	n/r	[2-13 years]	n=2: **Adequate neurodevelopment**<br>n=1: speech development was affected due to cochlea implants and a bilingual education; non-verbal intelligence was on average.	n/r
**Other Amino Acid Disorders (AADs)**	*Maple Syrup Urine Disease (MSUD)*	N=3<br>HMF: 5	Touati 2001	HMF: 3	2 weeks (n=1)<br>11 months (n=1)	n/r	**Adequate psycho-motor development**	n/r
			Huner 2005	HMF: 1	3.3 months	12 months	**Adequate neurodevelopment**	**DDST II**
			Pichler 2017	HMF: 1	1 month	5 years	**Adequate neurodevelopment**	**DDST II**
	Hypermethioninemia	N=1<br>HMF: 1	Pichler 2017	HMF: 1	12 months	5 years	**Adequate neurodevelopment**	**DDST II**
	Tyrosinemia (TYR)	N=1<br>HMF: 1	Silva 2022	HMF: 1	13 months	14 months	**Adequate neurodevelopment**	n/r
**Organic acidemias (OAs)**	*Methylmalonic acidemia (MMA)*	N=4<br>HMF: 7	Dixon 2000	HMF: 1	11 months	4 months	**Adequate neurodevelopment**	n/r
			Huner 2005 & Gökçay 2006	HMF: 4	18.5 months [11-24]	[11-24 months]	n=2: **Adequate neurodevelopment**<br>n=2: **Delayed neurodevelopment** - both infants were late diagnosed with severe neurological impairment at diagnosis; showed improvement while on HMF	**DDST II**

Group of disorders	Condition	N of studies and infants	References	N of infants: HMF vs. Std IF *(if applicable)*	Duration of HMF; Mean [range]	Follow-up/ Time of assessment	Key findings	Protocol for assessment
			Pichler 2017	HMF: 2	9 months (n=1)<br>12 months (n=1)	1 year (n=1)<br>2 years (n=1)	**Adequate neurodevelopment**	**DDST II**
	Propionic acidemia (PA)	N=3<br>HMF:4	Huner 2005 & Gökçay 2006	HMF: 1	3.5 months	4 months	**Adequate neurodevelopment**	**DDST II**
			Pichler 2017	HMF: 3	12.3 months [6-19]	[2-11 years]	**Mild to moderate neurological impairment** in all 3 infants.	**DDST II**
	Glutaric acidemia Type 1 (GA1)	N=3<br>HMF:4	Huner 2005 & Gökçay 2006	HMF: 2	6 months (n=1)<br>11 months (n=1)	6 months (n=1)<br>11 months (n=1)	n=1: **Adequate neurodevelopment**<br>n=1: **Delayed neurodevelopment** - late diagnosed with severe neurological impairment at diagnosis; showed improvement while on HMF	**DDST II**
			Pichler 2017	HMF: 2	2.5 months (n=1)<br>5 months (n=1)	2 years (n=1)<br>4 years (n=1)	n=1: **Adequate neurodevelopment**<br>n=1: **Delayed neurodevelopment**	**DDST II**
	Isovaleric acidemia (IVA)	N=2<br>HMF: 2	Huner 2005 & Gökçay 2006	HMF: 2	1.5 months (n=1)<br>10 months (n=1)	1.5 months (n=1)<br>10 months (n=1)	**Adequate neurodevelopment**	**DDST II**
	Malonic acidemia (MA)	N=1<br>HMF: 1	Pichler 2017	HMF: 1	6 months	1 years	**Adequate neurodevelopment**	**DDST II**
**Urea Cycle Disorders (UCDs)**	*Citrullinemia*	N=1<br>HMF: 1	Kamper 2001	HMF: 1	≥8 months	8 months	**Adequate psycho-motor development**	n/r
	Ornithine transcarbamylase (OTC) deficiency	N=1<br>HMF: 1	Pichler 2017	HMF: 1	9 months	6 years	**Adequate neurodevelopment**	**DDST II**
	Arginase deficiency (AD)	N=1<br>HMF: 2	Pichler 2017	HMF: 2	2 months (n=1)<br>6 months (n=1)	3 years (n=1)<br>10 years (n=1)	**Adequate neurodevelopment**	**DDST II**
	Argininosuccinate lyase (ASL) deficiency	N=1<br>HMF: 1	Dixon 2000	HMF: 1	3 months (n=1)<br>≥4 months (n=1)	3 months (n=1)<br>4 months (n=1)	**Adequate neurodevelopment**	n/r
	Carbamoyl phosphate synthetase 1 (CPS 1) deficiency	N=1<br>HMF: 2	Bzduch 2019	HMF: 2	2 months (n=1)<br>3 months (n=1)	1.5 years (n=1)<br>3 years (n=1)	**Adequate psycho-motor development**	n/r

Group of disorders	Condition	N of studies and infants	References	N of infants: HMF vs. Std IF *(if applicable)*	Duration of HMF; Mean [range]	Follow-up/ Time of assessment	Key findings	Protocol for assessment
**Fatty Acid Oxidation Disorders (FAODs)**	*Medium-chain acyl-CoA dehydrogenase (MCAD) deficiency*	N=1<br>HMF: 11<br>Std IF: 4<br>Total: 15	Hsu 2008	11 vs. 4	n/r	n/r	**Adequate neurodevelopment in all infants, except one** HM-fed infant who had neonatal hypoglycemia, and mild expressive speech delay at 2 years of age (improving with therapy).	n/r
	Long-chain hydroxyacyl-coA dehydrogenase (LCHAD) deficiency	N=1<br>HMF: 3	Pichler 2017	HMF: 3	3.5 months [2.5-5]	[0.5-10 years]	**Adequate neurodevelopment**	**DDST II**
	Very long-chain acyl-CoA dehydrogenase (VLCAD) deficiency	N=1<br>HMF: 1	Pichler 2017	HMF: 1	1 month	6 months	**Adequate neurodevelopment**	**DDST II**
	Mixed (LCHAD or VLCAD)	N=1<br>HMF: 4	Hussa 2006	HMF: 4	[1-4 months]	n/r	**Adequate neurodevelopment**	n/r
	Carnitine-acylcarnitine translocase (CACT) deficiency	N=1<br>HMF: 1	Kritzer 2020	HMF: 1	>12 months	n/r	**Adequate neurodevelopment**<br>(despite early severe medical complications, patient demonstrated normal gross motor, fine motor, speech, and social milestones).	n/r
	Carnitine palmitoyltransferase II (CPT II) deficiency	N=1<br>HMF: 1	Pichler 2017	HMF: 1	n/r	4 years	**Adequate neurodevelopment**	**DDST II**
**Others**	*Galactose epimerase (GALE) deficiency*	N=1<br>HMF: 2	Pichler 2017	HMF: 2	1 months (n=1)<br>4 months (n=1)	9 years (n=1)<br>5 years (n=1)	**Adequate neurodevelopment**	**DDST II**

Abbreviations: DDST II, Denver Developmental Screening Test; HMF, human milk feeding; IMD, inherited metabolic disorders; Min, minimum; N, number; n/r, not reported; PKU, phenylketonuria; Std IF, standard infant formula; WISC-R, The Wechsler Intelligence Scale for Children-Revised.

[a] Cornejo 2003: 19 infants were breastfed; however, psychomotor development was assessed in 16 infants only. Individual breastfeeding durations were not available; hence, the duration for the 19 infants is reported in this table.

Supplementary Table 7. Psychological outcomes associated with human milk feeding in mothers of infants with an inherited metabolic disorder.

Group of disorders	Condition	N of studies and infants	References	N of mothers providing HMF vs. Std IF	Duration of HMF Mean [range]	Follow-up/ Time of assessment	Key findings	Protocol for assessment
**Amino acid disorders**	*Phenylketonuria (PKU)*	N=5<br><br>HMF: 92<br>Std IF: 25<br>Total: 117	Francis 1981	HMF: 6	n/r	n/r	Bonding between mother and child was reported to be more secure as a result of breastfeeding.	**n/r**
			Miller 1994	HMF: 1	3 months	3 months	The mother reported a rewarding HMF experience.	n/r
			Nielsen 2005	HMF: 6	n/r	n/r	Continuing HMF was reported to have a very positive influence on the mother-child relationship.	n/r
			Schulpis 2019	HMF: 54	1 month	1 month	Mothers on HMF only experienced the highest degree of stress when asked for breastfeeding replacement than those who were on HMF and formula feeding at baseline; psychological support helped all mothers to feel better or lowered signs of stress.	**Stress Symptom Checklist**
			Schulpis 2021	25 vs. 25	PKU:<br>6 days (until diagnosis)<br><br>HPA:<br>n/r	n/r	The percentage of mothers experiencing high or severe stress was significantly higher in mothers of PKU infants on exclusive formula feeding compared to mothers of HPA infants on partial HMF (36% vs. 12%).<br>Mothers in both groups were relieved from stress symptoms (0% high or severe stress) after psychological support (five sessions, once per week).	**Stress Symptom Checklist**
**Organic acidemias**	*Methylmalonic acidemia (MMA)*	N=1<br><br>HMF: 6<br>Std IF: 5<br>Total: 11	Starin 2024	6 vs. 5	≥12.3 months [≥2-24]	1 year	Mothers reported improved mental well-being and a strengthened connection with their child when allowed to breastfeed.	n/r
	Propionic acidemia (PA)	N=1<br><br>HMF: 7<br>Std IF: 8<br>Total: 15	Starin 2024	7 vs. 8	≥8.1 months [1-≥23]	1 year	Mothers reported improved mental well-being and a strengthened connection with their child when allowed to breastfeed.	n/r

Abbreviations: HMF, human milk feeding; N, number; n/r, not reported; Std IF, standard infant formula.

Supplementary Table 8. Quality appraisal and risk of bias assessment of controlled intervention studies.

Study (Author, Year)	Items of "NIH Quality Assessment of Controlled Intervention Studies"														Overall
	1	2	3	4	5	6	7	8	9	10	11	12	13	14	
McCabe 1989	-	NA	-	-	?	+	?	?	+	?	+	-	+	NA	Fair
Greeve 1994	-	NA	-	-	?	+	-	-	+	-	+	-	+	NA	Poor
Agostoni 2003	-	NA	-	-	?	+	+	+	?	?	+	+	+	+	Fair
van Rijn 2003	-	NA	-	-	?	+	NA	NA	+	?	+	-	+	NA	Fair
Kanufre 2007	-	NA	-	-	?	+	+	+	+	-	+	-	+	+	Fair
Hsu 2008	-	NA	-	-	?	?	NA	NA	?	?	+	-	+	NA	Poor
Ahrens-Nicklas 2016	-	NA	-	-	?	+	NA	NA	?	?	+	-	+	NA	Poor

Each item was rated as low risk ("yes" = +), unclear ("cannot determine/not reported" = ?), or high risk ("no" = -) for the following type of bias: description as randomized study (1); adequacy of randomization (2); allocation concealment (3); blinding of study participants and providers to treatment group assignment (4); blinding of outcome assessors (5); similarity of groups at baseline (6); overall drop-out rate ≤20% (7); differential drop-out rate ≤15% (8); adherence to the intervention (9); avoidance of other interventions (10); measurement of outcomes (valid, reliable, consistent) (11); power calculation (12); prespecified outcomes (13); intention-to-treat analysis (14). NIH, National Institute of Health; NA, not applicable.

Supplementary Table 9. Quality appraisal and risk of bias assessment of case reports and case series.

Study (Author, Year)	Items of "NIH Quality Assessment of Quality Assessment Tool for Case Series Studies"									Overall
	1	2	3	4	5	6	7	8	9	
Francis 1980	-	-	?	?	+	-	+	?	-	Poor
Roe 1986	-	-	+	+	-	+	?	?	+	Poor
Miller 1994	-	+	NA	NA	+	-	+	NA	-	Poor
Duncan 1997	-	+	NA	NA	+	+	+	NA	+	Fair
Dixon 2000	-	+	?	-	+	-	+	?	-	Poor
Francis 2000	-	-	NA	NA	+	-	+	NA	-	Poor
Rawlinson 2000	-	-	?	+	+	-	+	?	-	Poor
Kamper 2001	-	+	NA	NA	+	-	+	NA	+	Fair
Touati 2001	+	-	?	?	+	?	+	?	-	Poor
Daly 2005	+	+	?	+	+	+	+	?	+	Fair
Nielsen 2005	-	-	+	+	+	-	?	?	-	Poor
Hussa 2006	-	-	?	?	+	-	+	?	-	Poor
Sweeney 2011	-	+	NA	NA	+	-	+	NA	-	Poor
Dixon 2014	+	-	?	+	+	-	+	?	-	Poor
Aktuğlu 2015	-	+	NA	NA	+	+	+	NA	+	Fair
Fitzachary 2015	+	-	?	+	+	-	+	?	-	Poor
Ross 2016	-	+	NA	NA	+	+	+	NA	+	Fair
Sweeney 2016	+	+	NA	NA	+	-	+	NA	-	Poor
Petropoulou 2017	+	+	NA	NA	+	+	+	NA	+	Fair
Bzduch 2019	-	+	+	+	+	-	+	?	-	Poor
Kritzer 2020	+	+	NA	NA	+	+	+	NA	+	Fair
Weiss 2020	+	+	?	+	+	+	?	?	+	Fair
Silva 2022	+	+	NA	NA	+	+	+	NA	+	Fair

Each item was rated as low risk ("yes" = +), unclear ("cannot determine/not reported" = ?), or high risk ("no" = -) for the following type of bias: clear research question/objective (1); description of the study population/case definition (2); consecutive cases (3); comparable subjects (4); description of the intervention (5); measurement of outcomes (valid, reliable, consistent) (6); adequate duration of follow-up (7); description of statistical methods (8); description of the results (9). NIH, National Institute of Health; NA, not applicable.

Supplementary Table 10. Quality appraisal and risk of bias assessment of observational cohort and cross-sectional studies.

Study (Author, Year)	Items of "NIH Quality Assessment Tool for Observational Cohort and Cross-Sectional Studies"														Overall
	1	2	3	4	5	6	7	8	9	10	11	12	13	14	
Riva 1996	+	+	+	?	-	+	?	-	+	-	+	?	NA	+	Fair
Motzfeldt 1999	-	+	+	?	-	+	?	-	?	+	-	?	NA	-	Poor
Davidson 2000	-	+	+	?	-	+	+	-	+	+	-	?	NA	-	Poor
Cornejo 2003	+	-	?	?	-	+	+	-	+	+	+	?	NA	-	Fair
Huner 2005	+	-	?	?	-	+	+	-	+	+	-	?	?	NA	Poor
Gökçay 2006	-	-	?	?	-	+	+	-	+	+	+	?	?	NA	Poor
Sweeney 2009	+	+	+	?	-	+	+	-	+	+	-	?	NA	-	Poor
Santos 2011	+	-	?	+	?	+	+	-	+	+	+	?	?	-	Fair
Banta-Wright 2012	+	+	+	?	-	+	+	-	+	+	+	?	NA	-	Fair
Lamônica 2012	+	+	?	?	-	+	+	-	+	+	+	?	+	+	Fair
O'Sullivan 2013	+	+	+	?	-	+	+	+	+	+	+	?	NA	-	Fair
Pichler 2017	+	+	?	+	-	+	+	-	?	+	+	?	NA	-	Poor
Köse 2018	+	+	-	+	-	+	+	-	+	+	+	-	-	+	Fair
Schulpis 2019	+	-	+	?	-	+	+	+	+	NA	+	?	NA	-	Fair
Schulpis 2021	+	-	?	?	-	+	?	+	+	+	+	?	NA	-	Fair
Zuvadelli 2022	+	+	?	+	-	+	+	-	+	+	+	?	NA	-	Fair
Rice 2023	+	+	+	+	-	+	+	-	+	+	+	?	NA	-	Fair
Rocha 2023	+	+	?	+	-	+	+	+	+	NA	+	?	NA	-	Fair
Burfield 2024	+	+	?	+	-	+	+	-	+	+	+	?	+	NA	Poor
Guillén-López 2024	+	-	?	?	?	+	?	-	-	+	+	?	NA	-	Poor
Mohammadzadeh 2024	+	+	-	+	-	+	+	-	+	+	+	?	NA	+	Fair
Starin 2024	+	-	?	?	?	+	+	-	-	+	-	?	NA	NA	Poor

Each item was rated as low risk ("yes" = +), unclear ("cannot determine/not reported" = ?), or high risk ("no" = -) for the following type of bias: clear research question/objective (1); specification of the study population (2); participation rate (3); homogeneous population (4); sample size justification, power description, effect estimate (5); exposure assessed prior to outcome measurement (6); Sufficient timeframe for follow-up (7); different levels of the exposure of interest (8); exposure measures and assessment (9); repeated exposure assessment (10); measurement of outcomes (valid, reliable, consistent) (11); blinding of outcome assessors (12); follow-up rate (13); statistical analyses and adjustment for key confounders (14). NIH, National Institute of Health; NA, not applicable.

Supplementary Table 11. Recommendations on human milk feeding of infants with an inherited metabolic disorder, in published guidelines or consensus reports.

Disorder	Condition	Guideline	Recommendations on HMF:
Aminoacidopathies	PKU	**Singh et al., 2014 (USA)**	"Include breast milk and/or infant formula as sources of Phe in the diet of an infant with PAH deficiency as there is agreement that feeding at the breast, as well as feeding expressed breast milk by bottle, results in good metabolic control. Later in the first year, breast milk or infant formula can be slowly removed in exchange for limited amounts of intact protein from solid foods containing an equivalent amount of Phe."
		**van Wegberg et al., 2017 (Europe)**	**Statement #34: Grade C**<br>"In infants with PKU, breastfeeding in combination with a Phe-free infant L-AA formula should be encouraged. It is associated with long-term satisfactory blood Phe control and growth."
		**Inwood et al., 2017 (Australasia)**	"Consistent with recommendations for healthy infants, breastfeeding is encouraged for infants with PKU (NHMRC 2012; Ministry of New Zealand 2012). Consultation with midwives or lactation consultants may be of benefit for some mothers who experience breastfeeding problems."
	MSUD	**Frazier et al., 2014**	**Acute dietary treatment - Rating:** Consensus; **Clinical application:** Imperative<br>"Consider use of breast milk (mean Leu concentration of 1 mg/mL) as a source of intact protein (and BCAA) in the dietary management of infants with MSUD if there is frequent anthropometric, clinical, and laboratory monitoring of the infant and mother has adequate milk production."
	TYR 1	**de Laet et al., 2013**	No recommendation on HMF.
		**Chinsky et al., 2017**	"Introduction of regular infant formula or breast milk should be adjusted to achieve 185–550 mg/day of Phe and 95–275 mg/day of tyrosine."
	CBS deficiency	**Morris et al., 2017**	**Under Statement #22:** Methionine restriction; **Grade of recommendation:** D<br>The authors referred to the UK's "Homocystinuria (HCU) Dietetic Management Pathway" as a potential management algorithm.<br>"For infants diagnosed through NBS in the UK, the supply of Met/natural protein (from breast milk or formula) is stopped (after the pyridoxine test) and a Met-free complete infant formula is given for 2-4 days to reduce Hcy levels. Met in the form of breast milk or infant formula is then introduced, divided into several feeds, in conjunction with the Met-free formula. UK guidelines recommend a starting allowance of 90-120 mg Met/day (or 30 mg/kg/day if weight <3 kg). The Met allowance is then titrated against the patient's plasma tHcy levels. Weaning should begin at the usual time and progress through stages as normal - Gradually the Met allowance of breast milk or standard formula is replaced with protein/Met containing foods. Age appropriate concentrated L-AA supplements are introduced at appropriate times to ensure full protein requirements are met."
Organic acidemias	GA1	**Boy et al., 2023**	"*Infant feeding*: Breastmilk is physiological and beneficial for infants (…). Breastmilk feeding in infants with GA1 is used worldwide and should be encouraged. The guideline development group is mostly experienced in breastmilk feeding on demand after administration of a lysine-free and tryptophan-reduced AAM thus limiting lysine intake in analogy to PKU. This procedure has been used in several trials and is associated with beneficial clinical outcome. Clinical experience with administration of AAM after breastmilk feeding is limited. Since the amount of lysine in breast milk (86 mg/100 ml) and formula milk used for bottle feeding are known, daily lysine intake can be easily calculated."
	PA	**Jurecki et al., 2019**	**Strength of evidence:** Fair; **Clinical Action:** Imperative<br>"Human breast milk (from feeding at the breast or using expressed breastmilk) can be used as source of intact protein with careful monitoring for infants with PA."
	MMA and PA	**Forny et al., 2021** ***(first revision of Baumgartner et al., 2014)***	**Recommendation #7. Outcome:** Metabolic stability; **Quality of evidence**: low; **Strength of recommendation:** weak<br>"We suggest a low natural protein diet under consideration of age-appropriate total protein requirements to improve metabolic stability."<br>"Breastfeeding is possible considering the total natural protein intake."
		**Baumgartner et al., 2014**	**Practical aspects of dietary management (under Statement #37. Grade of recommendation: C-D)**<br>"There are few published reports of successful demand breast feeding in MMA/PA and some do not advocate this in MMA/PA. Expressed breast milk should be encouraged if demand breast feeding is impracticable. For MMA/PA particular breast milk advantages include its low protein and amino acid content, protection against infection, and reduction in gut propionate."

Disorder	Condition	Guideline	Recommendations on HMF:
**Urea cycle disorders**	**CPS1D, OTCD, ASSD, ASLD, ARG1D, NAGSD, ORNT1D**	**Häberle et al., 2012 & 2019**	**Practical aspects of dietary management of low-protein diet (under Statement #22. Grade of recommendation: C)**<br>"The main protein source for infants should be either breast feeding or standard infant formula. Exclusive demand breast feeding is possible but this needs close analytical/clinical monitoring, and if necessary protein intake can be limited by giving protein-free infant formula prior to breast feeds."
**Fatty acid oxidation disorder**	**VLCADD**	**Van Calcar et al., 2020**	**Strength of evidence:** Consensus; **Clinical action:** Conditional<br>"For neonates with suspected VLCAD, initiate fasting precautions while awaiting confirmation of the diagnosis. Asymptomatic neonates can continue to breast feed (or feed expressed breast milk) without MCT supplementation, if appropriate fasting precautions are followed."<br><br>**Strength of evidence:** Consensus; **Clinical action:** Conditional<br>"Support breastfeeding of infants with VLCAD, taking into consideration the following:<br>• For an asymptomatic infant with a MILD form of VLCAD, allow breastfeeding (or expressed breast milk) without MCT, as long as breast milk supply remains adequate, age appropriate weight gain is maintained, and fasting recommendations are followed;<br>• For an asymptomatic infant with a MODERATE form of VLCAD, allow breastfeeding (or expressed breast milk) but consider supplementing breast milk with a low LCF, high MCT medical food;<br>• For an asymptomatic infant with a SEVERE form of VLCAD, the primary source of nutrition should be a low LCF, high MCT medical food;<br>• For a symptomatic infant, depending on the severity of symptoms and lab monitoring, consider allowing some breast milk while using a low LCF, high MCT medical food to meet energy needs;<br>If breastfeeding was discontinued during metabolic decompensation, consider reintroduction of partial breast feeding (or expressed breast milk) after the infant returns to an asymptomatic clinical state, if the mother's breast milk supply remains adequate to do so."
**Disorders of carbohydrate metabolism**	**Classical galactosemia**	**Welling et al., 2017**	**Recommendation #4 (++):**<br>"Clinicians should immediately commence a galactose restricted diet (discontinuation of breast milk or whey-based infant formulas and initiation of a soy-based, casein hydrolysate or elemental formula) if classical galactosemia is suspected in an infant, without waiting for confirmation of the diagnosis."
	**Glycogen storage disease Type III**	**Kishnani et al., 2010**	No recommendation on HMF.
	**Glycogen storage disease Type I**	**Kishnani et al., 2014**	No recommendation on HMF.

Abbreviations; AAM, amino acid mixture; ALT, alanine aminotransferase; ARG1D, arginase 1 deficiency; ASLD, argininosuccinate lyase deficiency; ASSD, argininosuccinate synthetase deficiency; AST, aspartate aminotransferase; BCAA, branch chained amino acids; CBS, cystathionine β-synthase; CK, creatine kinase; CPS1D, carbamoylphosphate synthetase 1 deficiency; GA 1, glutaric aciduria type 1; Hcy, homocysteine; HMF, human milk feeding; L-AA, L-amino acids; LCF, long chain triglyceride formula; Leu, leucine; MCT, medium chain triglycerides; Met, methionine; MMA, methylmalonic acidemia; MSUD, maple syrup urine disease; NAGSD, N-acetylglutamate synthase deficiency; ORNT1D, deficiency of the mitochondrial ornithine/citrulline antiporter; OTCD, ornithine transcarbamylase deficiency; PA, propionic acidemia; PAH, phenylalanine hydroxylase; Phe, phenylalanine; PKU, phenylketonuria; TYR 1, tyrosinemia type 1; VLCADD, very long-chain acyl-CoA dehydrogenase deficiency.
